# Ultrafine Ru‐M Alloy@S Vacancy‐Rich MoS_2_ Nanosheets With Bimetallic Active Sites for Efficient Hydrogen Evolution in Alkaline and Acidic Media

**DOI:** 10.1002/advs.202519323

**Published:** 2025-11-28

**Authors:** Rui Su, Tiantian Ding, Hao Tang, Xueying Yang, Jie Zhang, Xiaolin Liu, Jinfu Jia, Yuan Liu, Jin Ge, Zhilin Wu, Zhengya Dong, Xiaojing Zhu

**Affiliations:** ^1^ MOE Laboratory of Bioinorganic and Synthetic Chemistry GBRCE for Functional Molecular Engineering LIFM, IGCME School of Chemistry Sun Yat‐Sen University Guangzhou 510006 China; ^2^ Chemistry and Chemical Engineering Guangdong Laboratory Shantou 515031 China; ^3^ College of Chemistry and Chemical Engineering Key Laboratory for Preparation and Application of Ordered Structural Materials of Guangdong Province Shantou University Shantou 515063 China; ^4^ School of Chemistry South China Normal University Guangzhou 510006 China

**Keywords:** Bimetallic active sites, HER, S vacancy‐rich MoS_2_ nanosheets, Ultrafine Ru‐based alloy, Ultrasonic microreactor

## Abstract

Developing efficient, scalable electrocatalysts for the hydrogen evolution reaction (HER) is crucial for industrial H_2_ production. Herein, ultrafine (≈1.7 nm) ruthenium‐cobalt (RuCo) alloys anchored on Sulfur‐vacancy‐rich 1T phase molybdenum disulfide (1T phase MoS_2‐x_) nanosheets (denoted as Ru_2_Co_1_‐4@E‐MoS_2‐x_) are synthesized via ultrasonic microreactor (USMR) technology. The USMR strategy concurrently achieves a reduced alloy size, accelerated Ru reduction kinetics, and improved metal dispersion, endowing the resulting catalysts with high metal loading and abundant accessible active sites. Experimental and theoretical analyses reveal that Co incorporation optimizes RuCo electronic structure and strengthens interfacial coupling with 1T phase MoS_2‐x_, lowering the Gibbs free energy of H^*^ adsorption (ΔG_H*_) on both Ru and Co sites. This synergistic interaction establishes bimetallic active centers that overcome the adsorption–desorption trade‐off in single‐metal systems. In situ Raman spectroscopy further confirms that Co promotes water dissociation and hydrogen desorption at Ru sites under alkaline conditions. Consequently, Ru_2_Co_1_‐4@E‐MoS_2‐x_ exhibits exceptional HER activity, achieving record‐low overpotentials of 31 mV in acidic and 36 mV in alkaline media at 10 mA·cm^−2^, significantly outperforming 20 wt% Pt/C (45 and 53 mV, respectively). Moreover, the USMR approach is universal, generating highly active RuM@E‐MoS_2‐x_ catalysts (M = Fe, Ni, Cu). This work establishes a novel “bimetallic active sites/engineered carrier” paradigm for advanced electrocatalytic water splitting.

## Graphical abstract

A USMR synthesizes ultrafine RuCo alloy on S‐vacancy‐rich 1T phase MoS2. This method enables rapid precursor mixing, instantaneous reduction, and uniform alloy dispersion while creating S vacancies. The synergy between Ru‐Co sites and defective MoS2 optimizes hydrogen kinetics, achieving a low overpotential of 31 mV@10 mA·cm‐2 in acid, providing a scalable route for high‐performance dual‐site electrocatalysts.

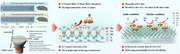

## 1 Introduction

As a clean energy source characterized by high energy density and environmental friendliness, hydrogen fuel can mitigate both the growing energy demand and environmental issues associated with fossil fuels.^[^ ^1^ ^]^ Electrocatalytic water splitting offers an efficient approach to sustainable hydrogen production.^[^ ^2^, ^3^ ^]^ Although platinum (Pt) is used industrially due to its ultra‐low overpotential, its high cost and scarcity hinder large‐scale application.^[^ ^4^ ^]^ Metal ruthenium (Ru) has a similar metal‐hydrogen (M‐H) bond and strength as Pt, and its price is lower than that of metal Pt, making it an effective alternative to Pt.^[^ ^3^, ^5^, ^6^ ^]^ However, the strong interaction between Ru and H^*^ leads to the slow desorption of H^*^, which limits the HER performance of the catalyst.^[^ ^7^ ^]^ Alloying Ru with transition metals (M = Fe, Co, Ni), which have weaker M–H bonds, can modulate the electronic structure via d–d orbital interactions and enhance both catalytic performance and acid resistance.^[^ ^8^, ^9^, ^10^, ^11^, ^12^, ^13^ ^]^

Interface engineering provides a viable route to enhance catalytic performance while minimizing the use of precious metals. By constructing heterogeneous interfaces, supported catalysts leverage synergistic effects to optimize material properties.^[^ ^14^, ^15^, ^16^ ^]^ Selecting a suitable support to tune the adsorption energy of H^*^/OH^*^ on Ru is thus crucial. Molybdenum disulfide (MoS_2_), particularly its metallic 1T phase, exhibits high conductivity and favorable ΔG_H*_ near zero at unsaturated edge sites, facilitating electron transfer and HER kinetics.^[^ ^17^, ^18^, ^19^, ^20^ ^]^ In addition, the study by Xu et al. showed that the adsorption energy of OH^*^ on 1T phase MoS_2_ is greater than that on Ru. Therefore, OH^*^ generated by hydrolysis on Ru active sites tends to transfer from Ru sites to 1T phase MoS_2_.^[^ ^21^ ^]^ This reduces the poisoning of Ru active sites by OH^*^ and releases Ru active sites. Therefore, 1T phase MoS_2_ as a carrier to load RuM alloy is expected to exhibit strong HER catalytic activity.

Current synthetic strategies for metal particle catalysts supported on 1T phase MoS_2_ face severe challenges in precisely controlling interfacial electronic coupling and optimizing active site exposure. Impregnation methods usually involve immersing the support in a metal precursor solution, followed by reduction. Due to the lack of in situ reduction and strong interfacial bonding mechanisms, they often result in weak metal‐support interactions and insufficient interfacial bonding. In the subsequent reduction or calcination steps, the metal particles tend to undergo significant agglomeration, resulting in uneven particle distribution and large size. For example, the RuCo alloy particles in the MXene@RuCo NPs prepared by Li et al. were approximately 20 nm in size.^[^ ^22^ ^]^ Although hydrothermal synthesis can promote good crystallinity, its reaction kinetics are slow, and the mass transfer efficiency is low. Prolonged high‐temperature and high‐pressure environments are prone to Ostwald ripening and particle agglomeration, resulting in a wide size distribution of the final nanoparticles and poor control over the final nanoparticle size.^[^ ^23^, ^24^, ^25^, ^26^, ^27^, ^28^ ^]^ Fu et al. prepared RhCu alloys with an average particle size of 62 nm using ultrasound‐assisted hydrothermal reaction.^[^ ^29^ ^]^ To address these challenges, we propose an innovative strategy for synthesizing RuM@1T‐MoS_2_ heterostructures using ultrasound‐assisted microreactor technology in a controllable manner. The core advantage of this technology lies in the acoustic‐microfluidic synergistic effect^[^ ^30^, ^31^ ^]^: (1) The microfluidic confinement effect is used to achieve excellent substance mixing for ensuring the formation of ultrasmall RuM alloy nanoparticles (2–3 nm) with a narrow size distribution. (2) The extreme conditions and shock waves generated by the ultrasonic cavitation effect can accelerate mass transfer, improve reaction efficiency, and disperse metal particles on the carrier.^[^ ^30^, ^31^, ^32^, ^33^, ^34^, ^35^, ^36^, ^37^ ^]^ (3) In situ generation of covalent S‐M (M = Ru/Fe/Co/Ni) bonds at the metal‐support interface by instantaneous pressure enhances electron transfer kinetics and structural stability. The continuous flow microreactor system can also precisely control the nucleation/growth kinetics through adjustable residence time and multiphase mixing efficiency, overcoming the limitations of batch synthesis in the scale‐up process. This method not only solves the key metal particle size problem in traditional catalysts but also establishes a general platform for the preparation of support‐loaded metal systems.

Based on the motivations, we utilized the UMSR technique to achieve in‐situ reconstruction of S‐vacancy‐rich 1T phase MoS_2_ (E‐MoS_2‐x_ NSs) and efficient loading of ultrafine RuCo alloy (1.7 nm). In this process, USMR achieves threefold impacts: (1) drastically reducing the alloy particle size to ≈1.7 nm, (2) enhancing Ru reduction kinetics through cavitation‐induced local heating, and (3) promoting superior dispersibility of metal alloys on the support via ultrasonic microjet agitation. Experimental results show that at a current density of 10 mA·cm^−2^, Ru_2_Co_1_‐4@E‐MoS_2‐x_ NSs exhibit better overpotentials than Pt/C catalysts in both acidic and alkaline solutions. The improved performance is due to the introduction of Co, which increases its active sites. At the same time, there is a strong interfacial interaction between the high‐conductivity 1T phase E‐MoS_2‐x_ NSs and the RuCo alloy. The transfer of electrons between the interfaces is promoted, so that the RuCo alloy on Ru_2_Co_1_‐4@E‐MoS_2‐x_ NSs exhibits bimetallic active sites, and the ΔG_H*_ of the bimetallic sites is closer to the theoretical value. In addition, the S‐vacancy‐rich 1T phase E‐MoS_2‐x_ NSs also expose more edge active sites, which promotes the dispersed anchoring of alloy nanoparticles. XPS, in situ Raman, and DFT results reveal that the electronic interaction between Ru and Co is strong, causing the Ru 3d orbital to shift negatively. This leads to a downward shift of the d‐band center, optimizing Ru‐H binding strength and promoting H_2_ desorption. As a result, Ru_2_Co_1_‐4@E‐MoS_2‐x_ NSs exhibit excellent HER performance in both acidic and alkaline solutions. In addition, this synthesis strategy was further expanded to synthesize Ru_2_M_1_‐4@E‐MoS_2‐x_ NSs (M = Fe, Ni, Cu). Their HER activities are better than Ru@E‐MoS_2‐x_ NSs, indicating that the HER performance of the synergistic system of “dual metal active sites‐interface engineering carriers” is better than that of the “single metal active sites‐interface engineering carriers” system.

## 2 Results and Discussion

The exfoliated 1T phase MoS_2_ nanosheets (E‐MoS_2_ NSs) with an ultrathin structure of approximately 5‐9 layers were successfully synthesized through ultrasonic treatment of hydrothermally grown MoS_2_ nanoflowers. Comprehensive characterization using transmission electron microscopy (TEM), Atomic Force Microscope (AFM), X‐ray diffraction (XRD), and Raman spectroscopy (Figures S1–S5, Supporting Information) confirmed both the phase transition to metallic 1T structure and the reduced layer thickness, as detailed in Note S1 (Supporting Information). Remarkably, the optimized E‐MoS_2_ NSs demonstrated favorable hydrogen evolution reaction (HER) performance, achieving a low overpotential of ‐195 mV at a current density of 10 mA·cm^−2^.

**Figure 1:**
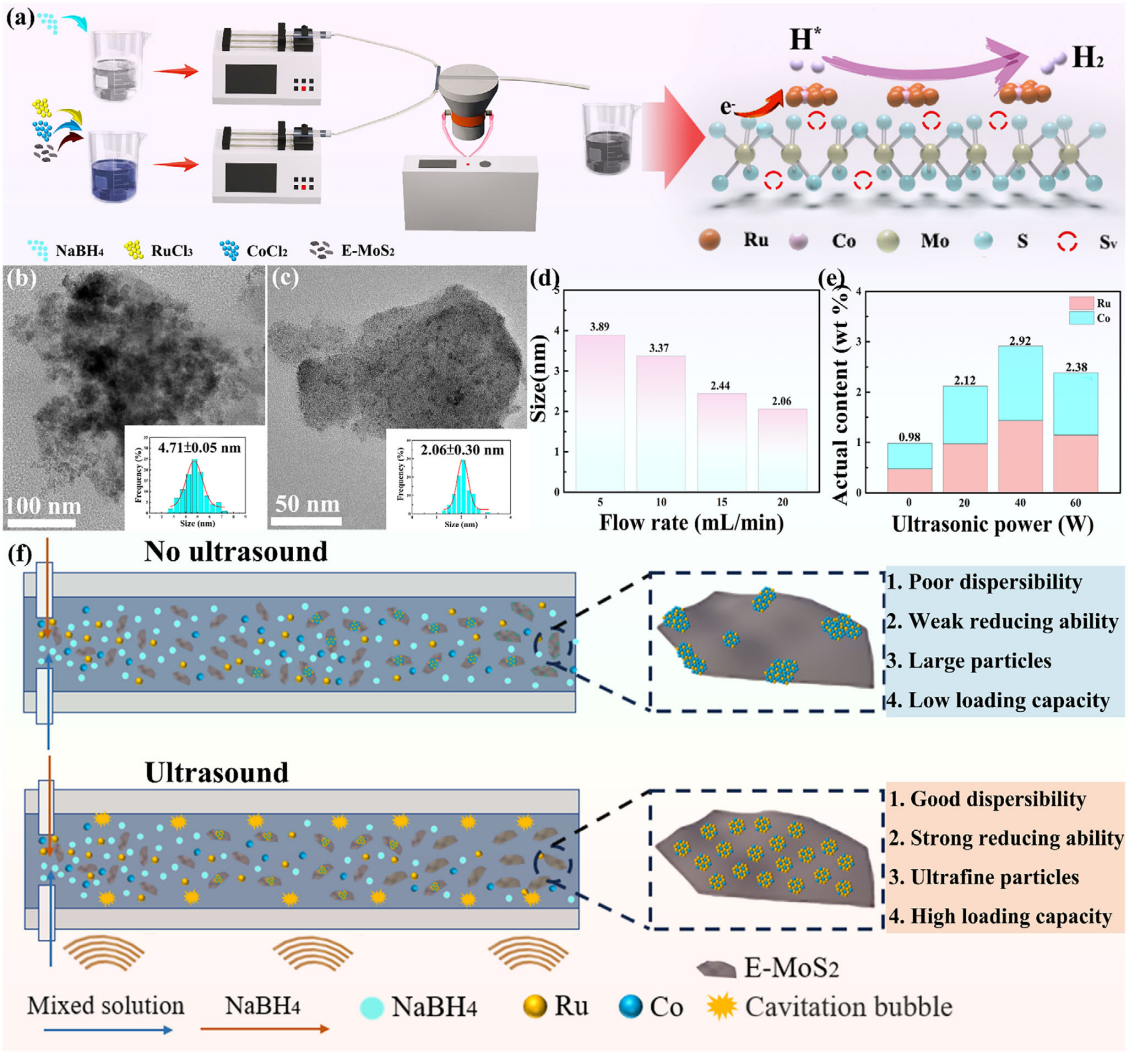
a) Schematic illustration of the synthetic process of the RuCo@E‐MoS_2‐x_ NSs. TEM images of Ru_1_Co_1_‐1@E‐MoS_2‐x_ NSs synthesized by b) ultrasonic probe and c) USMR. d) Size distribution of Ru_1_Co_1_‐1@E‐MoS_2‐x_ NSs at different flow rates. e) The Ru and Co element contents in Ru_1_Co_1_‐1@E‐MoS_2‐x_ NSs samples at different ultrasonic powers tested by the ICP‐MS method. f) Schematic diagram of the ultrasound effect in the USMR pipeline.

S‐vacancy‐rich E‐MoS_2_ NSs loaded with RuCo alloy (RuCo@E‐MoS_2‐x_ NSs) were synthesized using the USMR. In **Figure** **1**a, Ru and Co precursors were initially added to the E‐MoS_2_ NSs aqueous solution and uniformly stirred to adsorb Ru and Co. Subsequently, the mixed solution and NaBH_4_ solution were introduced into USMR via pumps, and the anchoring RuCo alloy on E‐MoS_2_ NSs was rapidly formed under the 20 kHz ultrasound. As shown in Figure S8a,b (Supporting Information), the low intensity of the 10° and 31° characteristic peaks in the XRD spectrum indicates that the MoS_2_ synthesized by the USMR maintains a 1T phase amorphous structure. Meanwhile, XPS results show that a high density of S vacancies is generated on the MoS_2_ surface (Tables S2 and S3, Supporting Information). This highly defective substrate provides abundant anchoring points for alloy particles through the vacancy trapping mechanism, achieving particle size control and spatially confined growth. Compared with traditional ultrasonic probes (Figure 1b), the USMR with high fluidic mixing performance achieves uniform deposition of alloy particles on MoS_2_ (Figure 1c). Finally, a RuCo@E‐MoS_2‐x_ composite catalyst with a small size, uniform distribution, and strong interface interaction is obtained. It is particularly noteworthy that the wide alloy peak at 43° in the XRD spectrum (Figure S8a,b, Supporting Information),^[^ ^10^ ^]^ high‐angle annular dark field scanning transmission electron microscopy (HAADF‐STEM), and the corresponding energy dispersive X‐ray (EDX) element mapping images (Figures S9–S14, Supporting Information) further confirm that the RuCo alloy has a small particle size and is successfully loaded on the E‐MoS_2‐x_ NSs.

In addition, the process scalability of the USMR is also reflected in its ability to precisely control the synthesis conditions. The particle size of the alloy can be controlled by adjusting the ultrasonic power and liquid flow rate. The statistical results of the HAADF‐STEM image (Figure 1d; Figure S15a–d, Supporting Information) show that the size of the RuCo alloy gradually decreases with the increase of the flow rate. This is because at low flow rates, the reducing agent mixes slowly with the aqueous solution of E‐MoS_2_ NSs, resulting in uneven reduction and agglomeration of the RuCo alloy. At high flow rates (20 mL min^−1^), the reactants mix rapidly, forming a large number of nuclei and ultimately producing fine, evenly distributed nanoparticles (Figure 1d). However, high flow rates also shorten the contact and adsorption time between the metal precursor and the support, resulting in a decrease in the overall adsorption capacity. The RuCo alloy exhibited minimal size change under varying ultrasonic powers (Figure S15d–f, Supporting Information). However, at low ultrasonic powers, the RuCo alloy exhibited poor dispersion at the edges of the E‐MoS_2‐x_ NSs and exhibited severe agglomeration (Figure S13, Supporting Information). ICP analysis determined the actual metal loading of RuCo alloy on E‐MoS_2‐x_ NSs at different ultrasonic powers. Figure 1e shows that increasing the ultrasonic power can increase the loading of Ru and Co, because high‐power ultrasound can effectively promote the transfer of mass and heat and shorten the reaction time.^[^ ^38^, ^39^, ^40^, ^41^, ^42^ ^]^ However, when the ultrasonic power increases from 40 W to 60 W, the actual loading of Ru and Co decreases. This is likely due to the negative effects of excessive cavitation. Excessively high ultrasonic power generates excessive cavitation bubble clusters in the liquid. These dense bubble clusters scatter and attenuate acoustic energy, reducing the effective acoustic energy available to promote chemical reactions and mass transfer. Furthermore, strong cavitation causes NaBH_4_ to rapidly release a large amount of H_2_, resulting in a decrease in the content of hydrogen‐reducing species in the solution and insufficient reduction capacity for RuCo alloys.^[^ ^43^, ^44^, ^45^, ^46^, ^47^ ^]^ Therefore, there is an optimal ultrasonic power window (40 W in this study). At this power, the benefits of the cavitation effect (promoting mass transfer, reduction reaction, and dispersion) are maximized. Furthermore, control experiments conducted without ultrasound demonstrated the lowest metal loading rate. This result confirms that the synergistic effect of acoustic cavitation and microchannels in the USMR can significantly improve the mass transfer efficiency. Finally, RuCo alloy particles with a particle size of less than 5 nm were uniformly dispersed on the surface of MoS_2_ NSs (Figure 1f). The LSV curve results indicate that the HER performance is optimal at an impact flow rate of 20 mL min^−1^ and an ultrasonic power of 40 W (Figure S16, Supporting Information). Therefore, subsequent studies were conducted at a collision flow rate of 20 mL min^−1^ and an ultrasonic power of 40 W.

**Figure 2:**
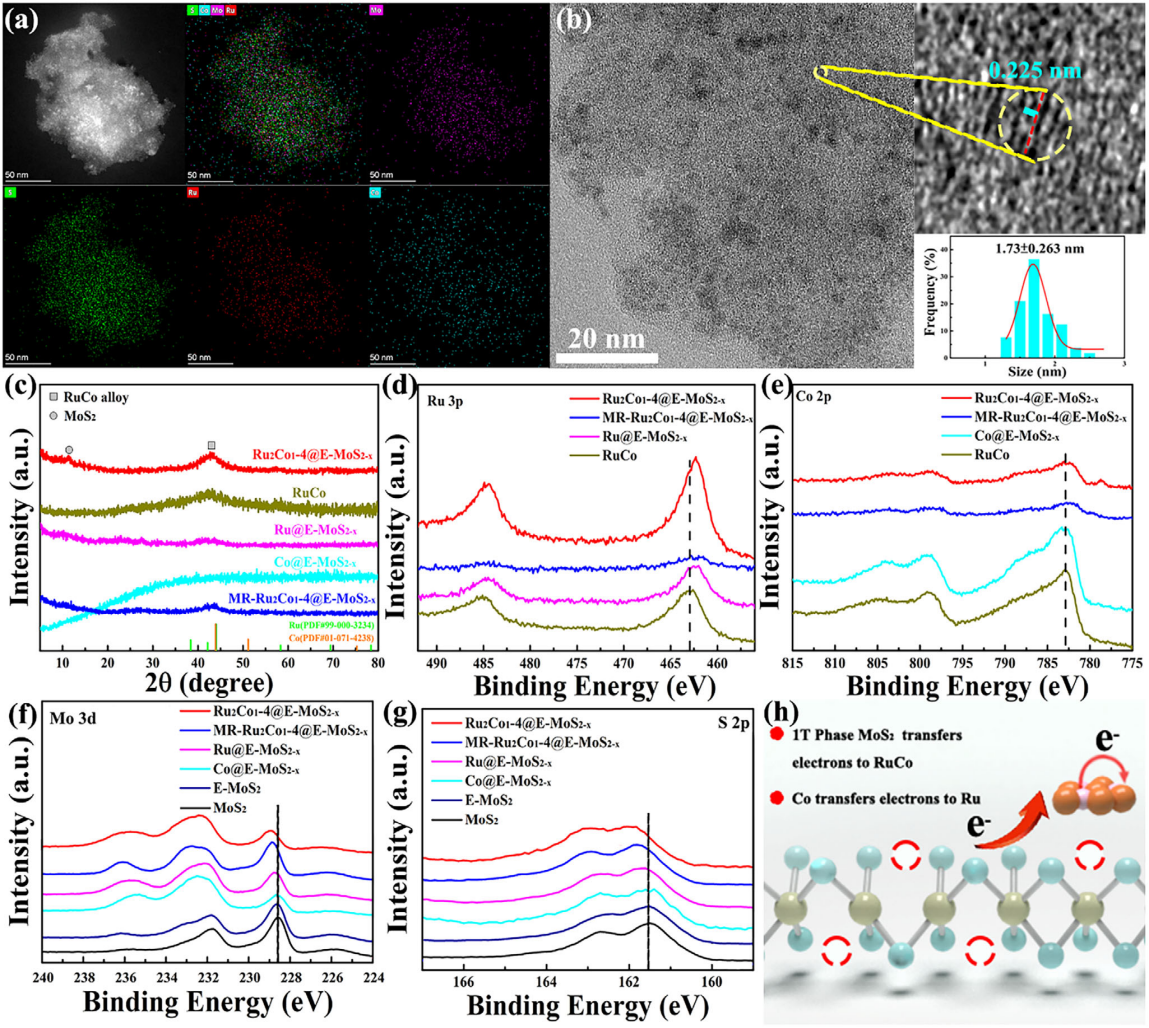
a) EDX elemental mapping image of Ru_2_Co_1_‐4@E‐MoS_2‐x_ NSs. b) HRTEM image of Ru_2_Co_1_‐4@E‐MoS_2‐x_ NSs (in the right, the magnified HRTEM image of the yellow circle and the particle size distribution of RuCo alloy on Ru_2_Co_1_‐4@E‐MoS_2‐x_ NSs). c) XRD patterns of Ru_2_Co_1_‐4@E‐MoS_2‐x_ NSs and control catalysts. d) Ru 3p, e) Co 2p, f) Mo 3d, and g) S 2p high‐resolution XPS spectra of Ru_2_Co_1_‐4@E‐MoS_2‐x_ NSs and control catalysts. h) Charge transfer diagram.

Based on the above experimental methods, different loading amounts and metal ratios of RuCo alloys were explored. TEM, HAADF‐STEM, and corresponding EDX element mapping techniques were used to analyze the representative material Ru_2_Co_1_‐4@E‐MoS_2‐x_ NSs. Monodisperse, small‐sized RuCo alloy nanoparticles can be intuitively observed on Ru_2_Co_1_‐4@E‐MoS_2‐x_ NSs (**Figure** **2**a,b). The average nanoparticle diameter measured 1.74 nm (Figure 2a,b; Figure S27, Supporting Information), with a lattice spacing of 0.225 nm (Figure 2b). It is between 0.204 nm of monometallic Ru (101) grains and 0.244 nm of monometallic Co (111) grains. This belongs to the (100) crystal plane of the RuCo alloy with hcp structure, which conforms to Vegard's law.^[^ ^22^, ^48^ ^]^ The XPS spectra of RuCo alloys with different metal ratios loaded on E‐MoS_2‐x_ NSs show that in Ru_2_Co_1_‐4@E‐MoS_2‐x_ NSs, the 3p orbitals of Ru^0^ and Ru^n+^ shift more toward lower binding energy, and the Co 2p orbital shifts more significantly toward higher binding energy (Figure S23b,c, Supporting Information). This indicates that there is a stronger electronic coupling effect between Ru and adjacent Co in Ru_2_Co_1_‐4@E‐MoS_2‐x_ NSs. Furthermore, the strong electronic interaction between Ru and Co atoms further confirms the formation of the RuCo alloy. This interaction also enhances charge transfer between atoms, thereby enhancing its catalytic activity.^[^ ^22^, ^49^ ^]^ For details about E‐MoS_2‐x_ NSs loaded with RuCo alloys with different loading amounts and metal ratios, please see Note S2 in the supporting information. Here, the core interest is investigating the electrocatalytic HER performance and mechanism of Ru_2_Co_1_‐4@E‐MoS_2‐x_ NSs in acidic and alkaline conditions. Comparative catalysts include Co@E‐MoS_2‐x_ NSs, Ru@E‐MoS_2‐x_ NSs, RuCo alloy, 20 wt% Pt/C, and Ru_2_Co_1_‐4@E‐MoS_2‐x_ NSs prepared in a microreactor (MR‐Ru_2_Co_1_‐4@E‐MoS_2‐x_ NSs. XRD spectra, XPS patterns, HAADF‐STEM images, and corresponding EDX elemental mapping images (Figure 2c–g; Figures S25–S28, Supporting Information) confirm the successful preparation of these samples. The XPS spectra (Figure 2f,g) show that the Mo 3d orbitals and S 2p orbitals of Ru_2_Co_1_‐4@E‐MoS_2‐x_ NSs shift to higher binding energies compared with MoS_2_ nanoflowers and E‐MoS_2‐x_ nanosheets. Meanwhile, it can be observed that the Ru 3p and Co 2p orbitals of Ru_2_Co_1_‐4@E‐MoS_2‐x_ NSs shifted to lower binding energy compared to the RuCo alloy. These results indicate that electrons are transferred from E‐MoS_2‐x_ NSs to the alloy, suggesting a strong interaction between the alloy and the E‐MoS_2‐x_ NSs interface.^[^ ^50^ ^]^ Figure 2h is a schematic diagram of charge transfer in Ru_2_Co_1_‐4@MoS_2‐x_.

**Figure 3:**
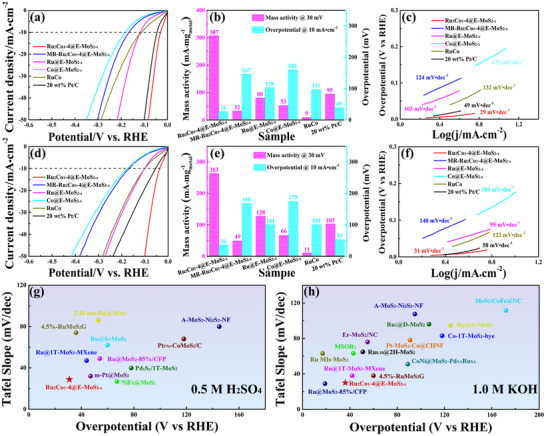
LSV curves of Ru_2_Co_1_‐4@E‐MoS_2‐x_ NSs and control catalysts in a) 0.5 M H_2_SO_4_ and d) 1.0 M KOH. b) The mass activity and overpotential (at 10 mA·cm^−1^) of Ru_2_Co_1_‐4@E‐MoS_2‐x_ NSs and control catalysts in (b) H_2_SO_4_ and e) 1.0 M KOH. Tafel plots of Ru_2_Co_1_‐4@E‐MoS_2‐x_ NSs and control catalysts in c) 0.5 M H_2_SO_4_ and f) 1.0 M KOH. Comparison with the recently reported MoS_2_‐supported HER catalysts in g) 0.5 M H_2_SO_4_ and h) 1.0 M KOH.

Subsequently, the HER activity of these samples was comprehensively evaluated in acidic and alkaline electrolytes. From the LSV curves and histograms in **Figure** **3**a,b, it is evident that in 0.5 M H_2_SO_4_ solution, Ru_2_Co_1_‐4@E‐MoS_2‐x_ NSs exhibit an overpotential of 31 mV (𝜂 = 10 mA·cm^−2^), significantly lower than MR‐Ru_2_Co_1_‐4@E‐MoS_2‐x_ NSs (167 mV), Co@E‐MoS_2‐x_ NSs (183 mV), Ru@E‐MoS_2‐x_ NSs (118 mV), RuCo (111 mV), and 20 wt% Pt/C (45 mV). Benefiting from the uniform distribution of small‐sized RuCo alloy on E‐MoS_2‐x_ NSs, Ru_2_Co_1_‐4@E‐MoS_2‐x_ NSs exhibit a high mass activity of 307 mA·mg_metal_^−1^ at an overpotential of 30 mV, surpassing MR‐Ru_2_Co_1_‐4@E‐MoS_2‐x_ NSs, Co@E‐MoS_2‐x_ NSs, Ru@E‐MoS_2‐x_ NSs, RuCo alloy, and 20 wt% Pt/C by factors of 9.6, 5.8, 3.8, 34.1, and 3.2, respectively (Figure 3b; Figure S33, Supporting Information). Furthermore, Ru_2_Co_1_‐4@E‐MoS_2‐x_ NSs also exhibit the smallest Tafel slope and R_ct_ (Figure 3c; Figure S34, Supporting Information). Further extrapolating the Tafel plot to η = 0 mV, the exchange current density (j_0_) of Ru_2_Co_1_‐4@E‐MoS_2‐x_ NSs is calculated to be 0.20 mA·cm^−2^, which is higher than that of other samples, indicating that it has faster reaction kinetics and follows a more efficient Tafel mechanism for HER. To comprehensively assess the HER performance of Ru_2_Co_1_‐4@E‐MoS_2‐x_ NSs, overpotential at higher current densities was analyzed (Figure S35, Supporting Information). Results indicate that at 𝜂>200 mA·cm^−2^, Ru_2_Co_1_‐4@E‐MoS_2‐x_ NSs demonstrate superior HER activity compared to 20 wt% Pt/C. Additionally, the HER activity of Ru_2_Co_1_‐4@E‐MoS_2‐x_ NSs was further investigated in alkaline electrolytes. Figure 3d depicts the LSV curves of Ru_2_Co_1_‐4@E‐MoS_2‐x_ NSs and other comparative catalysts in a 1.0 M KOH solution. The results show that Ru_2_Co_1_‐4@E‐MoS_2‐x_ NSs still have excellent HER activity. Ru_2_Co_1_‐4@E‐MoS_2‐x_ NSs exhibit a high mass activity of 263 mA·mg_metal_^−1^ at an overpotential of 30 mV (Figure 3e; Figure S36, Supporting Information). This represents 2.55 times the mass activity of 20 wt% Pt/C, indicating high atomic utilization. Additionally, the Tafel slope and R_ct_ of Ru_2_Co_1_‐4@E‐MoS_2‐x_ NSs are lower than those of 20 wt% Pt/C (Figure 3f; Figure S37, Supporting Information), indicative of enhanced H_2_O dissociation and faster reaction kinetics. Notably, the EIS frequency range for Ru_2_Co_1_‐4@E‐MoS_2‐x_ was extended to 100 kHz–0.01 Hz (Figure S38, Supporting Information). No significant Warburg impedance was observed in the low‐frequency region (0.1 Hz–0.01 Hz). This reveals that, under the potential and system studied here, the hydrogen evolution reaction is primarily controlled by the charge transfer step, while the adsorption/desorption of hydrogen atoms or their diffusion within the bulk phase of the material are not the rate‐determining steps of the entire reaction. This is due to the extremely high intrinsic catalytic activity of the electrode material we designed, which results in a very fast hydrogen adsorption/desorption step. More importantly, Ru_2_Co_1_‐4@E‐MoS_2‐x_ exhibits competitive performance with recently reported MoS_2_‐supported catalysts in both acidic and alkaline media (Figure 3g,h; Tables S6 and S7, Supporting Information). Notably, the HER activity of Ru_2_Co_1_‐4@E‐MoS_2‐x_ NSs in acidic electrolytes is slightly higher than in alkaline electrolytes. This is primarily because, in acidic conditions, a greater concentration of H^+^ facilitates the Volmer step, whereas, in alkaline conditions, H_2_O must dissociate to produce more accessible H^+^ intermediates. This is also the reason for its slow reaction kinetics.^[^ ^51^ ^]^

**Figure 4:**
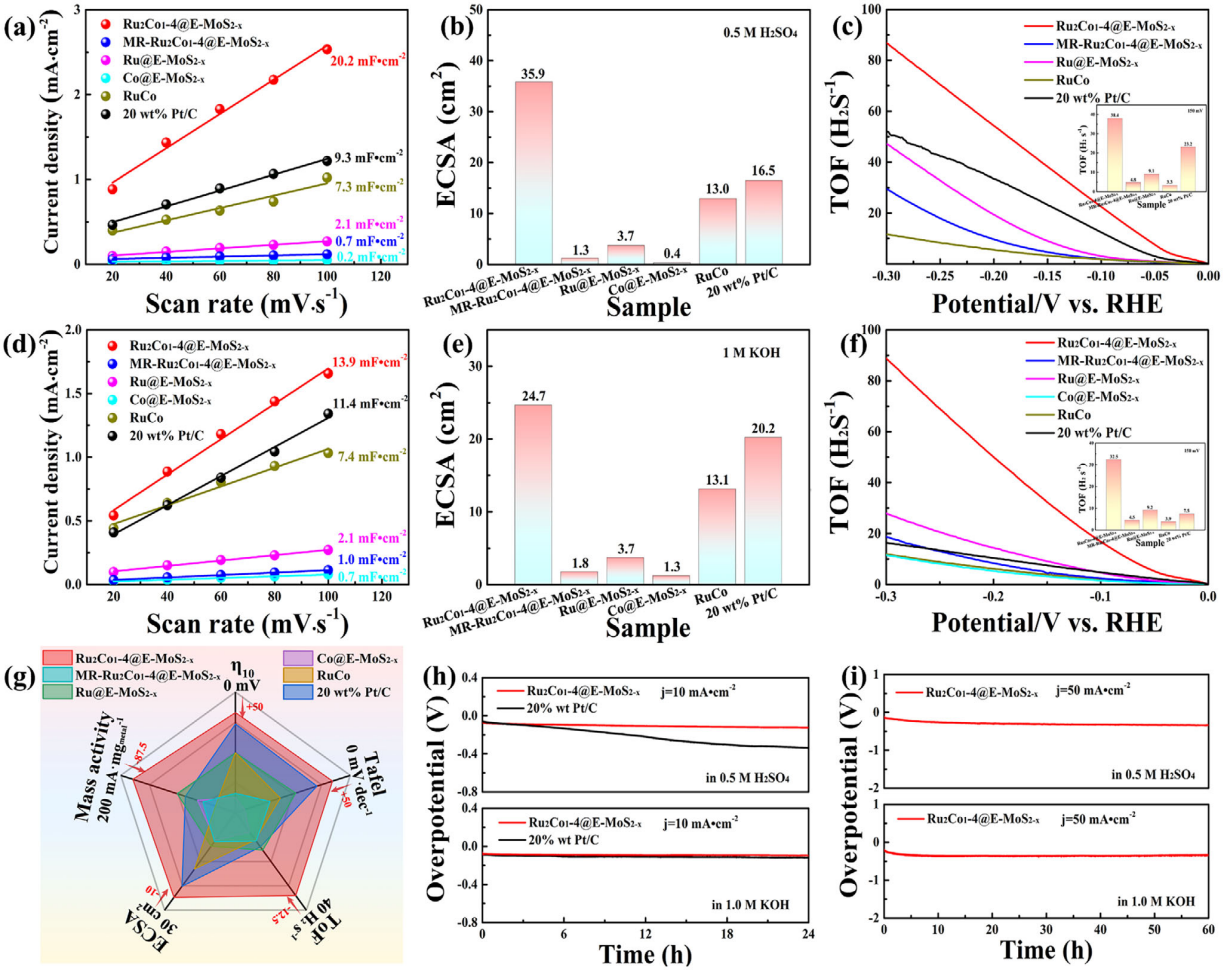
C_dl_ values of Ru_2_Co_1_‐4@E‐MoS_2‐x_ NSs and control catalysts in a) 0.5 M H_2_SO_4_ and d) 1.0 M KOH. ECSA values of Ru_2_Co_1_‐4@E‐MoS_2‐x_ NSs and control catalysts in b) 0.5 M H_2_SO_4_ and e) 1.0 M KOH. TOF plots and TOF value of Ru_2_Co_1_‐4@E‐MoS_2‐x_ NSs and control catalysts at an overpotential of 150 mV in c) 0.5 M H_2_SO_4_ and f) 1.0 M KOH. g) Comparison of 𝜂_10_, ECSA, Tafel slope, TOF and mass activity of different catalysts in 1.0 M KOH. h) Chronopotentiometry curves of Ru_2_Co_1_‐4@E‐MoS_2‐x_ NSs, 20 wt% Pt/C at 10 mA·cm^−2^ in 0.5 M H_2_SO_4_ and 1.0 M KOH. i) Chronopotentiometry curves of Ru_2_Co_1_‐4@E‐MoS_2‐x_ NSs, 20 wt% Pt/C at 50 mA·cm^−2^ in 0.5 M H_2_SO_4_ and 1.0 M KOH.

To better understand the intrinsic catalytic activity of Ru_2_Co_1_‐4@E‐MoS_2‐x_ NSs, ECSA values and turnover frequency (TOF) were measured. **Figure** **4**a,b,d,e and Figures S39 and S40 (Supporting Information) show the CV curves and corresponding ECSA values of Ru_2_Co_1_‐4@E‐MoS_2‐x_ NSs, MR‐Ru_2_Co_1_‐4@E‐MoS_2‐x_ NSs, Ru@E‐MoS_2‐x_ NSs, Co@E‐MoS_2‐x_ NSs, RuCo alloy, and 20 wt% Pt/C in acidic and alkaline conditions. Among them, Ru_2_Co_1_‐4@E‐MoS_2‐x_ NSs exhibit the highest ECSA values of 35.9 cm^2^ under acidic conditions and 24.7 cm^2^ under alkaline conditions. Significantly, the active surface area of Ru_2_Co_1_‐4@E‐MoS_2‐x_ NSs is much larger than that of Ru@E‐MoS_2‐x_ NSs and Co@E‐MoS_2‐x_ NSs. This occurs because the introduction of Co atoms reduces the size. Simultaneously, the synergistic effect between Ru and Co atoms exposes new active sites, significantly increasing the number of active sites of the catalyst.^[^ ^48^ ^]^ TOF values were further calculated using Ru atoms as the active sites, as depicted in Figure 4c,f. Under acidic and alkaline conditions, Ru_2_Co_1_‐4@E‐MoS_2‐x_ NSs achieved maximum TOF values of 38.4 H_2_·s^−1^ and 32.5 H_2_·s^−1^ at 150 mV, respectively, outperforming 20 wt% Pt/C and other comparative catalysts. In summary, from the perspective of overpotential, Tafel slope, j_0_, TOF, ECSA, and mass activity, Ru_2_Co_1_‐4@E‐MoS_2‐x_ NSs outperform all these samples in HER performance in both alkaline and acidic media (Figure 4g; Figure S41, Supporting Information, **Table** **1** and Figure S8, Supporting Information). These analyses further confirm that Ru_2_Co_1_‐4@E‐MoS_2‐x_ NSs have high HER activity under both acidic and alkaline conditions and have excellent H_2_ evolution ability.

**Table 1:** Comparison of 𝜂_10_, Tafel slope, j_0_, ECSA, mass activity, and TOF of different catalysts in 1.0 M KOH.

	η_10_ [mv]	Tafel slope [mV·dec^−1^]	j_0_ [mA·cm^−2^]	ESCA [cm^2^]	Mass activity @ 30 mV [mA·mg_metal_^−1^]	TOF @ 150 mV [H_2_ s^−1^]
Ru_2_Co_1_‐4@E‐MoS_2‐x_	36	31	1.90	24.7	263	32.5
MR‐Ru_2_Co_1_‐4@E‐MoS_2‐x_	168	140	0.78	1.8	49	4.5
Ru@E‐MoS_2‐x_	101	95	1.10	3.7	128	9.2
Co@E‐MoS_2‐x_	175	184	1.17	1.3	66	‐
RuCo	101	122	1.69	13.1	11	3.9
20 wt% Pt/C	53	58	1.90	20.2	103	7.5

Based on the results of the LSV curve, Tafel slope, and CV curve, there was a significant disparity in the HER activity between MR‐Ru_2_Co_1_‐4@E‐MoS_2‐x_ NSs and Ru_2_Co_1_‐4@E‐MoS_2‐x_ NSs. XPS results (Table S9 and Figure S42, Supporting Information) indicated that MR‐Ru_2_Co_1_‐4@E‐MoS_2‐x_ NSs had lower Ru content than Ru_2_Co_1_‐4@E‐MoS_2‐x_ NSs. This result suggests that the reduction efficiency of Ru using NaBH_4_ in the Microreactor (MR) was insufficient in this experiment. In MR‐Ru_2_Co_1_‐4@E‐MoS_2‐x_ NSs, the actual Ru to Co ratio (0.45:1) is significantly lower than the theoretical value (2:1). This is also the main reason for the low catalytic activity of MR‐Ru_2_Co_1_‐4@E‐MoS_2‐x_ NSs. Due to the ultrasound cavitation effect, cavitation bubbles will generate local high temperatures and high pressures now of collapse, enhancing the mass transfer effect. This promotes the rapid mixing of the NaBH_4_ solution and metal ions, facilitating the reduction reaction.^[^ ^48^ ^]^ Thus, the actual Ru to Co ratio (1.6:1) in Ru_2_Co_1_‐4@E‐MoS_2‐x_ NSs prepared using the USMR closely approximates the theoretical value (2:1). In addition, the XPS results exhibited that the S vacancy concentration of MR‐Ru_2_Co_1_‐4@E‐MoS_2‐x_ NSs was 21.9%, which was higher than 15.6% of Ru_2_Co_1_‐4@E‐MoS_2‐x_ NSs. This may be because when ultrasound is applied, more NaBH_4_ participates in the reduction of Ru. When ultrasound is not used, the reduction ability of NaBH_4_ on Ru is limited, and more NaBH_4_ reacts with MoS_2_ to generate H_2_S, resulting in an increased number of S vacancies. According to previous reports, as the concentration of S vacancies increases, ΔG_H*_ gradually decreases. When the concentration of S vacancies is between 9‐19%, ΔG_H*_ is between ± 0.08 eV. When the concentration of S vacancies increases further, MoS_2_ has a more negative ΔG_H*_. Therefore, the HER activity of Ru_2_Co_1_‐4@E‐MoS_2‐x_ NSs is much better than that of MR‐Ru_2_Co_1_‐4@E‐MoS_2‐x_ NSs. Moreover, the HER activity of Ru_2_Co_1_‐4@E‐MoS_2‐x_ NSs is also significantly lower than that of RuCo alloy (Figure 3a,d). This arises from the interfacial interaction between RuCo alloy and E‐MoS_2‐x_ NSs, optimizing the electronic structure and H adsorption‐free energy of RuCo alloy, increasing the number of active sites, and intrinsic activity. In addition, previous studies have shown that the interaction of OH^*^ on 1T phase MoS_2_ is stronger than with Ru.^[^ ^7^, ^52^ ^]^ Thus, the OH^*^ generated by water dissociation at Ru active sites tends to migrate from Ru sites to the adjacent 1T phase MoS_2_ substrate. This effectively prevents Ru site poisoning and enhances HER activity in an alkaline environment by releasing Ru active sites.

Additionally, the long‐term stability of Ru_2_Co_1_‐4@E‐MoS_2‐x_ NSs is crucial for the practical application and was further assessed using LSV and chronopotentiometry. Figure 4h and Figure S43 (Supporting Information) demonstrate that at a constant current density of 10 mA·cm^−2^, Ru_2_Co_1_‐4@E‐MoS_2‐x_ NSs maintained stable operation in 0.5 M H_2_SO_4_ and 1.0 M KOH for 24 hours without significant deactivation. The LSV curve after the long‐term stability test reveals that the catalytic performance of Ru_2_Co_1_‐4@E‐MoS_2‐x_ NSs at 10 mA·cm^−2^ is slightly attenuated. After the long‐term stability test, the TEM image demonstrates that the RuCo alloy in Ru_2_Co_1_‐4@E‐MoS_2‐x_ NSs has maintained uniform distribution without agglomeration (Figure S44, Supporting Information). XPS analysis was performed on samples after the stability test. The results (Figure S45, Supporting Information) show no significant changes in the valence states of Ru and Co, indicating that the alloy structure remained unchanged during the reaction. Furthermore, in the XRD results (Figure S46, Supporting Information), the diffraction peaks attributed to the RuCo alloy remained clearly visible and showed no significant shift. No diffraction peaks attributable to Ru and Co oxides were detected in the spectrum. This indicates that the RuCo alloy nanostructure maintained its metallic state under the electrochemical test conditions and did not undergo significant oxidation. Furthermore, a comparison of the XRD patterns before and after the stability test revealed no significant changes in the position and shape of the characteristic diffraction peaks attributed to MoS_2_. This indicates that the MoS_2_ crystal structure remained stable during the reaction, with no large‐scale phase transformations detectable by XRD. These results indicate excellent structural stability of Ru_2_Co_1_‐4@E‐MoS_2‐x_ NSs during the HER process. Long‐term stability tests were further conducted at higher current densities. As shown in Figure S47a,b (Supporting Information), the overpotentials required for Ru_2_Co_1_‐4@E‐MoS_2‐x_ to reach a current density of 1 A·cm^−2^ in 0.5 M H_2_SO_4_ and 1.0 M KOH were approximately 430 mV and 910 mV, respectively. Figure 4i and Figure S47c (Supporting Information) show that no obvious overpotential fluctuations were observed after continuous operation at a current density of 50 mA·cm^−2^ and 1 A·cm^−2^ for 60 hours. This demonstrates that the Ru_2_Co_1_‐4@E‐MoS_2‐x_ nanostructure has strong stability and great potential in practical applications.

**Figure 5:**
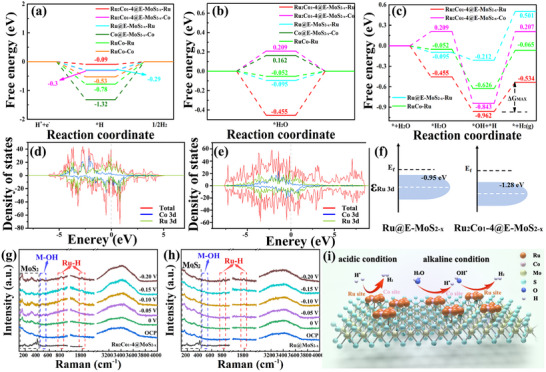
a) ΔG_H*_ of active sites of different samples. b) Adsorption energy diagram of H_2_O at the active sites of different samples. c) Energy diagrams of H_2_O dissociation and H_2_ generation at active sites of different samples. d, e) Density of states (DOS) of Ru@E‐MoS_2‐x_ NSs and Ru_2_Co_1_‐4@E‐MoS_2‐x_ NSs; f) d‐band centers of Ru 3d orbitals for different samples. In situ Raman spectra of HER for g) Ru_2_Co_1_‐4@E‐MoS_2‐x_ NSs and h) Ru@E‐MoS_2‐x_ NSs in 1.0 M KOH; i) Mechanism analysis.

DFT calculations were used to analyze the source of the excellent electrolytic water‐splitting activity of Ru_2_Co_1_‐4@E‐MoS_2‐x_ NSs. The catalyst model used is shown in Figure S48 (Supporting Information), and other calculation parameters and information are described in detail in the supporting information. Taking acidic HER as an example, **Figure** **5**a shows the ΔG_H*_ intermediates at different catalyst sites. Compared with the Ru site of Ru@E‐MoS_2‐x_ NSs, the proton adsorption energy of the Ru site of Ru_2_Co_1_‐4@E‐MoS_2‐x_ NSs weakens from −0.29 eV to −0.09 eV, which is closer to the ideal value of 0 eV. XPS results (Figure 2d,e) reveal that the Ru 3p and Co 2p core energy levels in Ru_2_Co_1_‐4@E‐MoS_2‐x_ NSs shift toward lower binding energies compared to the control catalysts. This is due to the excellent electrical conductivity of E‐MoS_2‐x_. When RuCo alloy nanoparticles come into contact with this defect‐rich MoS_2_ surface, a strong interaction forms. Electrons flow significantly from the MoS_2_ support to the RuCo alloy nanoparticles. Therefore, compared to monometallic Ru or Co, the RuCo alloy forms a more stable interface with the MoS_2_ support, more effectively facilitating electron transfer from the support to the alloy. Charge delocalization within the alloy, achieved through d‐d orbital hybridization, allows both Ru and Co to share the extra electrons from the support, ultimately reducing the binding energy of both. The density of states (DOS) of Ru sites on different catalysts was studied. As shown in Figure 5d,e, the d‐band center of Ru in the Ru_2_Co_1_‐4@E‐MoS_2‐x_ NSs model is lower than that of Ru@E‐MoS_2‐x_ NSs and RuCo alloy (Figure 5f), which helps to reduce ΔG_H*_. This is consistent with the results of XPS. The alloying of Ru and Co causes the Ru's d‐band center to shift downward, weakening the metal‐adsorbate interaction and thus optimizing the catalytic activity of the Ru site.^[^ ^53^, ^54^ ^]^ At the same time, the introduction of Co further optimizes the electronic state of Ru through d‐d orbital hybridization. This electronic reconstruction puts the Ru site in a partially reduced state (∼Ru^δ−^), significantly improving its polarization ability for water molecules and facilitating the migration of ions.^[^ ^55^ ^]^ The above results show that the introduction of Co can effectively balance the kinetics of the H^*^ adsorption and desorption process at the Ru site, making the Ru site an efficient reaction site. In addition, the adsorption energy of the Co site in Ru_2_Co_1_‐4@E‐MoS_2‐x_ NSs has also been greatly optimized. The adsorption energy of the Co site in Ru_2_Co_1_‐4@E‐MoS_2‐x_ NSs (−0.3 eV) is much lower than that of Co@E‐MoS_2‐x_ NSs (−1.32 eV). This is due to the alloying effect of Co and Ru, which weakens the strength of the Co─H bond.^[^ ^12^ ^]^ The above results show that Ru_2_Co_1_‐4@E‐MoS_2‐x_ NSs have a bimetallic active site. This makes the proton adsorption present a complementary gradient between the Ru site and the Co site, with the Ru site as the main active center to achieve rapid proton adsorption/desorption (ΔG_H*_≈0), and the Co site stabilizing the reaction intermediates through moderate adsorption. This complementary adsorption gradient helps to better balance the capture of protons and the desorption of hydrogen. In addition, the Ru and Co sites' adsorption energies in the RuCo alloy (−0.78 and −0.53 eV, respectively) are higher than those of Ru_2_Co_1_‐4@E‐MoS_2‐x_ NSs. This may be attributed to the interaction between E‐MoS_2‐x_ NSs and RuCo alloys, which prompts the rearrangement of the electron density on the surface of the RuCo alloy. The results of XPS show (Figure 2g,h) that the core energy levels of Mo 3d and S 2p of Ru_2_Co_1_‐4@E‐MoS_2‐x_ NSs move toward the high binding energy direction compared with the E‐MoS_2‐x_ NSs. This indicates a strong interface coupling between E‐MoS_2‐x_ NSs and RuCo alloys. At the same time, the high conductivity of the 1T phase MoS_2_ accelerates the electron transfer from the MoS_2_ carrier to the RuCo alloy. Therefore, the synergistic effect of 1T phase MoS_2_ with S vacancies and RuCo alloy can effectively reduce the adsorption energy of the alloy for protons. All the above results demonstrate that the bimetallic active site Ru_2_Co_1_‐4@E‐MoS_2‐x_ NSs model exhibits an ideal proton adsorption strength, resulting in faster HER kinetics and enhanced catalytic activity.

In alkaline media, H^*^ comes from the dissociation of H_2_O molecules (Figures S49 and S50, Supporting Information). Therefore, the adsorption, dissociation of H_2_O molecules, and the desorption of H_2_ molecules are of great significance for the overall HER activity. As shown in Figure 5b, the ΔG_H2O*_ of the Co site on Ru_2_Co_1_‐4@E‐MoS_2‐x_ NSs and RuCo alloys is positive, and it is difficult to spontaneously adsorb H_2_O molecules. The Ru site of Ru_2_Co_1_‐4@E‐MoS_2‐x_ NSs exhibits the strongest water adsorption ability and can effectively capture H_2_O molecules. It is known that H^*^ and ^*^OH intermediates co‐adsorb on the surface of the electrocatalyst during H_2_O dissociation. Therefore, the co‐adsorption energy of H^*^ and ^*^OH intermediates (E_(H*+*OH)_) on the catalyst reflects its capacity to provide available H^*^ intermediates.^[^ ^56^ ^]^ As shown in Figure 5c, the Ru and Co sites on Ru_2_Co_1_‐4@E‐MoS_2‐x_ NSs exhibit more negative E_(H*+*OH)_ values (‐0.962 and ‐0.843 eV, respectively) than other catalyst sites. This indicates that alloying Ru with Co can significantly enhance the adsorption of H^*^ and ^*^OH intermediates on Ru and Co sites. Consequently, Ru_2_Co_1_‐4@E‐MoS_2‐x_ NSs are more suitable for operation under alkaline conditions with strong ^*^OH connections. The rate‐determining step of the HER reaction on the electrocatalyst is the generation and release of H_2_ based on the maximum Gibbs free energy change (ΔG_max_).^[^ ^57^ ^]^ The ΔG_max_ of the Ru sites on RuCo and Ru@E‐MoS_2‐x_ NSs is higher than that of Ru_2_Co_1_‐4@E‐MoS_2‐x_ NSs (Figure 5c), indicating that the strong binding of H makes it difficult for H_2_ to desorb, resulting in poor performance. Therefore, in alkaline media, the synergistic effect of RuCo bimetallic sites and MoS_2_ balances the energy relationship of adsorption‐activation‐desorption, making it exhibit better HER performance.

The results of in‐situ Raman spectroscopy (Figure 5g,h; Figure S51, Supporting Information) show that at a starting potential of 50 mV, the Ru@MoS_2‐x_ system exhibits strong hydrogen adsorption capacity (ΔG_H*_ = −0.3 eV) due to the isolated Ru sites, resulting in significant accumulation of H intermediates on its surface and showing an obvious Ru‐H characteristic peak.^[^ ^58^ ^]^ On the contrary, no Ru‐H peak was detected in Ru_2_Co_1_‐4@MoS_2‐x_ at the same potential, confirming that Co alloying reduces the hydrogen adsorption free energy of Ru sites (ΔG_H*_ = −0.09 eV) through electronic structure regulation (downward shift of the d‐band center), prompting H intermediates to quickly participate in the desorption reaction after formation, avoiding excessive aggregation. At the same time, in the 500 cm^−1^ region, Ru_2_Co_1_‐4@MoS_2‐x_ exhibits the strongest M‐OH vibration peak intensity.^[^ ^59^ ^]^ This is attributed to the synergistic adsorption effect of the bimetallic sites on OH intermediates. The Co site efficiently captures OH intermediates with its optimized electronic structure, effectively avoiding the occupation of OH at the Ru active site, thereby releasing the Ru site to continuously split new water molecules, significantly improving the water dissociation rate. In addition, the stable adsorption of OH by the Co site reduces the concentration of free OH^−^ at the interface, which helps to maintain the structural integrity of the MoS_2_ carrier, which is specifically reflected in the stable MoS_2_ intrinsic peaks in the range of 100∼410 cm^−1^. Further analysis of the potential dependence shows that: in the range of −0.10 V to −0.20 V, the Ru‐H peak intensity of Ru_2_Co_1_‐4@MoS_2‐x_ is relatively constant, indicating that H reaches a balance between dynamic accumulation and desorption; while the Ru‐H peak intensity of Ru@MoS_2‐x_ continues to increase, indicating that high ΔG_H*_ leads to excessive aggregation of H, resulting in active site blockage. In contrast, the introduction of Co reduces the adsorption intensity of H on the Ru site and promotes the desorption kinetics, which is consistent with the theoretical calculation results. It is worth noting that the Co@MoS_2‐x_ sample only shows a weak Co‐OH peak at ‐0.20 V and no Co‐H signal, which supports the limited water dissociation ability of the Co site alone and the weak adsorption capacity for OH and H. The broad Raman peak in the range of 3200 to 3600 cm^−1^ can be attributed to the O‐H stretching vibration mode of interfacial water. The interfacial water peak can be analyzed into three components by peak fitting, located at 3235 cm^−1^, 3445 cm^−1^, and 3610 cm^−1^, corresponding to tetrahedral coordinated water, trihedral coordinated water, and free water.^[^ ^58^, ^60^ ^]^ Among them, free water exists in the H‐down configuration, indicating that it is easy to adsorb on the catalyst surface. In addition, the activation energy barrier of free water is lower than that of tetrahedral coordinated water and trihedral coordinated water. Therefore, the higher the proportion of free water in the sample, the lower the energy required to dissociate the water molecule.^[^ ^61^ ^]^ The data in Figure S43b (Supporting Information) show that at a potential of ‐0.20 V, the proportion of free water in Ru_2_Co_1_‐4@E‐MoS_2‐x_ NSs (10.1%) is higher than that in Co@E‐MoS_2‐x_ NSs (8.1%) and Ru@E‐MoS_2‐x_ NSs (8.9%). This indicates that Ru_2_Co_1_‐4@E‐MoS_2‐x_ NSs is more likely to destroy the hydrogen bond network structure of tetrahedral coordinated water and trihedral coordinated water, thereby generating more free water, which reveals the intrinsic correlation of its excellent catalytic activity in alkaline media. Figure 5i shows the mechanism diagram of bimetallic active site Ru_2_Co_1_‐4@E‐MoS_2‐x_ NSs for water decomposition over a wide pH range. Ru sites are the primary active centers for hydrogen adsorption/desorption. Their electronic structure is optimized by Co alloying, resulting in a near‐thermoneutral ΔG_H*_, enabling a fast Volmer‐Tafel step. Co‐sites serve multiple functions as cocatalysts. First, the introduction of Co modulates the electronic structure of Ru, reducing its ΔG_H*_. Second, in alkaline environments, they act as oxygen‐loving sites, strongly adsorbing OH, promoting water dissociation, and preventing OH^*^ from poisoning the Ru sites. The high conductivity of E‐MoS_2‐x_ as a support and its strong interfacial interaction with the alloy enable fast electron transport. Furthermore, S vacancies act as anchors to stabilize the metal nanoparticles and provide edge active sites.

**Figure 6:**
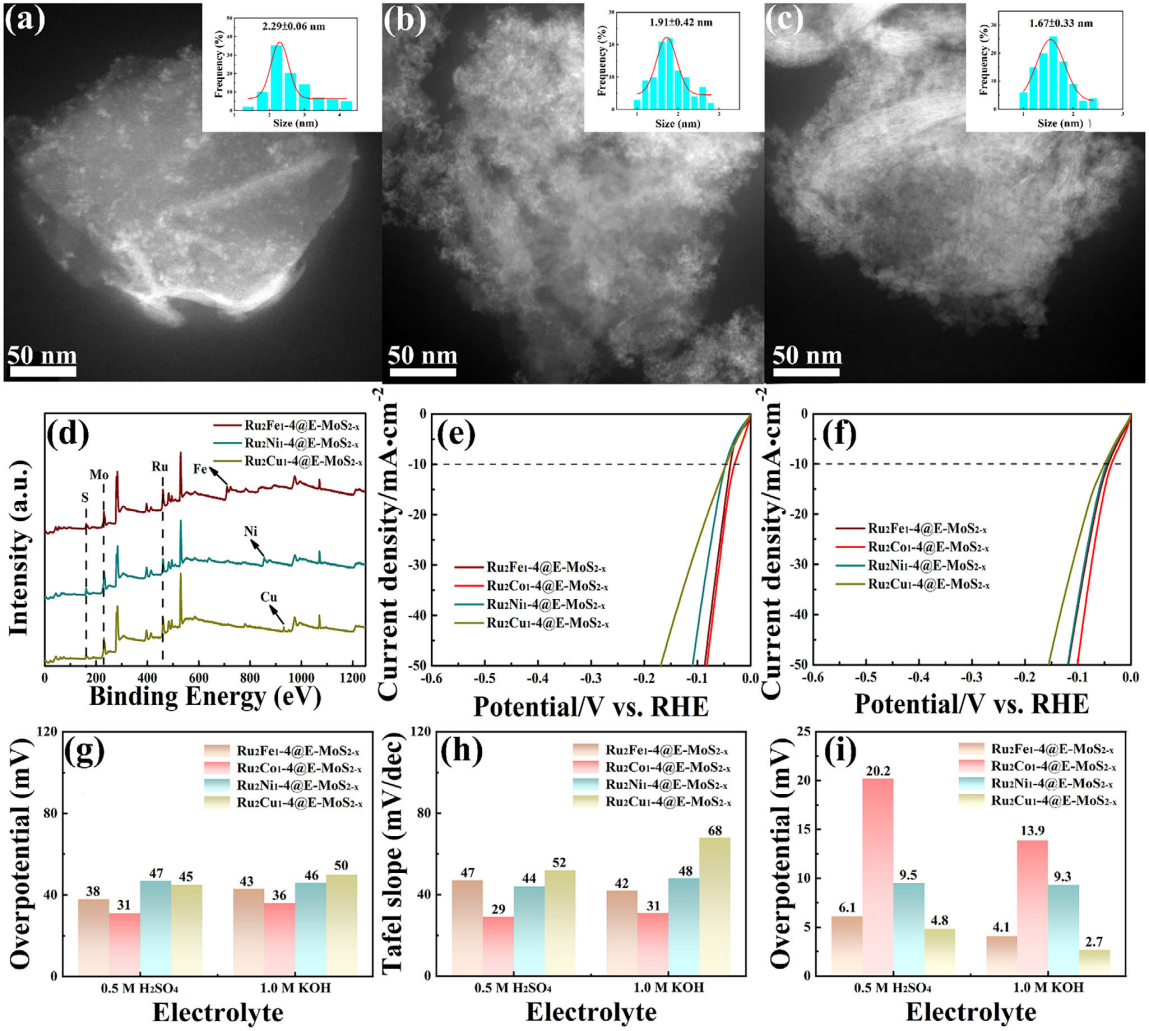
TEM images and Size distribution of the a) Ru_2_Fe_1_‐4@E‐MoS_2‐x_ NSs, b) Ru_2_Ni_1_‐4@E‐MoS_2‐x_ NSs, and c) Ru_2_Cu_1_‐4@E‐MoS_2‐x_ NSs. d) XPS spectra of Ru_2_Fe_1_‐4@E‐MoS_2‐x_ NSs, Ru_2_Ni_1_‐4@E‐MoS_2‐x_ NSs, and Ru_2_Cu_1_‐4@E‐MoS_2‐x_ NSs. LSV curves of E‐MoS_2‐x_ NSs loaded with different Ru‐based alloys in e) 0.5 M H_2_SO_4_ and f) 1.0 M KOH. Corresponding g) 𝜂_10_ values, h) Tafel slopes, and i) C_dl_ values of E‐MoS_2‐x_ NSs loaded with different Ru‐based alloys in different electrolytes.

The strategy of preparing Ru_2_Co_1_ alloy loaded on E‐MoS_2‐x_ NSs using USMR can be further extended to Ru_2_Fe_1_, Ru_2_Ni_1_, and Ru_2_Cu_1_ alloys. It is only necessary to change the cobalt chloride precursor to ferric chloride, nickel chloride, and cupric chloride, respectively. TEM images, XPS spectra, and EDX elemental mapping images (**Figure** **6**a–d; Figures S52–S54, Supporting Information) demonstrate the presence and uniform distribution of Ru, Fe, Ni, and Cu elements on the E‐MoS_2‐x_ NSs. The average diameters of Ru_2_Fe_1_, Ru_2_Ni_1_, and Ru_2_Cu_1_ alloy nanoparticles are 2.3, 1.9, and 1.7 nm, respectively. As expected, the loading of Ru_2_Fe_1_, Ru_2_Ni_1_, and Ru_2_Cu_1_ on E‐MoS_2‐x_ NSs also significantly improved the HER activity of the catalysts in both acidic and alkaline electrolytes (Figure 6e–g). For example, Ru_2_Fe_1_‐4@E‐MoS_2‐x_ NSs, Ru_2_Ni_1_‐4@E‐MoS_2‐x_ NSs, and Ru_2_Cu_1_‐4@E‐MoS_2‐x_ NSs show overpotentials of 38, 47, and 45 mV (𝜂 = 10 mA·cm^−2^) in 0.5 M H_2_SO_4_ solution, respectively (Figure 6e). In addition, Ru_2_Fe_1_‐4@E‐MoS_2‐x_ NSs, Ru_2_Ni_1_‐4@E‐MoS_2‐x_ NSs, and Ru_2_Cu_1_‐4@E‐MoS_2‐x_ NSs also have Tafel slopes and ECSA values comparable to those of 20 wt% Pt/C (Figure 6h,i). Similarly, these values are also like those of 20 wt% Pt/C in 1.0 M KOH solution. It should be noted that the RuFe, RuNi, and RuCu alloys were synthesized under the identical procedure as Ru_2_Co_1_‐4@E‐MoS_2‐x_ by simply replacing the metal precursor. However, the inherently distinct nucleation and growth kinetics of each metal system naturally lead to variations in particle size and dispersion.^[^ ^62^, ^63^, ^64^ ^]^ A systematic optimization of metal ratios, loading amounts, and USMR conditions for each specific alloy will be essential in future studies. In summary, we have achieved, for the first time, the simultaneous preparation of S‐vacancy‐rich MoS_2_ and in‐situ anchoring of an ultrafine bimetallic alloy in a one‐step process using USMR technology. We also established a strong synergistic effect between the bimetallic active site and the interface‐engineered support. This can be extended to various bimetallic systems, including RuFe, RuNi, and RuCu, demonstrating the versatility of the “bimetallic‐interface‐engineered support” collaborative design concept. Thanks to these unique structural advantages, HER activity exceeding the commercial 20 wt% Pt/C was achieved, along with excellent stability.

## 3 Conclusion

In summary, this study successfully developed Ru_2_Co_1_₋4@E‐MoS_2‐x_ NSs, an efficient, stable, and broad pH range HER electrocatalyst, by innovatively constructing a “bimetallic active site‐interface engineering carrier” synergistic catalytic system. The synthesis method based on a USMR combines rapid fluid mixing and the ultrasonic cavitation effect. It not only promotes the etching of S vacancies in the MoS_2_ lattice but also provides a high‐energy field for the transient reduction reaction of NaBH_4_ and thereby achieves an ultrafine alloy with a high Ru proportion. The transient high‐pressure microjets generated by cavitation further drive strong coupling between RuCo nanoparticles and the S‐vacancy‐rich E‐MoS_2_ support, forming interfacial bonds that significantly enhance alloy–support adhesion. This unique micro‐reaction system gives the composite catalyst abundant active site exposure and fast charge transfer channels. In situ Raman spectroscopy and DFT calculations show that the bimetallic catalytic system synergistically optimizes the adsorption/desorption equilibrium. The interaction between Ru and Co significantly reduces the ΔG_H*_ of the Ru and Co bimetallic sites, overcoming the contradiction of “strong adsorption‐weak desorption” or “weak adsorption‐low activity” in the monometallic system, and avoiding the active site poisoning due to excessive H^*^ adsorption. Meanwhile, the S‐vacancy‐rich 1T phase MoS_2_ carrier not only accelerates charge transfer through high conductivity, but its interfacial synergistic effect further optimizes the H_2_O dissociation (alkaline conditions) and H_2_ desorption processes. The introduction of OH^*^‐philic Co sites overcomes the energy barrier of alkaline water dissociation and H^*^ coupling, greatly improving the activity of alkaline HER. Under acidic and alkaline conditions, Ru_2_Co_1_₋4@E‐MoS_2‐x_ NSs exhibit lower overpotential and better stability than the commercially 20 wt% Pt/C catalyst. This synthesis strategy can be extended to other bimetallic systems such as RuFe, RuNi, and RuCu, verifying the versatility of the “bimetal‐interface engineering carrier” collaborative design concept. The USMR technique reliably produces catalysts with high dispersion, ultrafine particle size, and high metal loading, all of which are critical for superior catalytic performance. This makes the method readily adaptable to a wide range of material systems, including various 2D supports (e.g., graphene, MXene) and porous frameworks (e.g., MOFs, COFs), as well as numerous metal nanoparticles (noble, non‐noble, and alloys) amenable to liquid‐phase reduction. Therefore, USMR represents a versatile and industrially viable platform for the rational design and construction of highly active catalysts.

## Conflict of Interest

The authors declare no conflict of interest.

## Supporting information



Supporting Information

## Data Availability

The data that support the findings of this study are available from the corresponding author upon reasonable request.
