# Supplementary material for: Ultrafine Ru‐M Alloy@S Vacancy‐Rich MoS2 Nanosheets With Bimetallic Active Sites for Efficient Hydrogen Evolution in Alkaline and Acidic Media
Source: Adv Sci (Weinh). 2025 Nov 28;13(8):e19323. doi: 10.1002/advs.202519323 (PMC12884741; doi:10.1002/advs.202519323)
Supplement: Supplementary file 1 — Supporting Information [file ADVS-13-e19323-s001.docx]

**Supporting Information**

**Ultrafine Ru-M alloy@S vacancy-rich MoS₂ nanosheets with bimetallic active sites for efficient hydrogen evolution in alkaline and acidic media**

Rui Su^a, b^, Tiantian Ding^c^, Hao Tang^b, c^, Xueying Yang^d^, Jie Zhang^b^, Xiaolin Liu^c^, Jinfu Jia^b^, Yuan Liu^b,^ *, Jin Ge^a^, Zhilin Wu^b, c^, Zhengya Dong^b, c, *^, Xiaojing Zhu^b, *^

a Laboratory of Bioinorganic and Synthetic Chemistry, GBRCE for Functional Molecular Engineering, LIFM, IGCME, School of Chemistry, Sun Yat-Sen University, Guangzhou 510006, China.

b Chemistry and Chemical Engineering Guangdong Laboratory, 515031 Shantou, China

c College of Chemistry and Chemical Engineering, Key Laboratory for Preparation and Application of Ordered Structural Materials of Guangdong Province, Shantou

University, 515063 Shantou, China

d School of Chemistry, South China Normal University, 510006 Guangzhou, China

Correspondence for materials should be addressed to X.Z. (zhuxj@ccelab.com.cn), Y.L. (liuyuan@ccelab.com.cn), and Z.D. ([zydong@ccelab.com.cn](mailto:zydong@ccelab.com.cn)).

**1. Experimental section**

**1.1 Materials:**

Ammonium molybdate tetrahydrate (H_24_Mo_7_N_6_O_24_·4H_2_O), Thiourea (CH_4_N_2_S), Sodium cholate hydrate (C_24_H_39_NaO_5_·xH_2_O), Ruthenium (III) chloride hydrate (RuCl_3_·xH_2_O), Cobalt chloride hexahydrate (CoCl_2_·6H_2_O), Iron chloride (FeCl_3_), Nickel (II) chloride (NiCl_2_), Copper (II) chloride (CuCl_2_), Sodium borohydride (NaBH_4_) purchased from Aladdin Chemical Co. Ethanol (99.7 %) was provided by Guangdong Guanghua Sci-tech Co., Ltd. (Shantou, China). The ultrapure water was produced by the Supergenie R150 water purifier from Shanghai Rephile Bioscience Co., Ltd. (Shanghai, China). All reagents were not further purified and were used directly.

**1.2 Experimental setup and instruments:**

USMR-1 and UMSR were provided by Shantou Moge Fluid Technology Co., Ltd. Scheme S1 is a schematic diagram of the exfoliation of MoS_2_ NFs. USMR-1 has a circular channel with a diameter of 4 mm, and its input and output ends are connected to hoses, respectively. A peristaltic pump transports the fluid from the beaker into the UMSR-1, and the output hose is connected to the beaker to ensure that the fluid can repeatedly enter the UMSR-1.^[1]^ The USMR system mainly consists of a microtube, a piezoelectric transducer (20 kHz), an ultrasonic generator, two syringe pumps, etc.^[2]^ As shown in Scheme S2, in this device, the quartz microtube is directly coupled to the piezoelectric transducer by epoxy glue (ENDFEST300, UHU, Germany) to establish USMR. The quartz microtube has an outer diameter of 3.5 mm, an inner diameter of 1 mm, and a length of 68 mm. It has an inlet of a T-joint (Scheme S2), which is connected to a FEP tube with an outer diameter of 5 mm to transport two fluids.

The morphology and microstructure of the material were characterized by transmission electron microscopy (TEM, Thermo Scientific Talos F200X G2) and scanning electron microscopy (SEM, Thermo Scientific Verios 5 UC). X-ray diffraction (XRD, DX-27 mini) is used to analyze the crystal structure. The excitation light source was Cu-Kα radiation, and the scanning range was 2θ = 5°–80°. The Raman spectra were recorded on a spectrometer (LabRAM Odyssey) operating the 532 nm. X-ray photoelectron spectroscopy (XPS) was performed using a monochromatic Al Kα X-ray source (Thermo Scientific EscaLab Xi+). All binding energies were calibrated by correcting the C 1s spectrum to 284.8 eV. Electrochemical measurements are carried out on a CHI660E electrochemical workstation (CH Instruments). The inductively coupled plasma mass spectrometry (ICP-MS) was performed on a Shimadzu ICPE-9820 using argon carrier gas. Tapping-mode atomic force microscopy (AFM) images were obtained on the Digital Instruments Oxford Cypher S Scanning Probe Microscope platform.

**1.3 Preparation of 1T phase MoS_2_ NSs:**

1T phase MoS_2_ nanoflowers (MoS_2_ NFs) were prepared by the hydrothermal method. 1.2359 g of ammonium molybdate [(NH_4_)_6_Mo_7_O_4_·2H_2_O] was added to ultrapure water, then 2.5119 g of thiourea was added and stirred for 2 hours. Subsequently, the precursor solution was shifted to an 80 mL autoclave and reacted at 180 °C for 18 h. The final sample was subsequently centrifuged with ultrapure water and alcohol 3 times and dried under vacuum at 60 °C for 6 h. Then, the obtained 1T phase MoS_2_ NFs were exfoliated to obtain 1T phase MoS_2_ nanosheets (MoS_2_ NSs). Sodium cholate was dispersed in 100 mL ultrapure water, and the concentration of the sodium cholate solution was 3 mg/mL. 1T phase MoS_2_ NFs were dispersed in sodium cholate solution, and the concentrations were 3 mg/mL, 5 mg/mL, and 10 mg/mL, respectively. The mixed solution was input into USMR-1 using a peristaltic pump and ultrasonicated for 2 hours, with an ultrasonic frequency of 20 kHz and an ultrasonic power of 200 W. The ultrasonicated sample was then centrifuged at 3000 rpm to collect the upper solution. The precipitate was further collected by centrifugation at 12000 rpm, washed three times with ultrapure water and alcohol, and dried to obtain 1T phase MoS_2_ NSs. The MoS_2_ NSs obtained after exfoliation of 1T phase MoS_2_ NFs were named E-MoS_2_ NSs.

**1.4 Preparation of RuCo@E-MoS_2-x_ NSs:**

E-MoS_2_ NSs were dispersed in 10 mL of deionized water with a concentration of 5 mg/mL. Then RuCl_3_·xH_2_O and CoCl_2_·6H_2_O were added and stirred to obtain a suspension. Suspension and 10 mL sodium borohydride solution with a concentration of 1 mg/mL were added to USMR. The product was collected, washed with deionized water and ethanol, and centrifuged 3 times. The centrifuged product was placed in a vacuum-drying oven and dried for 12 hours. The obtained solid was ground to obtain E-MoS_2_ NSs loaded with RuCo alloy. During the experiment, it was found that after RuCo alloy loading, E-MoS_2_ NSs produced more S vacancies, so the E-MoS_2_ NSs after alloy loading were changed to E-MoS_2-x_ NSs. When RuCl_3_·xH_2_O and CoCl_2_·6H_2_O were 0.01 mmol, respectively, the obtained E-MoS_2_ NSs loaded with RuCo alloy were named Ru_1_Co_1_-1@E-MoS_2-x_ NSs. When the amount of RuCl_3_·xH_2_O and CoCl_2_·6H_2_O is 0.02 mmol, respectively, the obtained E-MoS_2_ NSs loaded RuCo alloy is named Ru_1_Co_1_-2@E-MoS_2-x_ NSs, and so on. Experiments show that when the input amounts of RuCl_3_·xH_2_O and CoCl_2_·6H_2_O are 0.04 mmol, respectively, their performance is higher than others. To study the optimal ratio of ruthenium and cobalt, the total metal input amount is fixed, and the input amount of RuCl_3_·xH_2_O and CoCl_2_·6H_2_O at different ratios is calculated, respectively. And named Ru_3_Co_1_-4@E-MoS_2-x_ NSs, Ru_2_Co_1_-4@E-MoS_2-x_ NSs, Ru_1_Co_1_-4@E-MoS_2-x_ NSs, Ru_1_Co_2_-4@E-MoS_2-x_ NSs, Ru_1_Co_3_-4@E-MoS_2-x_ NSs, respectively. For comparison, RuCo alloy (RuCo) with a ratio of 2:1, E-MoS_2-x_ NSs loaded with Ru (Ru@E-MoS_2-x_ NSs), Co (Co@E-MoS_2-x_ NSs), and E-MoS_2-x_ NSs loaded with RuCo alloy (MR-Ru_2_Co_1_-4@E-MoS_2-x_ NSs) were prepared without ultrasound in USMR were prepared. The metal input amount of other comparative catalysts is consistent with that of Ru_2_Co_1_-4@E-MoS_2-x_ NSs. In addition, to explore the versatility of this method, we prepared E-MoS_2-x_ NSs loaded with RuFe, RuNi, and RuCu alloys. During the experiment, the cobalt source was replaced by other metal salts according to the method of preparing Ru_2_Co_1_-4@E-MoS_2-x_ NSs, and they were named Ru_2_Fe_1_-4@E-MoS_2-x_ NSs, Ru_2_Ni_1_-4@E-MoS_2-x_ NSs, and Ru_2_Cu_1_-4@E-MoS_2-x_ NSs respectively.

**1.5 Electrochemical measurements:**

All electrochemical tests were performed at room temperature, all samples were carried out in a typical three-electrode cell with 0.5 M H_2_SO_4_ and 1.0 M KOH, and all tests were performed on a CHI 760E electrochemical work. The obtained sample was used as the working electrode, while the graphite rod was used as the counter electrode, the saturated calomel electrode (SCE), and the Hg/HgO electrode were used as the reference electrode in acidic and alkaline electrolytes. During the measurement, N_2_ continuously entered the electrochemical electrolysis cell. The overpotential value is related to the reversible hydrogen electrode (RHE) as follows: E_RHE_=E_SCE_ + 0.241 V + 0.059 × pH. Preparation of the working electrode: (1) 2 mg of the catalyst was uniformly dispersed in 200 μL of isopropanol; (2) 2 μL of this solution was dropped onto a GCE with a diameter of 3 mm and then dried in air. The loading mass on the GCE was 0.281 mg·cm^−2^; (3) 2 μL of 1.0 wt% Nafion solution was dropped on top and dried. For linear sweep voltammetry (LSV) measurements, the scan rate was set to 0.5 mV·s^−1^ and corrected by 85% iR compensation. The Tafel slope was obtained from its corresponding LSV data by fitting the equation: $\eta=a+b*logj$. Electrochemical impedance spectroscopy (EIS) measurements were performed in the frequency range of 100 kHz to 1 Hz. Cyclic voltammetry (CV) curves were collected in the range of 0.191~0.291 V at different scan rates from 20 to 100 mV·s^-1^ compared with RHE.

**1.6 Calculation of electrochemical active surface area (ECSA)：**

ECSA of the electrocatalyst was calculated according to the following formula:

$$\text{ECSA=}\frac{\text{C}_{\text{dl}}}{\text{C}_{\text{s}}}\times\text{S}_{\text{geometric}}$$

Where C_s_ is 40 μF·cm^−2^ and S_geometric_ is the geometric area of the GCE (0.071 cm^2^). Cyclic voltammetry (CV) curves with different scan rates were conducted in the non-Faradic potential region of electrocatalysts. C_dl_ was the double-layer capacitance of the electrocatalyst calculated by plotting the (*j_a_–j_c_*)/2 vs. CV scan rate at the intermediate potential of the non-Faradic potential region. *j_a_* and *j_c_* represent the anodic and cathodic current density, respectively.

**1.7 Calculation of per-site TOF values：**

TOF is defined as the amount of H_2_ released per second from the active sites. The per-site TOF values can be calculated based on the following equation.

$\text{TOF=}\frac{\text{j}\times\text{A}}{2\times\text{n}\times\text{F}}$

Where j is the current density during the LSV measurement in 0.5 M H_2_SO_4_ and 1.0 M KOH solution. A stand for the area of the electrode is 0.071 cm^2^ and F is the Faradaic constant (96485 C·mol^−1^). 2 accounts for the electrons consumed to form the H_2_ molecule from water (2e^−^ for HER). n represents the number of active sites. This paper estimates TOF based on the assumption that all Ru atoms (determined by ICP-MS results) are exposed. The total number of Ru active sites on the electrode can be calculated from the total mass of Ru loaded and the atomic weight of Ru according to the following formula:

$$\text{n = }\frac{\text{m}_{\text{catalyst}}\times\text{C}_{\text{wt\%-Ru}}}{\text{M}_{\text{Ru}}}\times6.022\times10^{23}$$

Where m_catalyst_ is the catalyst loading on the NF electrode, C_wt%_ is the concentration of metal derived from ICP-MS results. Thus, the current density from the LSV curve can be converted into TOF values as follows:

$$\text{TOF=}\frac{3.12\times10^{15}}{\text{Number of Ru active sites}}\times\left|\text{j}\right|$$

**1.8 DFT calculations**

This study employs spin-polarized density functional theory (DFT) to perform structural optimizations and energy calculations. All computations were conducted using the Vienna Ab initio Simulation Package (VASP). The exchange-correlation interactions were described using the Perdew-Burke-Ernzerhof (PBE) functional within the generalized gradient approximation (GGA) framework. Atomic positions were optimized until the forces were less than 0.02eV/Å for the intermediate structures, and a plane-wave cutoff energy of 400 eV was employed. Free energy correction was performed at 298.15 K. Brillouin zone sampling was performed using a 2×2×1 Monkhorst-Pack k-point mesh. To eliminate artificial interactions induced by periodic boundary conditions, a vacuum layer exceeding 20 Å was implemented along the z-axis direction. Finally, the adsorption energies (E_ads_) are calculated as E_ads_ = E_ad/sub_ - E_ad_ - E_sub_, where E_ads_, E_ad,_ and E_sub_ are the optimized adsorbate/substrate system, the adsorbate in the structure and the clean substrate respectively. The free energy is calculated as follows:

G = E + ZPE - TS

where G, E, ZPE, and TS are the free energy, total energy from DFT calculations, zero-point energy, and entropic contributions, respectively.

**2. Results and Discussion**

**Note 1**

Scheme S1 outlines the preparation route of E-MoS_2_ nanosheets (NSs). Initially, MoS_2_ nanoflowers (NFs) containing 1T phase were synthesized via the hydrothermal method (Figure S1). Subsequently, MoS_2_ NFs were exfoliated using USMR-1. To ascertain the optimal exfoliation concentration, MoS_2_ NFs at varying concentrations (3 mg/mL, 5 mg/mL, and 10 mg/mL) were subjected to exfoliation, resulting in E-MoS_2_ NSs of different sizes. SEM images and statistical results (Figure S2) indicated that increasing the exfoliation concentration resulted in a reduction in nanosheet size, with values of 201.3 nm, 186.6 nm, and 134.1 nm, respectively. This occurred because ultrasound intensified collisions among MoS_2_ NFs at higher concentrations. Consequently, smaller E-MoS_2_ NSs were produced, exposing a greater number of edge active sites. Electrochemical test results (Figure S3) demonstrated that E-MoS_2_ NSs with an exfoliation concentration of 10 mg/mL had better HER performance. Thus, this exfoliation concentration was used for subsequent studies.

The phase structure of E-MoS_2_ NSs was characterized using X-ray diffraction (XRD) and Raman spectra. Figure S4(a) depicts the XRD patterns of 2H-MoS_2_, 1T-MoS_2_, and E-MoS_2_ NSs. The XRD pattern of E-MoS_2_ NSs exhibits diffraction peaks at 9.4° and 14.3°, corresponding to the characteristic peaks of 1T phase and 2H phase MoS_2_, respectively. The appearance of the characteristic peaks of the 2H phase is due to the transformation of the phase structure caused by the increase in temperature during the continuous ultrasonication process.^[3]^ Additionally, weak diffraction peaks in E-MoS_2_ NSs indicate reduced crystallinity. Raman spectroscopy (Figure S4(b)) further examined the phase structure of E-MoS_2_ NSs. The peaks at 378 cm^-1^ and 403 cm^-1^ correspond to the E^1^_2g_ (in-plane vibration) and A_1g_ (out-of-plane vibration) phonon modes of the 2H phase. At the same time, characteristic peaks of 1T phase MoS_2_ appear at 151 (J_1_ mode), 220 (J_2_ mode), 284 (E_1g_ mode), 335 (J_3_ mode), and 348 cm^-1^. The J_2_ mode, related to defects, indicates reduced crystallinity of E-MoS_2_ NSs post-ultrasonic treatment, promoting the creation of more active sites. Moreover, the E^1^_2g_ mode in E-MoS_2_ NSs shifts to a higher wavenumber than the 1T phase MoS_2_ before exfoliation. This indicates that the number of layers of E-MoS_2_ NSs is reduced.^[4]^ Atomic Force Microscope (AFM) results reveal that the thickness of E-MoS_2_ NSs is approximately 10 nm (Figure S5(a)). Additionally, transmission electron microscopy (TEM) images further confirm the flake morphology of E-MoS_2_ NSs (Figure S5(b)). Figure S5(c) illustrates that the (002) interplanar spacing of E-MoS_2_ NSs is 0.931 nm, significantly larger than that of the 2H phase MoS_2_. And the red circles indicate the typical defects of E-MoS_2_ NSs. To illustrate the effect of S vacancies on the HER activity of MoS_2_, three sets of comparative experiments were designed. (1) MoS_2_ nanoflowers prepared by direct hydrothermal synthesis; (2) MoS_2_ nanosheets (E-MoS_2_) after exfoliation in an ultrasonic microreactor; and (3) MoS_2_ nanosheets (E-MoS_2-x_) prepared by passing E-MoS_2_ and NaBH_4_ into an ultrasonic microreactor. XPS test results (see Figure S6 and Table S1) show that E-MoS_2-x_ has a higher S vacancy concentration. This is mainly because NaBH_4_ reacts with some S elements on the surface of E-MoS_2_. Its electrochemical HER activity was evaluated, and its HER performance (Figure S7) was better than that of E-MoS_2_ nanosheets and MoS_2_ nanospheres. At a current density of 10 mA·cm^-2^, its overpotential under acidic conditions was approximately 56 mV and 131 mV lower than the latter two, respectively. This strongly demonstrates the important role of S vacancies in improving the catalytic performance of MoS_2_.


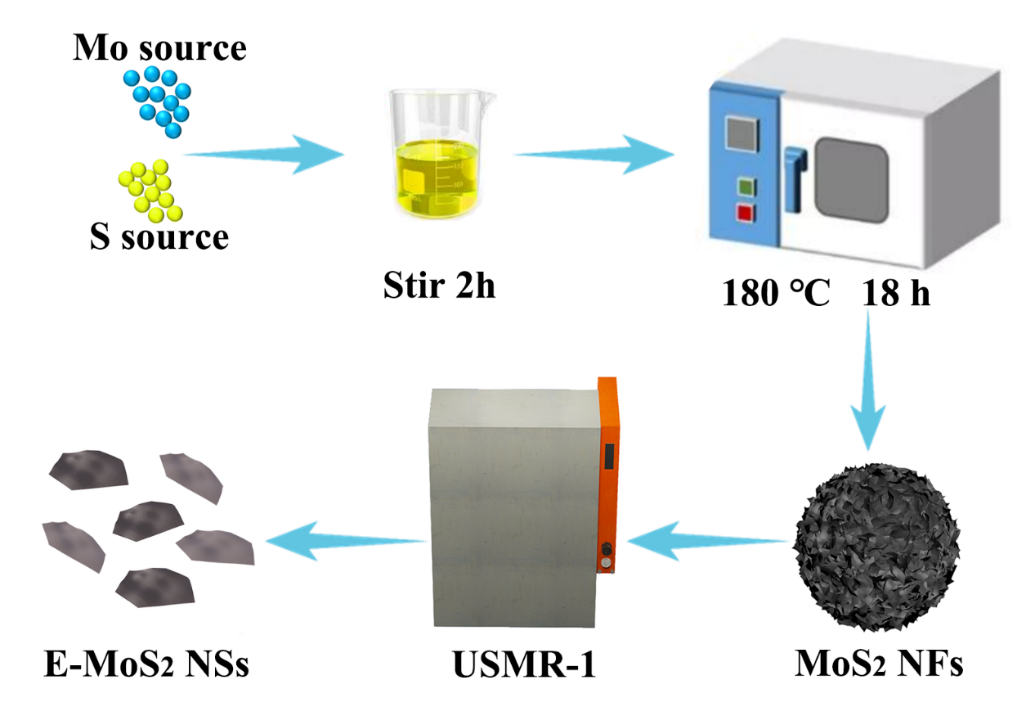


Scheme S1. Schematic illustration for the synthetic process of the E-MoS_2_ NSs.


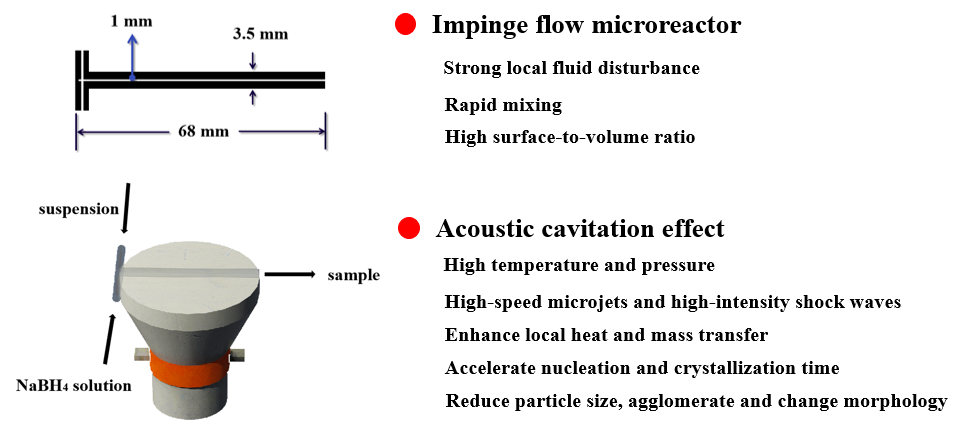


Scheme S2. Geometric information of the glass microtube (Top). Schematic of the USMR design (bottom).


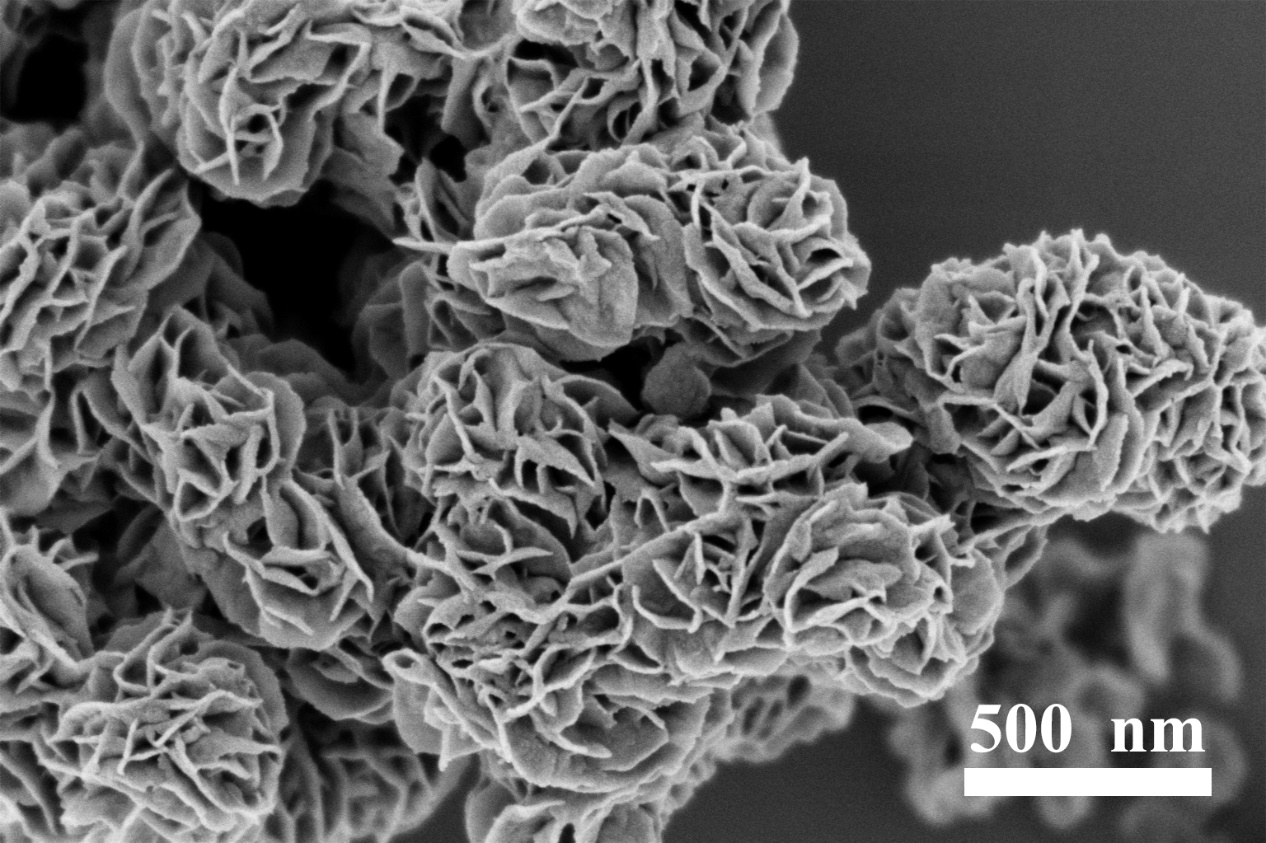


Figure S1. SEM image of the MoS_2_ NFs.


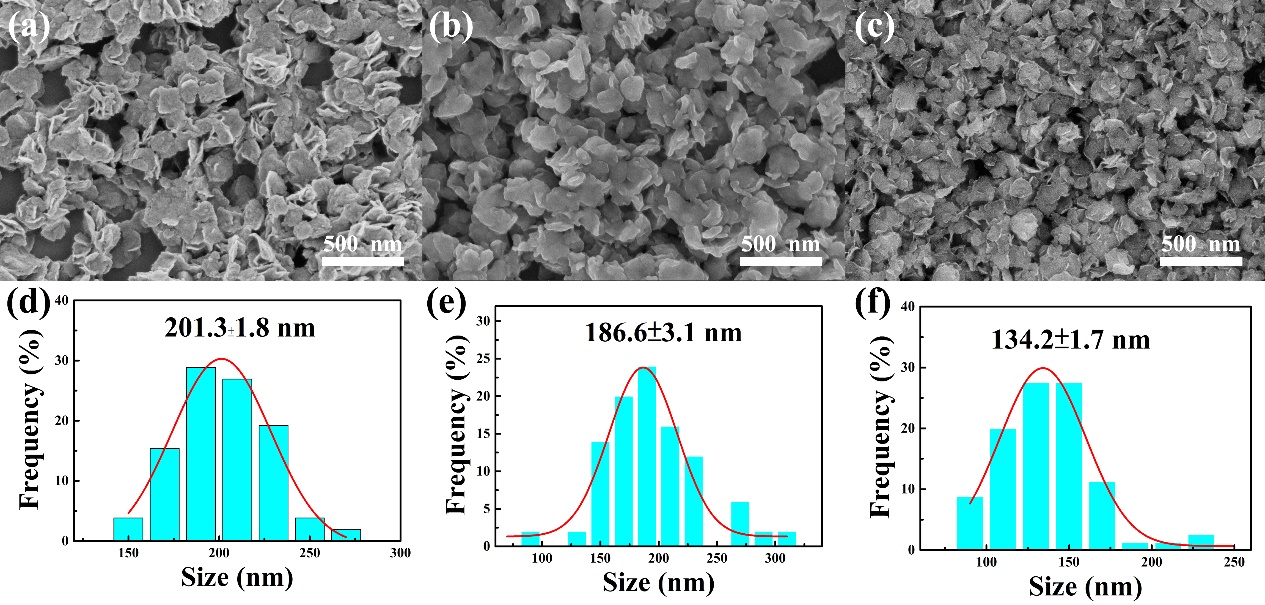


Figure S2. SEM images of E-MoS_2_ NSs exfoliation concentrations of (a) 3 mg/mL, (b) 5 mg/mL, and (c) 10 mg/mL. Size distribution of E-MoS_2_ NSs exfoliation concentrations of (d) 3 mg/mL, (e) 5 mg/mL, and (f) 10 mg/mL.





Figure S3. LSV curves of MoS_2_ NFs and E-MoS_2_ NSs exfoliated at 3 mg/mL, 5 mg/mL, and 10 mg/mL in 0.5 M H_2_SO_4_.


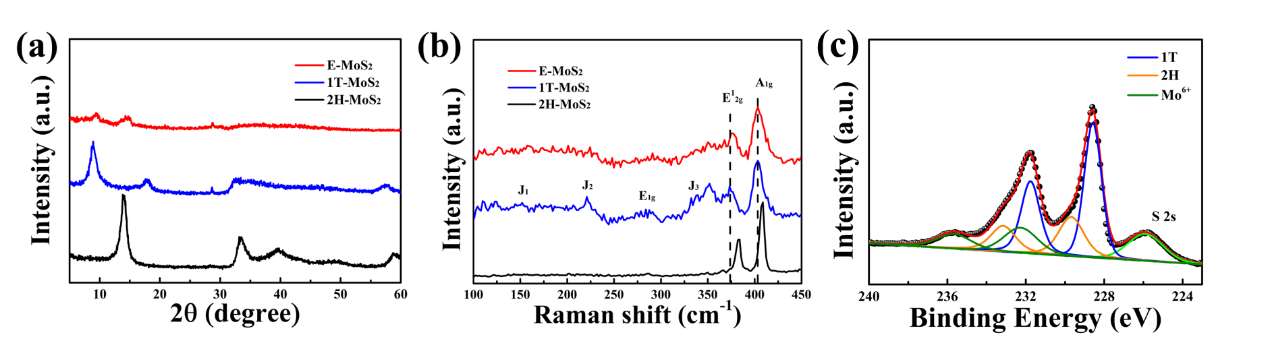


Figure S4. The (a) XRD and (b) Raman spectra of 2H phase MoS_2_, 1T phase MoS_2,_ and E-MoS_2_ NSs. (c) Mo 3d high-resolution XPS spectra of MoS_2_ NFs.


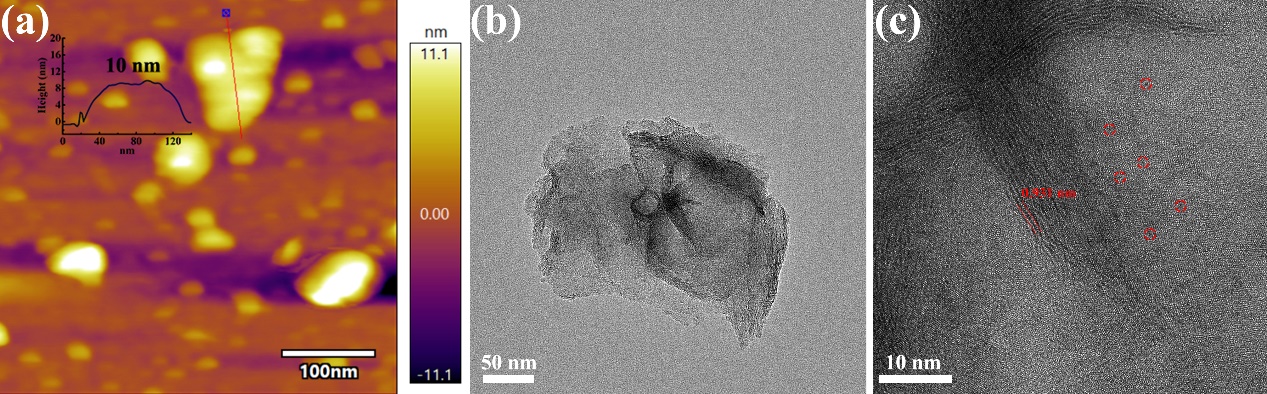


Figure S5. (a) AFM and (b)-(c) TEM spectra of E-MoS_2_ NSs.


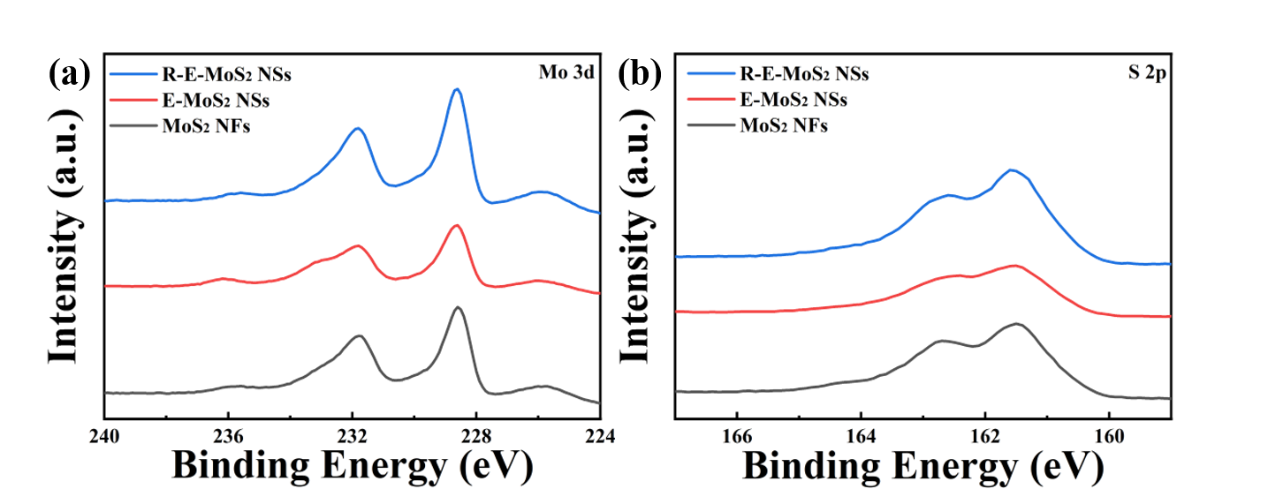


Figure S6. (a) Mo 3d and (b) S 2p high-resolution XPS spectra of MoS_2_ NFs, E-MoS_2_ NSs, and E-MoS_2-x_ NSs.





Figure S7. LSV curves of MoS_2_ NFs, E-MoS_2_ NSs, and E-MoS_2-x_ NSs in 0.5 M H_2_SO_4_.

Table S1. Concentrations of Mo, S, O, and C elements, as well as S vacancy concentration in MoS_2_ NFs, E-MoS_2_ NSs, and E-MoS_2-x_ NSs.

|  | Mo | S | O | C | S vacancy |
| --- | --- | --- | --- | --- | --- |
| MoS_2_ NFs | 10.92 | 19.73 | 49..57 | 19.78 | 9.7% |
| E-MoS_2_ NSs | 12.56 | 20.74 | 19.74 | 46.95 | 17.4% |
| E-MoS_2-x_ NSs | 21.34 | 34.52 | 19.69 | 24.45 | 19.1% |

Table S2. Concentrations of Ru, Co, Mo, and S elements in Ru_1_Co_1_-1@E-MoS_2-x_ NSs samples at different flow rates.

|  | Ru | Co | Mo | S |
| --- | --- | --- | --- | --- |
| 5 mL/min | 6.13 | 6.51 | 38.57 | 48.79 |
| 10 mL/min | 6.26 | 5.31 | 38.70 | 49.73 |
| 15 mL/min | 6.60 | 4.89 | 39.13 | 49.38 |
| 20 mL/min | 4.71 | 4.50 | 38.78 | 52.02 |

Table S3. Concentrations of Ru, Co, Mo, and S elements in Ru_1_Co_1_-1@E-MoS_2-x_ NSs samples at different ultrasonic powers.

|  | Ru | Co | Mo | S |
| --- | --- | --- | --- | --- |
| 20 W | 2.53 | 5.27 | 35.36 | 56.84 |
| 40 W | 4.71 | 4.50 | 38.78 | 52.02 |
| 60 W | 5.14 | 3.68 | 39.44 | 51.74 |

**
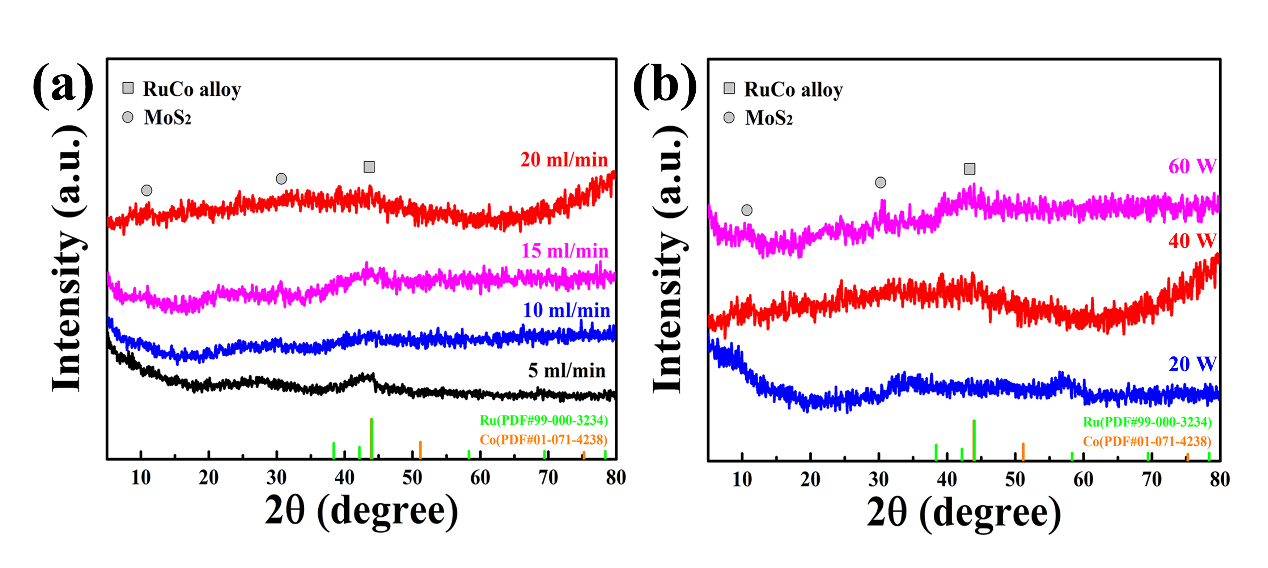
**

Figure S8. (a) XRD patterns of Ru_1_Co_1_-1@E-MoS_2-x_ NSs at different flow rates. (b) XRD patterns of Ru_1_Co_1_-1@E-MoS_2-x_ NSs at different ultrasonic powers.


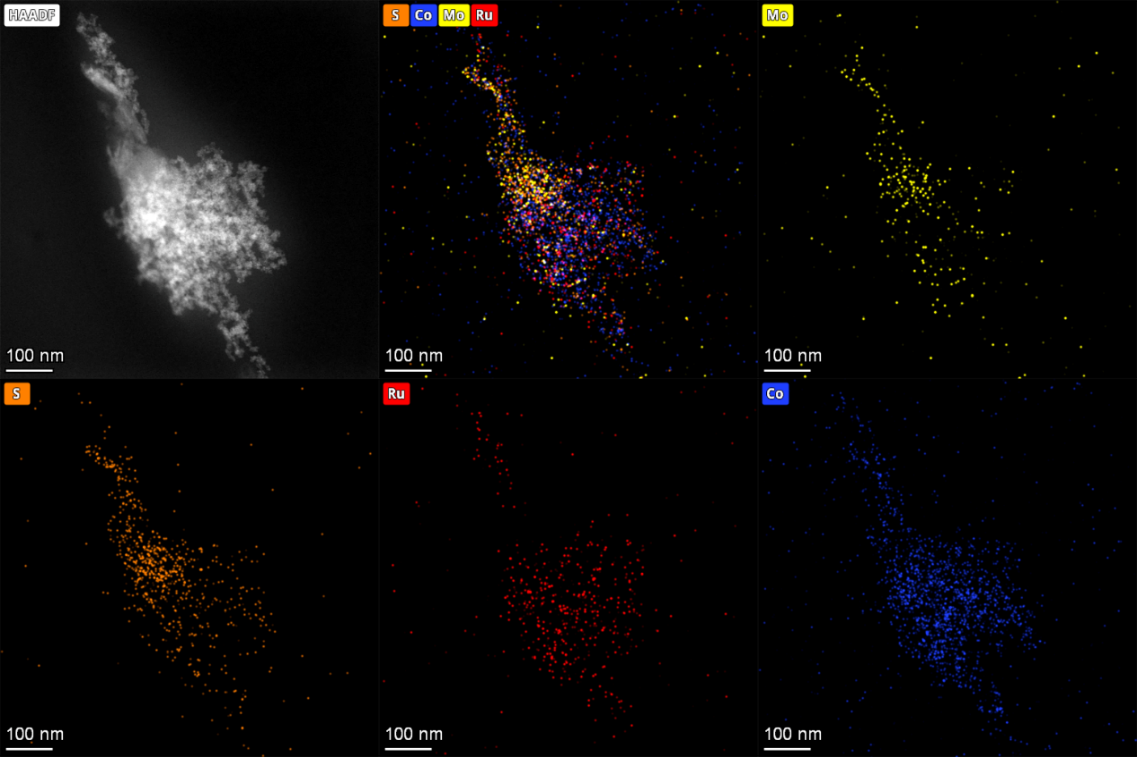


Figure S9. EDX elemental mapping image of Ru_1_Co_1_-1@E-MoS_2-x_ NSs (flow rate of 5 mL/min, ultrasonic power of 40 W).


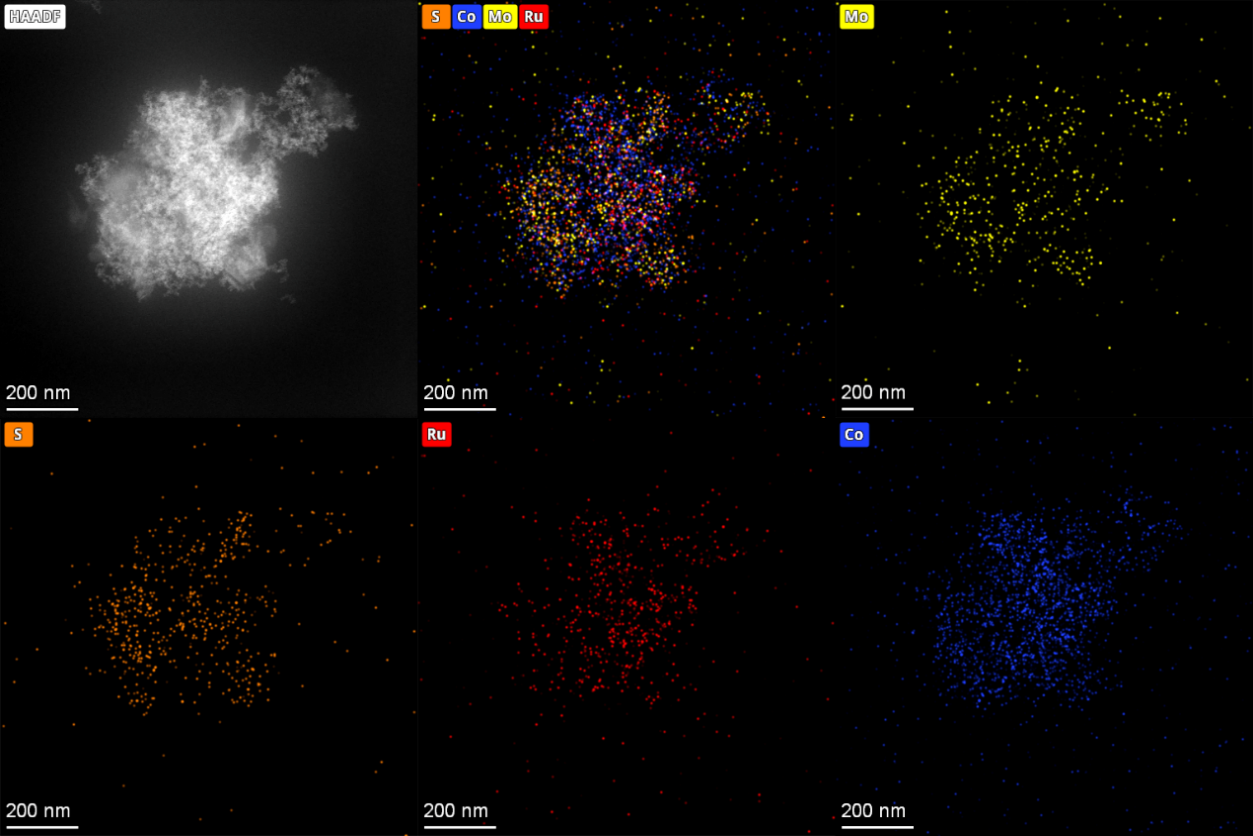


Figure S10. EDX elemental mapping image of Ru_1_Co_1_-1@E-MoS_2-x_ NSs (flow rate of 10 mL/min, ultrasonic power of 40 W).


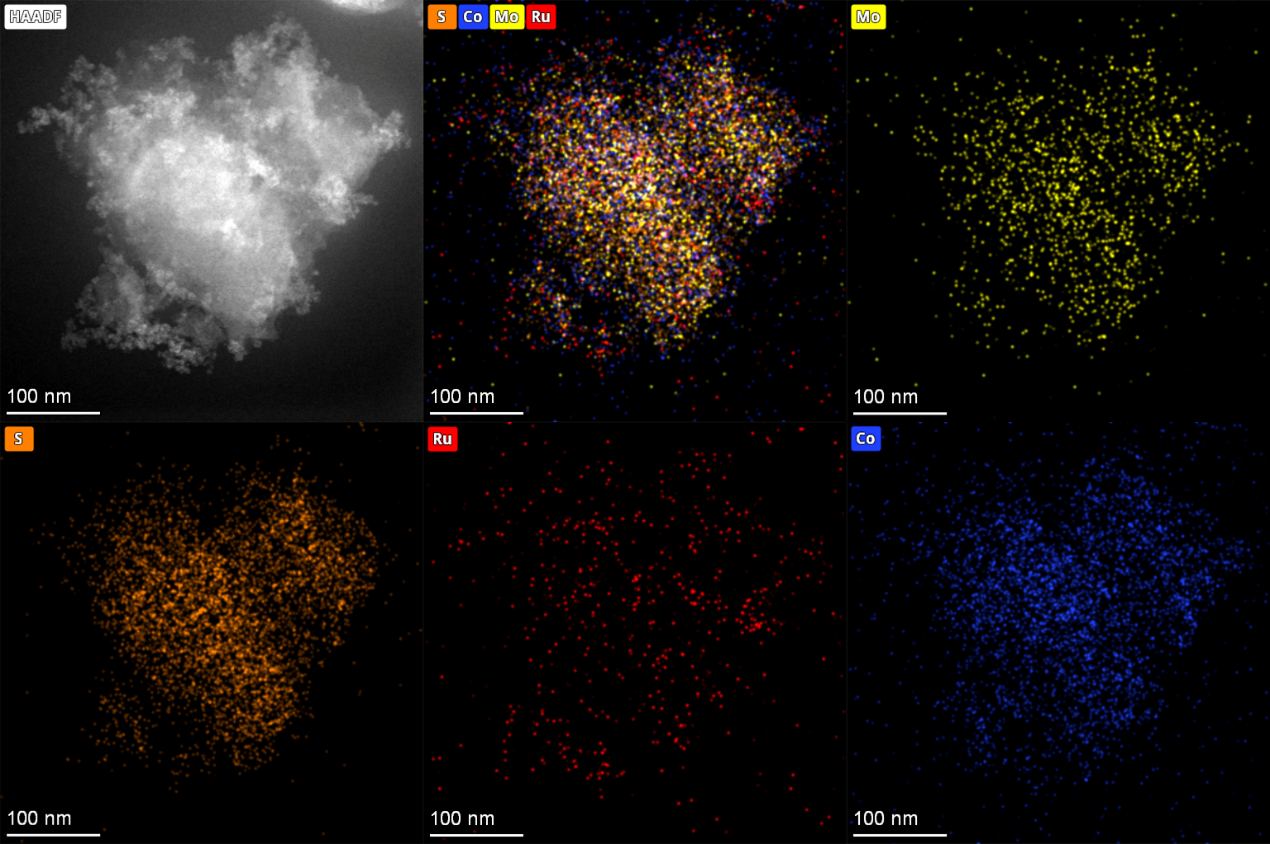


Figure S11. EDX elemental mapping image of Ru_1_Co_1_-1@E-MoS_2-x_ NSs (flow rate of 15 mL/min, ultrasonic power of 40 W).


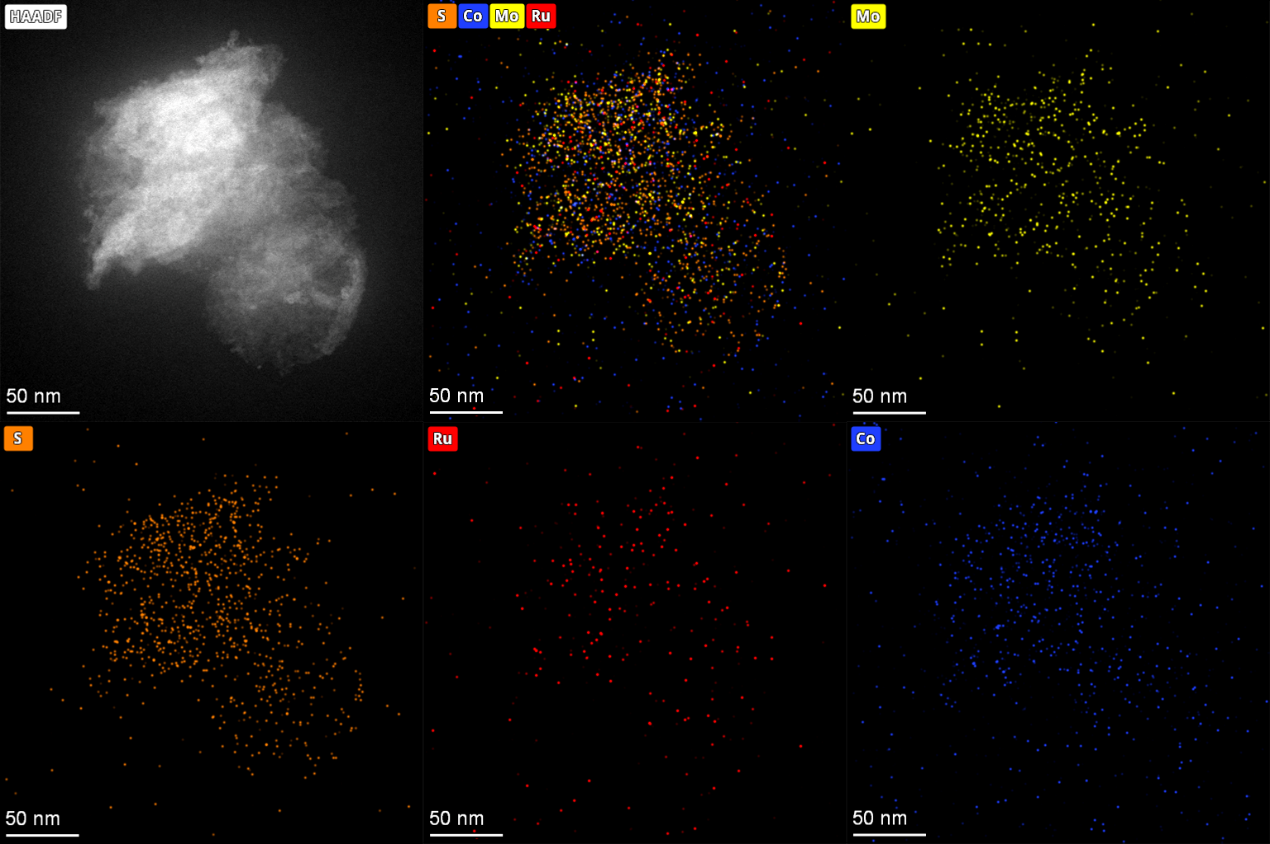


Figure S12. EDX elemental mapping image of Ru_1_Co_1_-1@E-MoS_2-x_ NSs (flow rate of 20 mL/min, ultrasonic power of 40 W)


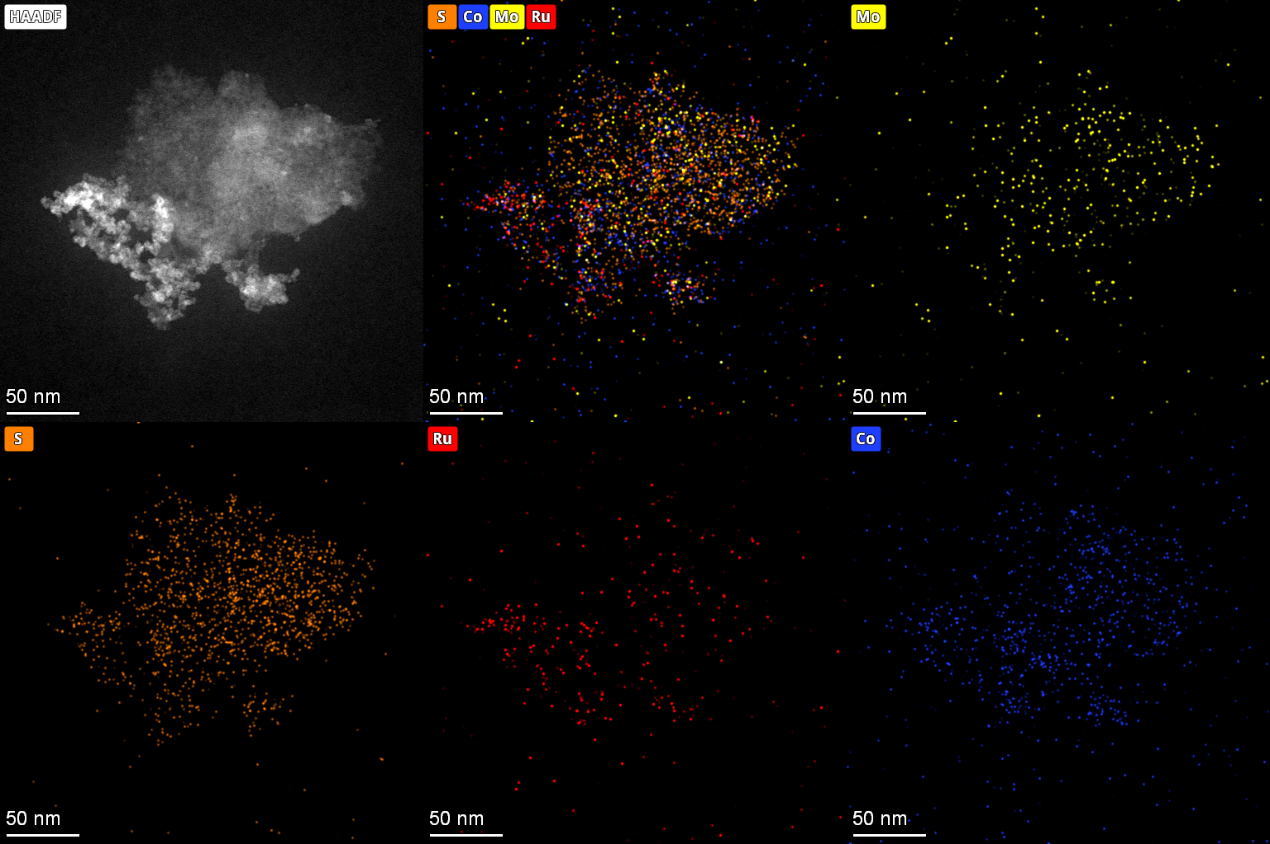


Figure S13. EDX elemental mapping image of Ru_1_Co_1_-1@E-MoS_2-x_ NSs (flow rate of 20 mL/min, ultrasonic power of 20 W)


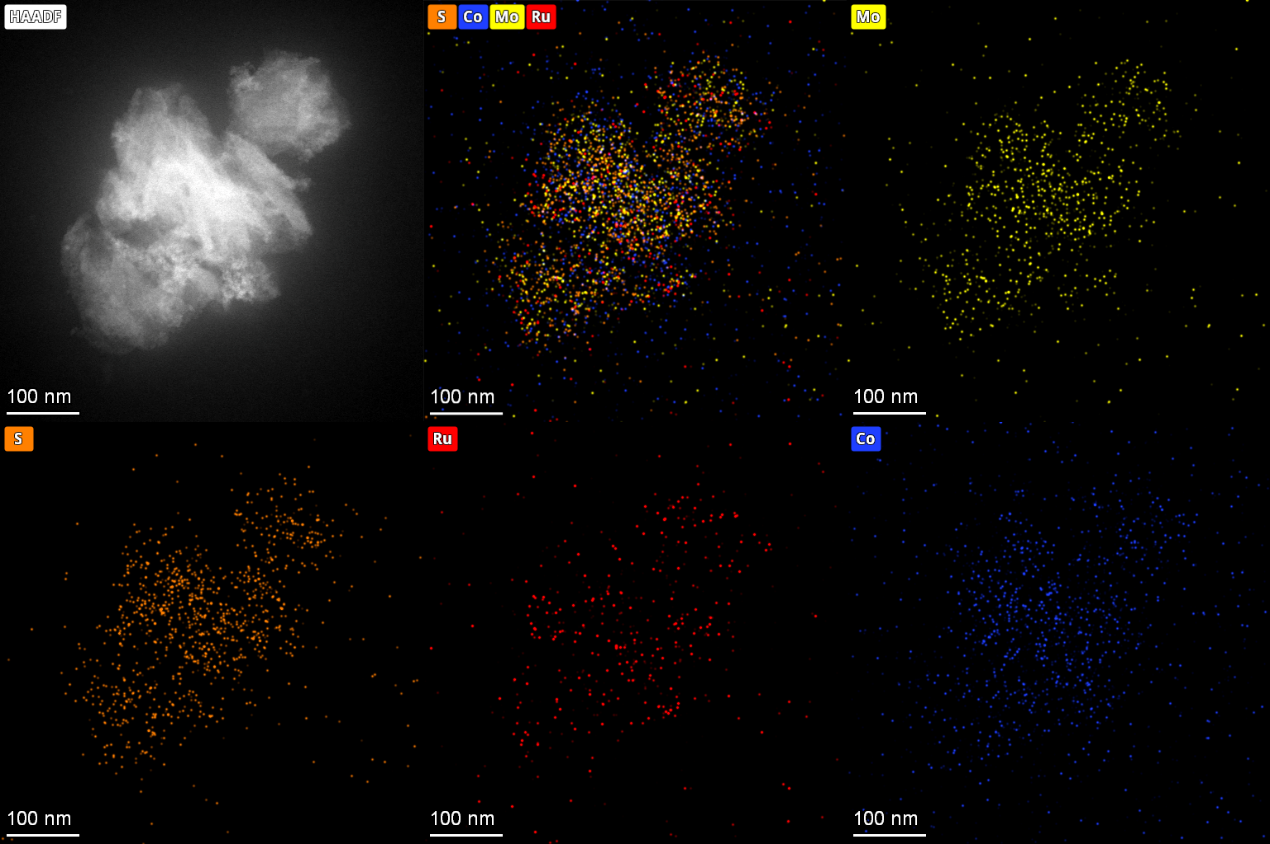


Figure S14. EDX elemental mapping image of Ru_1_Co_1_-1@E-MoS_2-x_ NSs (flow rate of 20 mL/min, ultrasonic power of 60 W).


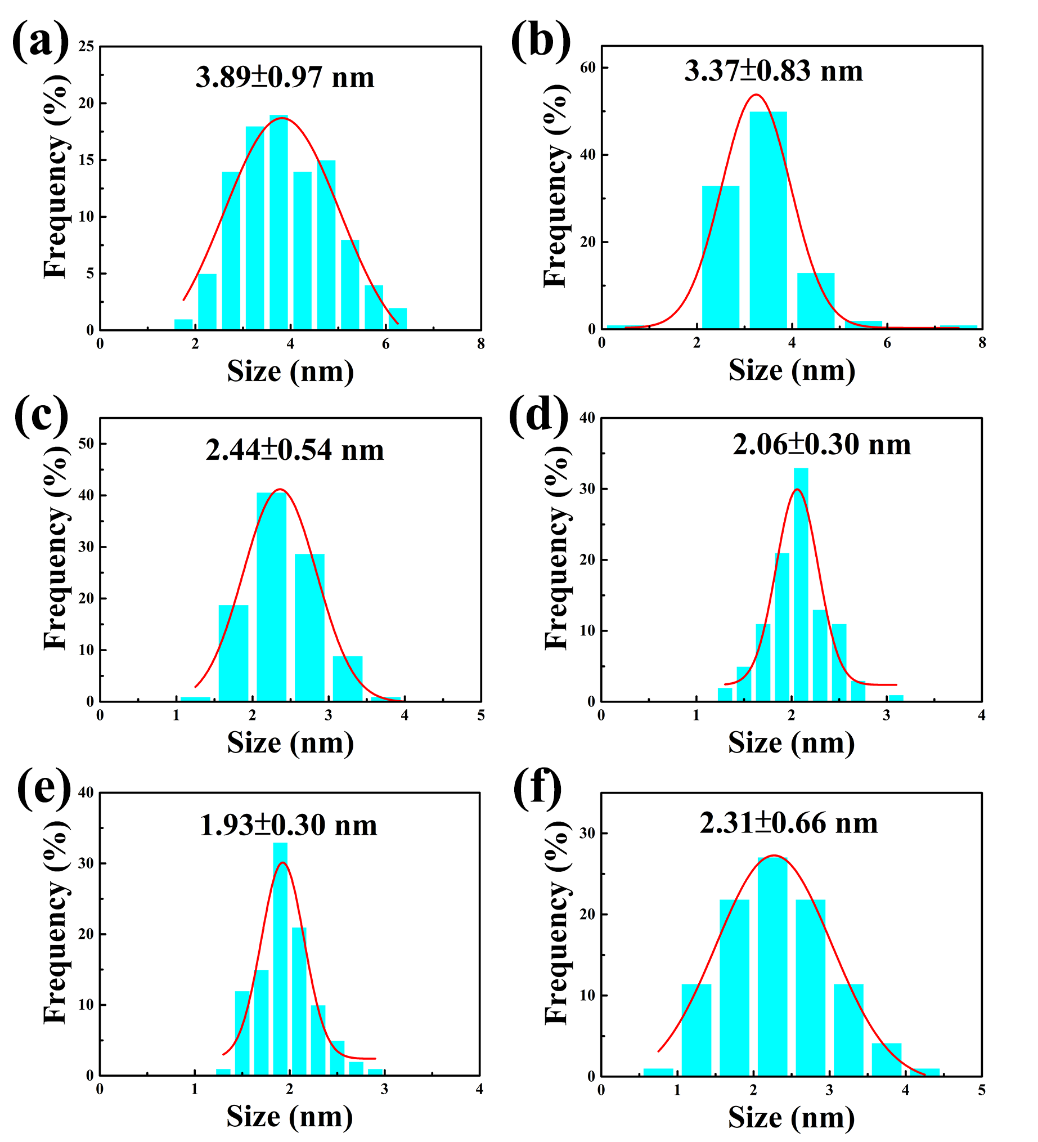


Figure S15. Ultrasonic power of 40 W, size distribution of Ru_1_Co_1_-1@E-MoS_2-x_ NSs at different flow rates (flow rates are (a) 5 mL/min, (b) 10 mL/min, (c) 15 mL/min, (d) 20 mL/min). Flow rate of 20 mL/min, size distribution of Ru_1_Co_1_-1@E-MoS_2-x_ NSs at different ultrasonic powers (powers are (e) 20 W, (f) 60 W).

.


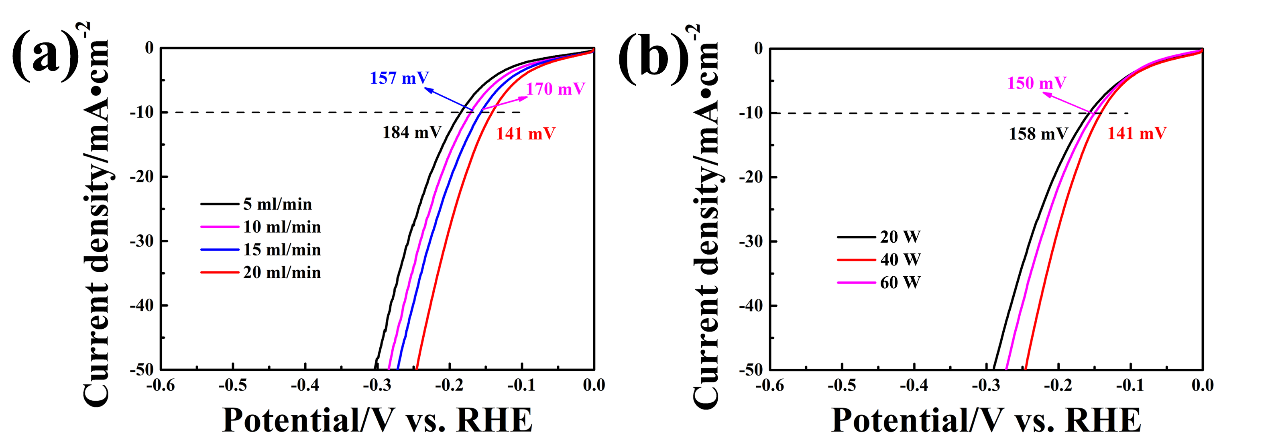


Figure S16. (a) LSV curves of Ru_1_Co_1_-1@E-MoS_2-x_ NSs at different flow rates. (b) LSV curves of Ru_1_Co_1_-1@E-MoS_2-x_ NSs at different ultrasonic powers.

**Note 2**

TEM images, HAADF-STEM images, and corresponding EDX element mapping images (Figure 2(a)-(b)) directly show monodispersed, small-sized RuCo alloy nanoparticles on Ru_2_Co_1_-4@E-MoS_2-x_ NSs. The average nanoparticle diameter measured 1.74 nm (Figure 2(b)), with a lattice spacing of 0.225 nm (Figure 2(b)). It is between 0.204 nm of monometallic Ru (101) grains and 0.244 nm of monometallic Co (111) grains. This belongs to the (100) crystal plane of the RuCo alloy with hcp structure, which conforms to Vegard's law.^[5]^ This is in good agreement with the XRD patterns (Figures S17 and S23). The line scan spectrum in Figure 2(b) further confirms that the RuCo alloy particles are loaded on E-MoS_2-x_ NSs. The XRD spectrum and XPS results (Figure S17 and Table S4) indicate that the loading of RuCo alloy on E-MoS_2-x_ NSs increases progressively with the metal precursor amount. Further ICP analysis (Figure S18) revealed that the alloy loading of Ru_1_Co_1_-4@E-MoS_2-x_ NSs and Ru_1_Co_1_-5@E-MoS_2-x_ NSs is similar. To comprehensively assess the HER activity of different alloy loadings, electrochemical tests were conducted under acidic and alkaline conditions, respectively. The results are depicted in Figures S19-S22. Ru_1_Co_1_-4@E-MoS_2-x_ NSs and Ru_1_Co_1_-5@E-MoS_2-x_ NSs exhibit similar overpotentials (𝜂=10 mA·cm^-2^), Tafel slopes, and electrochemically active areas (ECSA). This is mainly because the alloy loading has reached saturation on the Ru_1_Co_1_-4@E-MoS_2-x_ NSs sample. Additionally, the metal ratio of the RuCo alloy was further investigated under optimal metal loading. As shown in the corresponding XRD spectrum and XPS results (Figure S23 and Table S5), E-MoS_2-x_ NSs successfully loaded RuCo alloy particles with varying metal ratios. The Ru 3p high-resolution XPS spectrum (Figures S23b-c) exhibits that there are two well-separated main peaks near 462.6 and 484.7 eV, corresponding to metallic Ru (Ru^0^), while the other two peaks at 464.5 and 488.1 eV belong to oxidized Ru (Ru^n+^) species.^[6]^ Compared with other E-MoS_2-x_ NSs loaded with RuCo alloys of different metal ratios, in Ru_2_Co_1_-4@E-MoS_2-x_ NSs, the 3p orbitals of Ru^0^ and Ru^n^ shift to lower binding energy, and the Co 2p orbital shifts to a higher binding energy. This indicates that electron transfer from Co atoms to Ru atoms alters the electronic structure of both, creating a strong interaction between Ru and Co atoms. Furthermore, the strong electronic interaction between Ru and Co atoms further confirms the formation of the RuCo alloy. This interaction also enhances charge transfer between atoms, thereby enhancing its catalytic activity.^[5b, 7]^ However, no strong interaction was observed in the XPS spectrum of Ru_3_Co_1_-4@E-MoS_2-x_ NSs. This may be due to the low concentration of Co atoms, resulting in a weaker interaction. The HER activity of E-MoS_2-x_ NSs loaded with RuCo alloys of varying metal ratios was evaluated. The results are depicted in Figure S24. Ru_2_Co_1_-4@E-MoS_2-x_ NSs exhibit the lowest overpotential and Tafel slope in 0.5 M H_2_SO_4_ and 1.0 M KOH solutions. CV curves and corresponding ECSA values ​​indicate that Ru_2_Co_1_-4@E-MoS_2-x_ NSs possess a significant active surface area (Figures S25-S26). This is primarily attributed to the strong interaction between Ru atoms and Co atoms in RuCo alloys. In addition, in Ru_3_Co_1_-4@E-MoS_2-x_ NPs, excessive Ru leads to strong adsorption of H on the catalyst surface, limiting the release of H_2_ and thus reducing the HER activity.


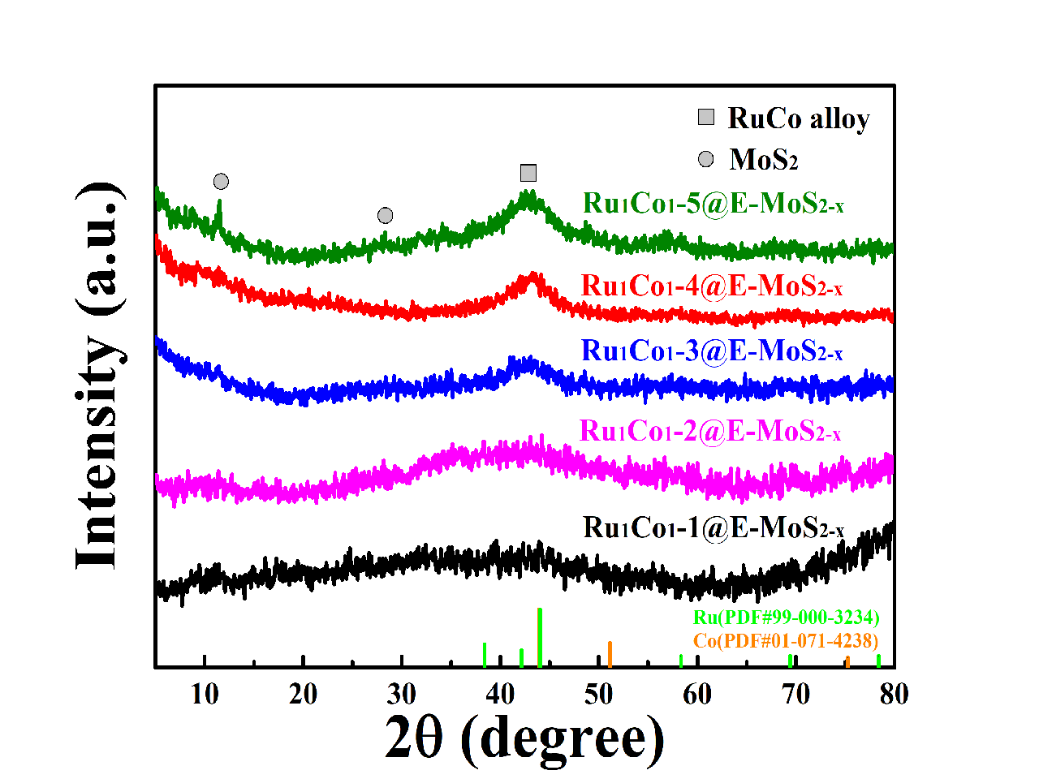


Figure S17. XRD patterns of RuCo@E-MoS_2-x_ NSs with different metal loading amounts.

Table S4. Concentrations of Ru, Co, Mo, and S elements in Ru_1_Co_1_-1@E-MoS_2-x_ NSs with different metal loading amounts

|  | Ru | Co | Mo | S |
| --- | --- | --- | --- | --- |
| Ru_1_Co_1_-1@E-MoS_2-x_ | 4.71 | 4.50 | 38.78 | 52.02 |
| Ru_1_Co_1_-2@E-MoS_2-x_ | 6.62 | 6.23 | 38.20 | 48.95 |
| Ru_1_Co_1_-3@E-MoS_2-x_ | 7.66 | 7.43 | 38.72 | 46.42 |
| Ru_1_Co_1_-4@E-MoS_2-x_ | 8.11 | 9.27 | 34.94 | 47.68 |
| Ru_1_Co_1_-5@E-MoS_2-x_ | 12.31 | 11.16 | 32.73 | 43.79 |


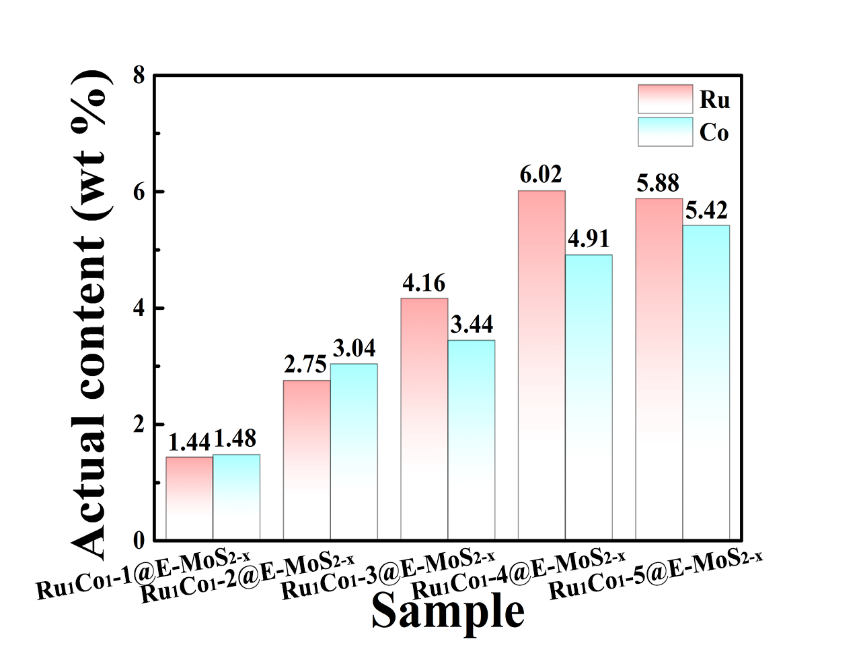


Figure S18. The Ru and Co element contents in RuCo@E-MoS_2-x_ NSs with different metal loading amounts samples tested by the ICP-MS method.


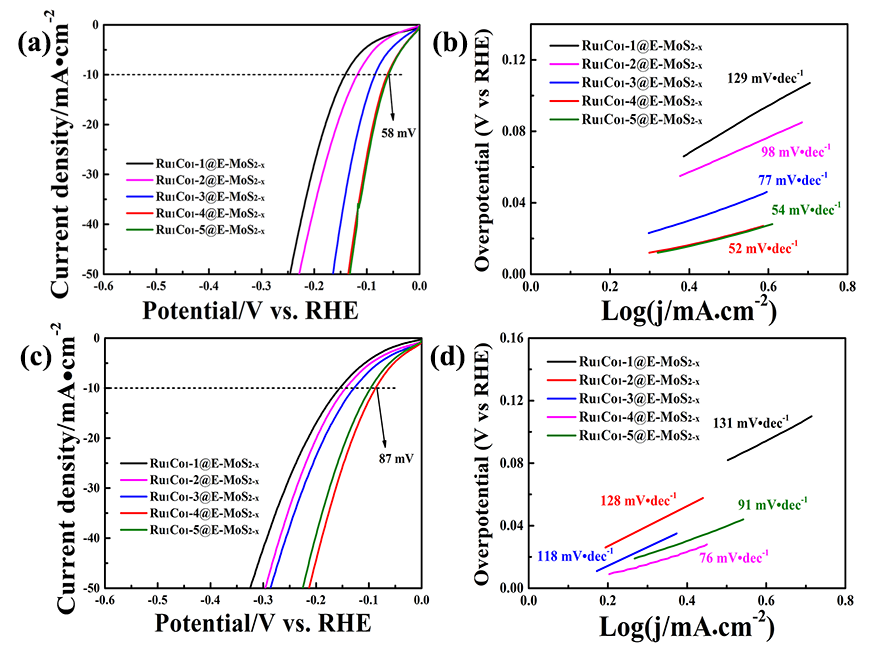


Figure S19. (a) LSV curves and (b) Tafel plots of RuCo@E-MoS_2-x_ NSs with different metal loading amounts in 0.5 M H_2_SO_4_.


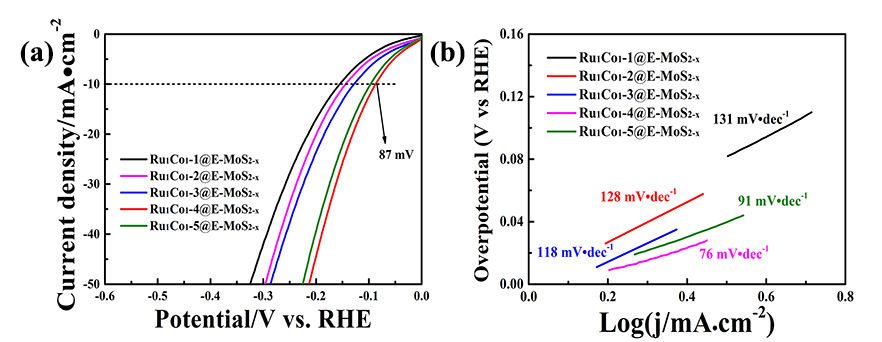


Figure S20. (a) LSV curves and (b) Tafel plots of RuCo@E-MoS_2-x_ NSs with different metal loading amounts in 1.0 M KOH.


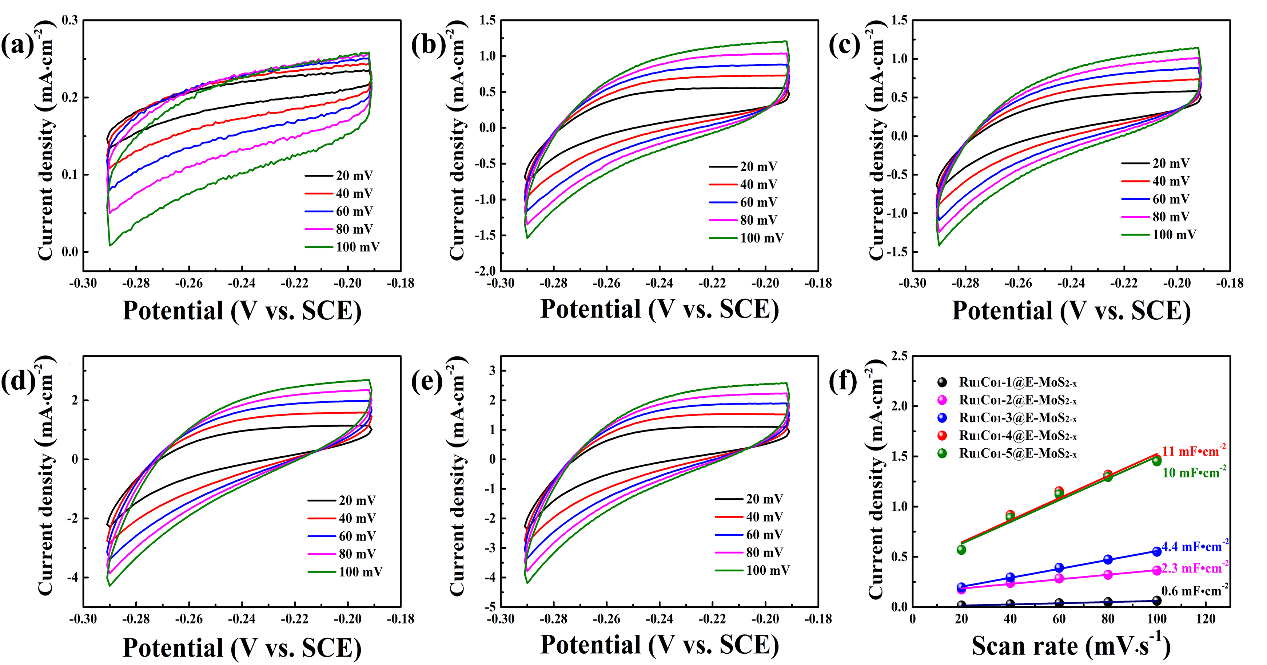


Figure S21. CV curves of the (a) Ru_1_Co_1_-1@E-MoS_2-x_ NSs, (b) Ru_1_Co_1_-2@E-MoS_2-x_ NSs, (c) Ru_1_Co_1_-3@E-MoS_2-x_ NSs, (d) Ru_1_Co_1_-4@E-MoS_2-x_ NSs, and (e) Ru_1_Co_1_-5@E-MoS_2-x_ NSs in 0.5 M H_2_SO_4_. (e) C_dl_ values of these samples in 0.5 M H_2_SO_4_.


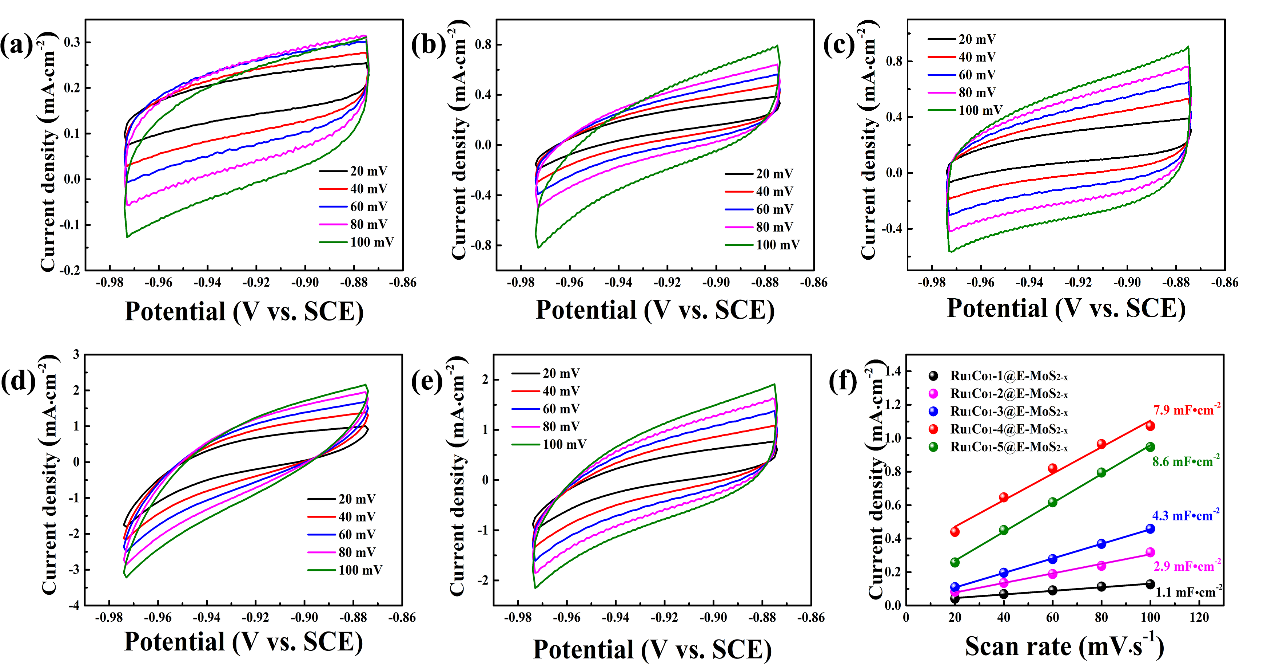


Figure S22. CV curves of the (a) Ru_1_Co_1_-1@E-MoS_2-x_ NSs, (b) Ru_1_Co_1_-2@E-MoS_2-x_ NSs, (c) Ru_1_Co_1_-3@E-MoS_2-x_ NSs, (d) Ru_1_Co_1_-4@E-MoS_2-x_ NSs, and (e) Ru_1_Co_1_-5@E-MoS_2-x_ NSs 1.0 M KOH. (e) C_dl_ values of these samples 1.0 M KOH.


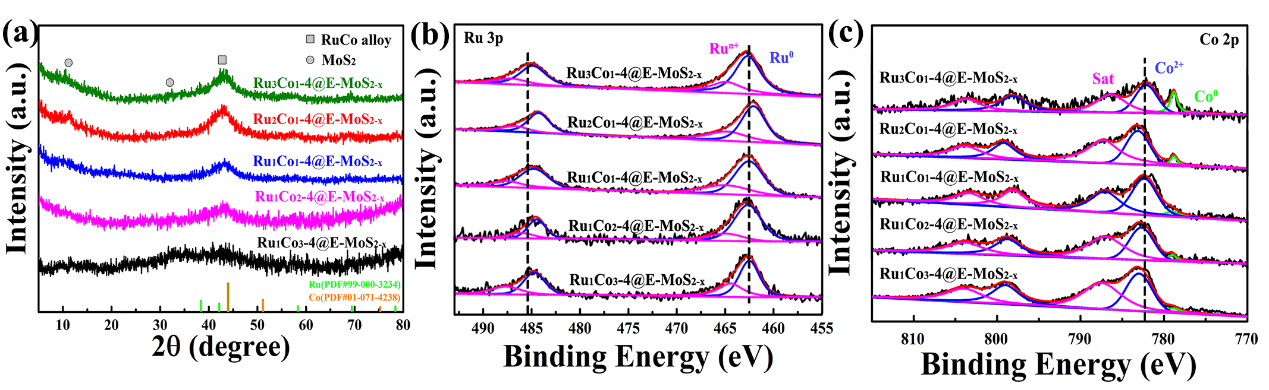


Figure S23. (a) XRD patterns, (b) Ru 3p and (c) Co 2p high-resolution XPS spectra of RuCo@E-MoS_2-x_ NSs loaded with varying metal ratios.

Table S5. Concentrations of Ru, Co, Mo, and S elements in RuCo@E-MoS_2-x_ NSs loaded with varying metal ratios.

|  | Ru | Co | Mo | S |
| --- | --- | --- | --- | --- |
| Ru_1_Co_3_-4@E-MoS_2-x_ | 3.74 | 16.39 | 27.62 | 52.25 |
| Ru_1_Co_2_-4@E-MoS_2-x_ | 3.22 | 6.81 | 35.86 | 54.12 |
| Ru_1_Co_1_-4@E-MoS_2-x_ | 8.11 | 9.27 | 34.94 | 47.68 |
| Ru_2_Co_1_-4@E-MoS_2-x_ | 14.86 | 9.39 | 28.18 | 47.57 |
| Ru_3_Co_1_-4@E-MoS_2-x_ | 12.76 | 4.67 | 35.18 | 47.40 |


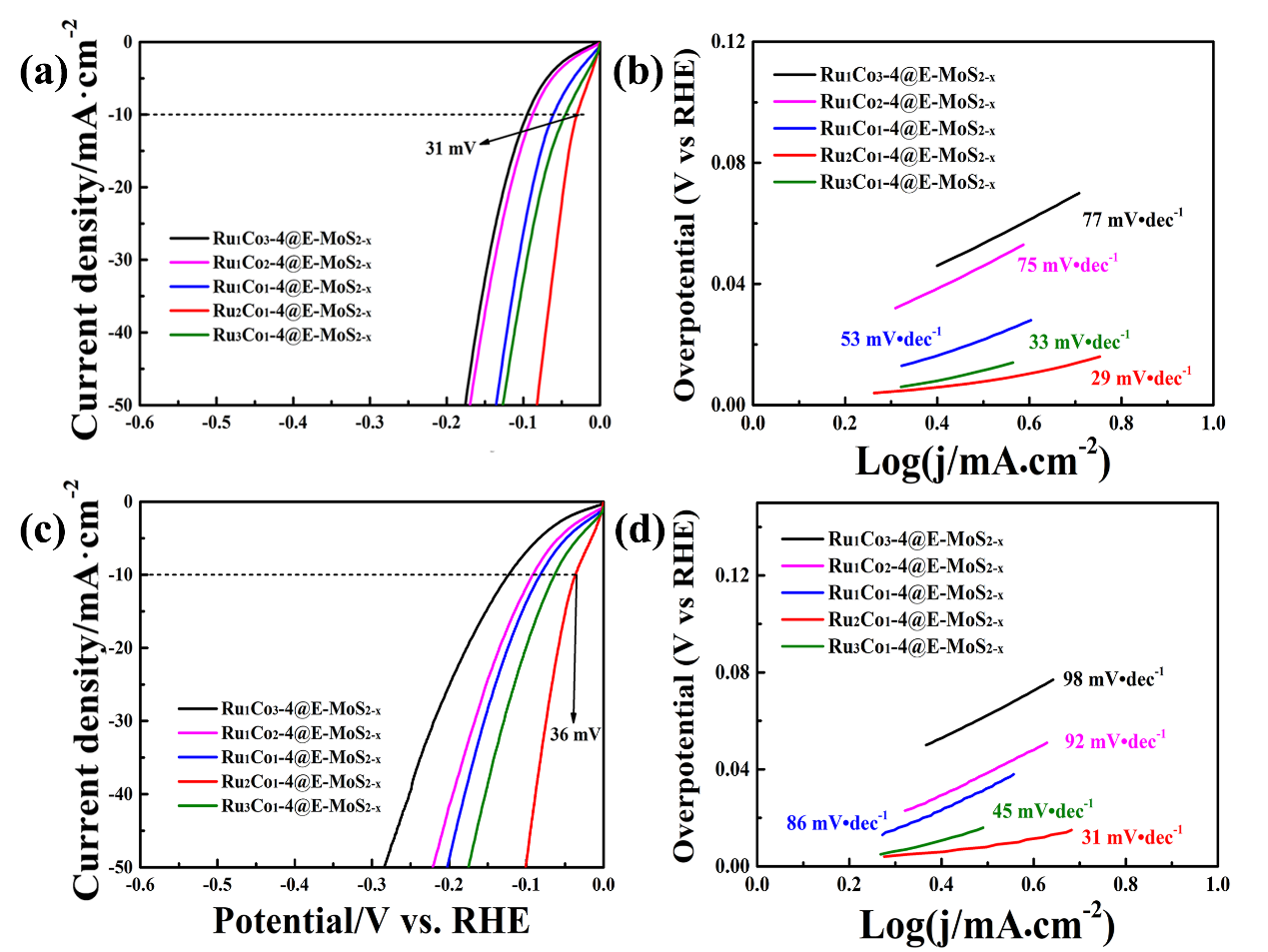


Figure S24. (a) LSV curves and (b) Tafel plots of RuCo@E-MoS_2-x_ loaded with varying metal ratios in 0.5 M H_2_SO_4_. (c) LSV curves and (d) Tafel plots of RuCo@E-MoS_2-x_ loaded with varying metal ratios in 1.0 M KOH.


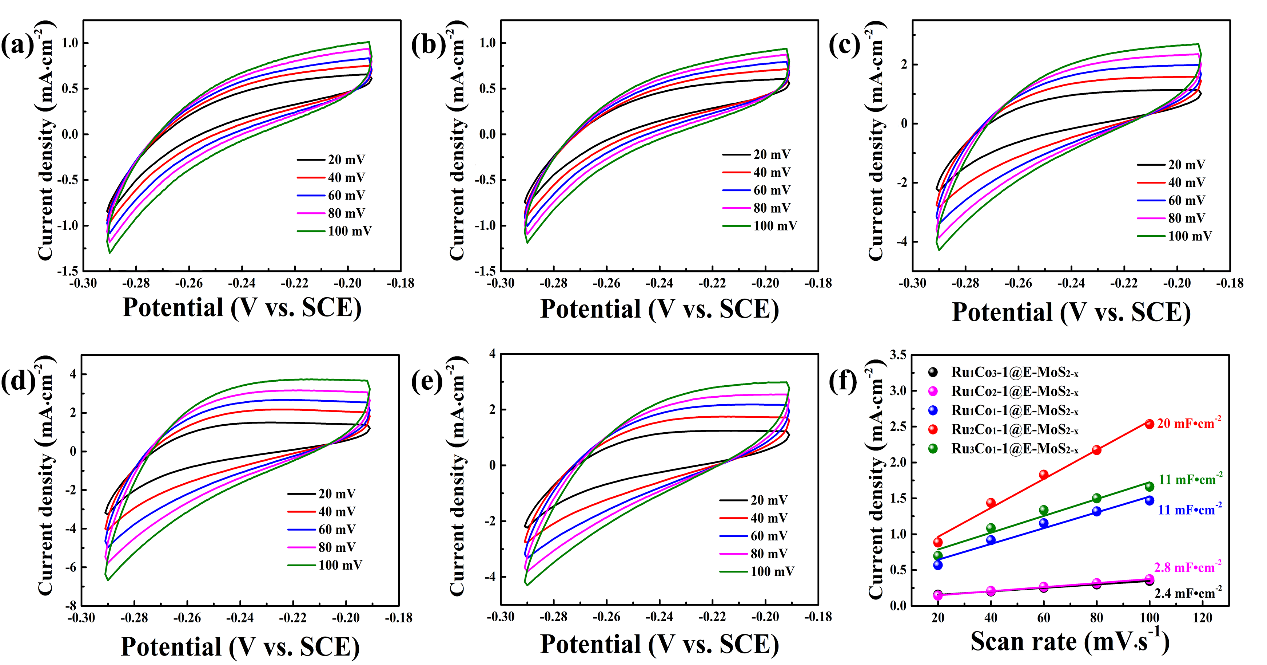


Figure S25. CV curves of the (a) Ru_1_Co_3_-4@E-MoS_2-x_ NSs, (b) Ru_1_Co_2_-4@E-MoS_2-x_ NSs, (c) Ru_1_Co_1_-4@E-MoS_2-x_ NSs, (d) Ru_2_Co_1_-4@E-MoS_2-x_ NSs, and (e) Ru_3_Co_1_-4@E-MoS_2-x_ NSs in 0.5 M H_2_SO_4_. (e) C_dl_ values of these samples in 0.5 M H_2_SO_4_.


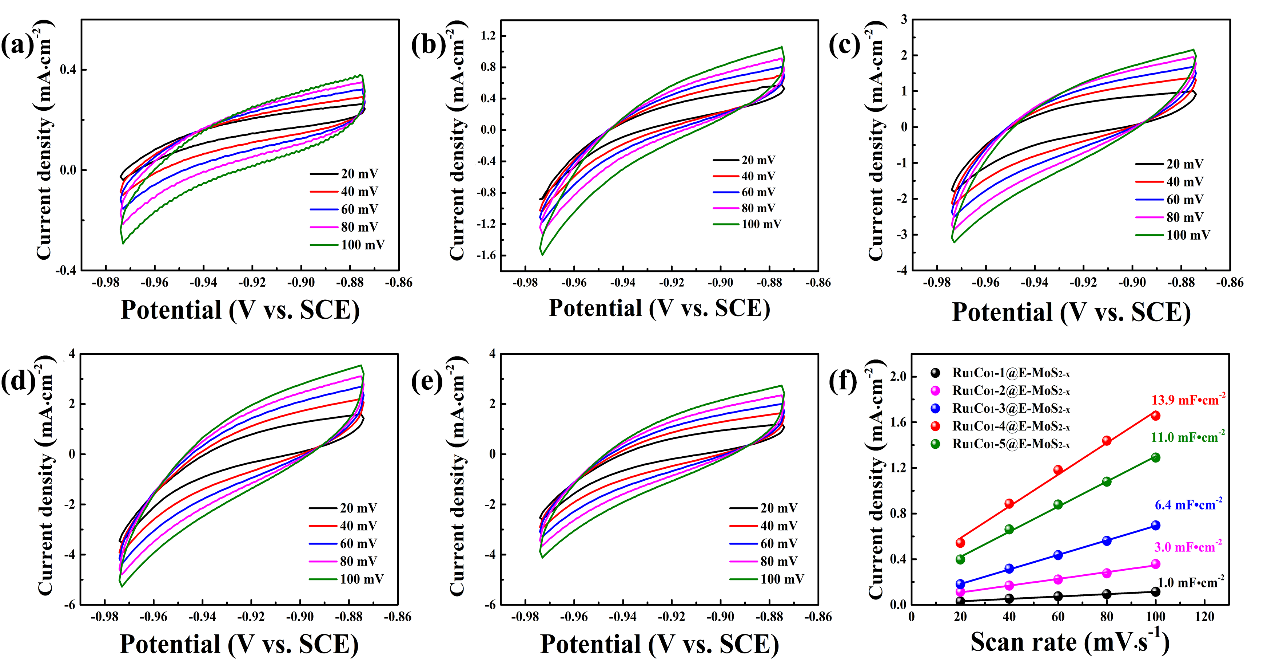


Figure S26. CV curves of the (a) Ru_1_Co_3_-4@E-MoS_2-x_ NSs, (b) Ru_1_Co_2_-4@E-MoS_2-x_ NSs, (c) Ru_1_Co_1_-4@E-MoS_2-x_ NSs, (d) Ru_2_Co_1_-4@E-MoS_2-x_ NSs, and (e) Ru_3_Co_1_-4@E-MoS_2-x_ NSs in 1.0 M KOH. (e) C_dl_ values of these samples in 1.0 M KOH.


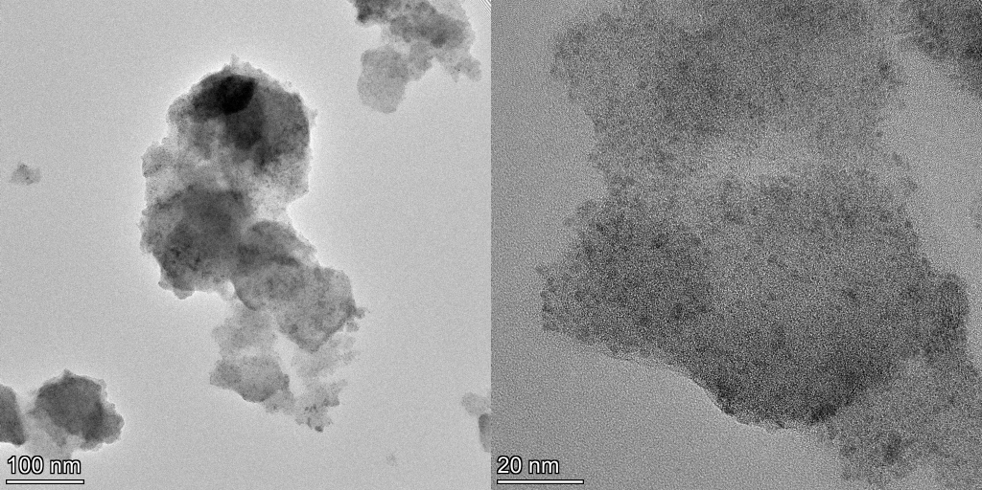


Figure S27. TEM images of Ru_2_Co_1_-4@E-MoS_2-x_ NSs.


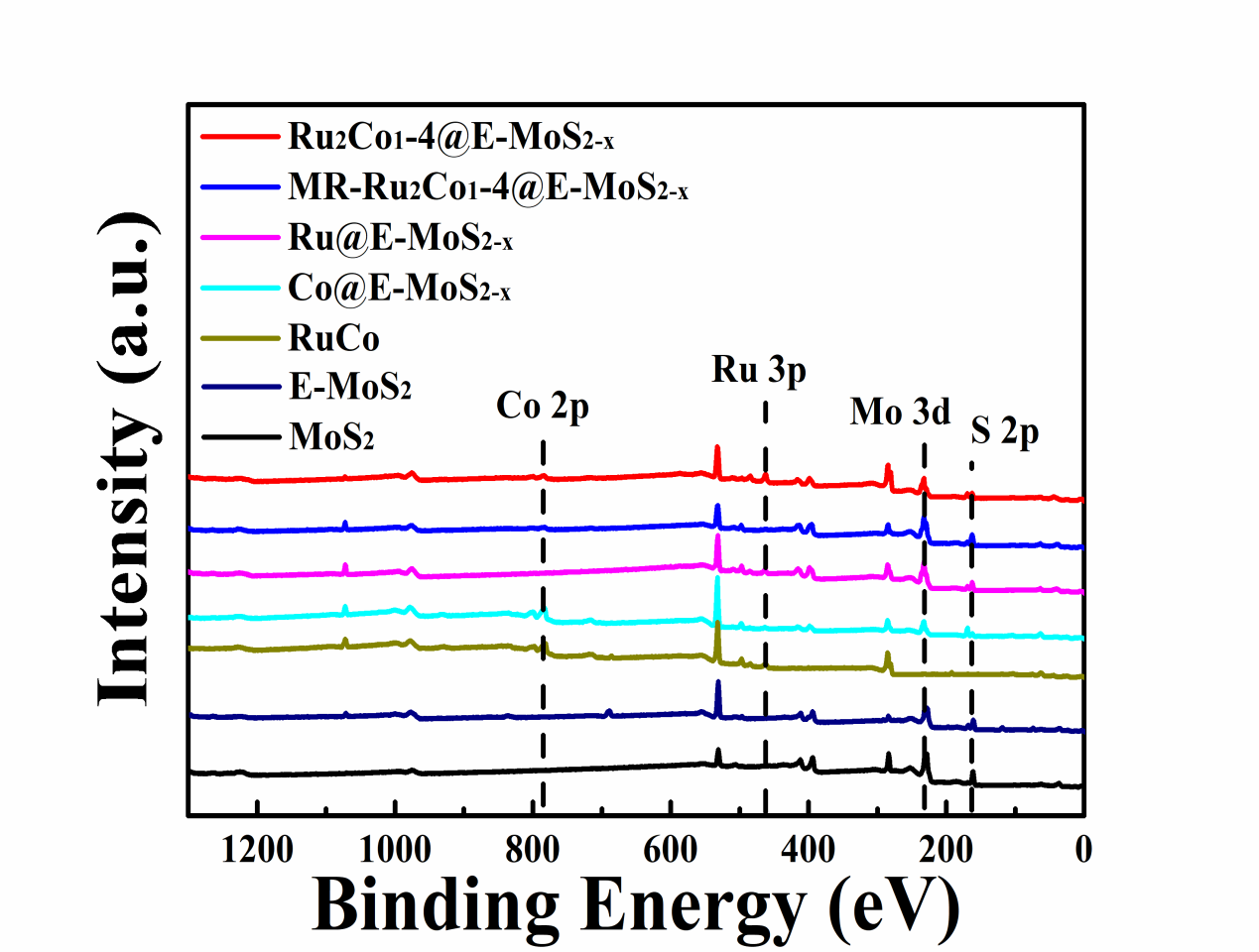


Figure S28. (a) XPS spectra of Ru_2_Co_1_-4@E-MoS_2-x_ NSs and control catalysts.


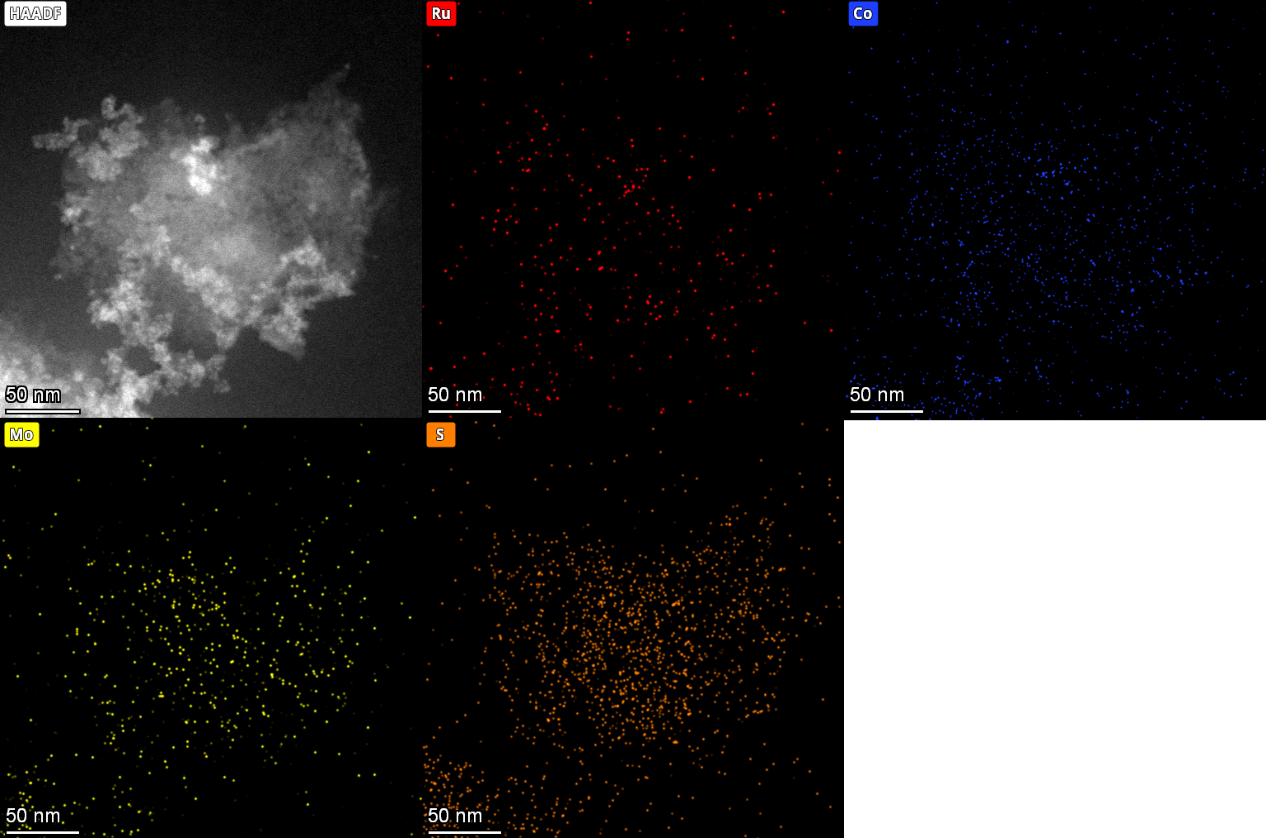


Figure S29. (a) EDX elemental mapping image of MR-Ru_2_Co_1_-4@E-MoS_2-x_ NSs.


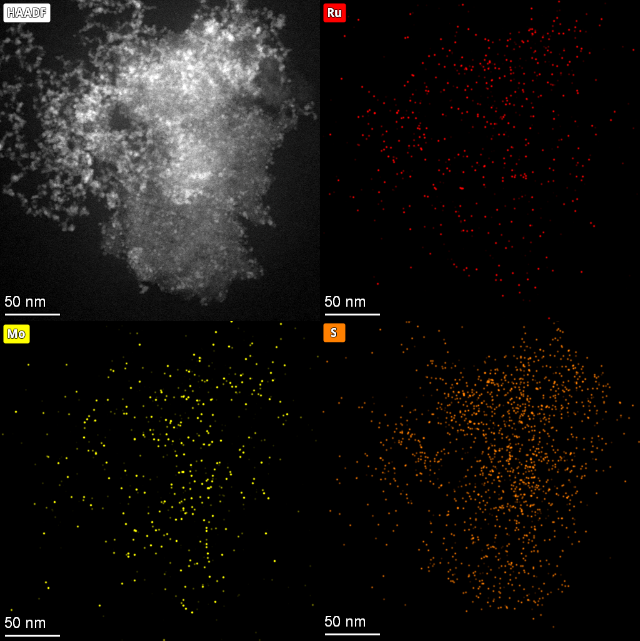


Figure S30. (a) EDX elemental mapping image of Ru@E-MoS_2-x_ NSs.


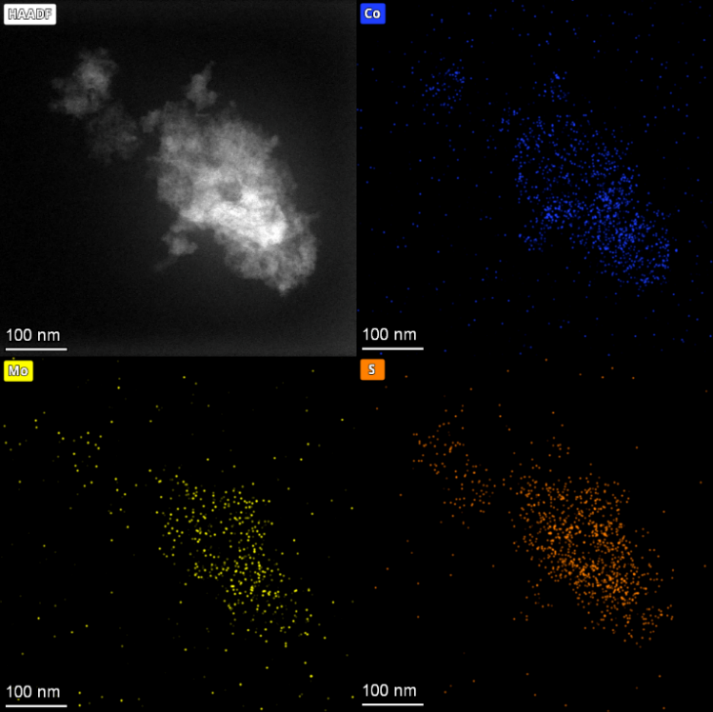


Figure S31. (a) EDX elemental mapping image of Co@E-MoS_2-x_ NSs.


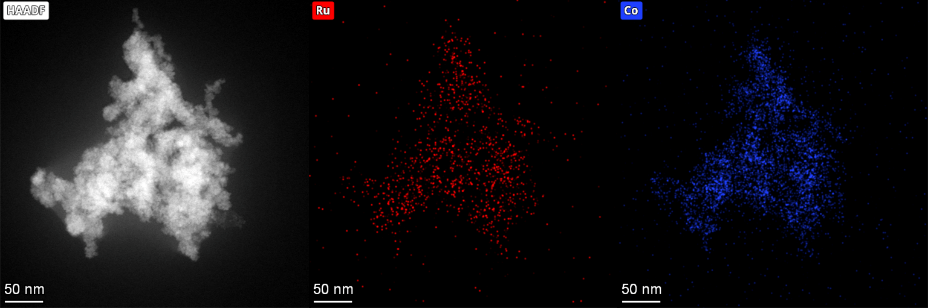


Figure S32. (a) EDX elemental mapping image of RuCo alloy.





Figure S33. Mass activity curves of Ru_2_Co_1_-4@E-MoS_2-x_ NSs and control catalysts in 0.5 M H_2_SO_4_.


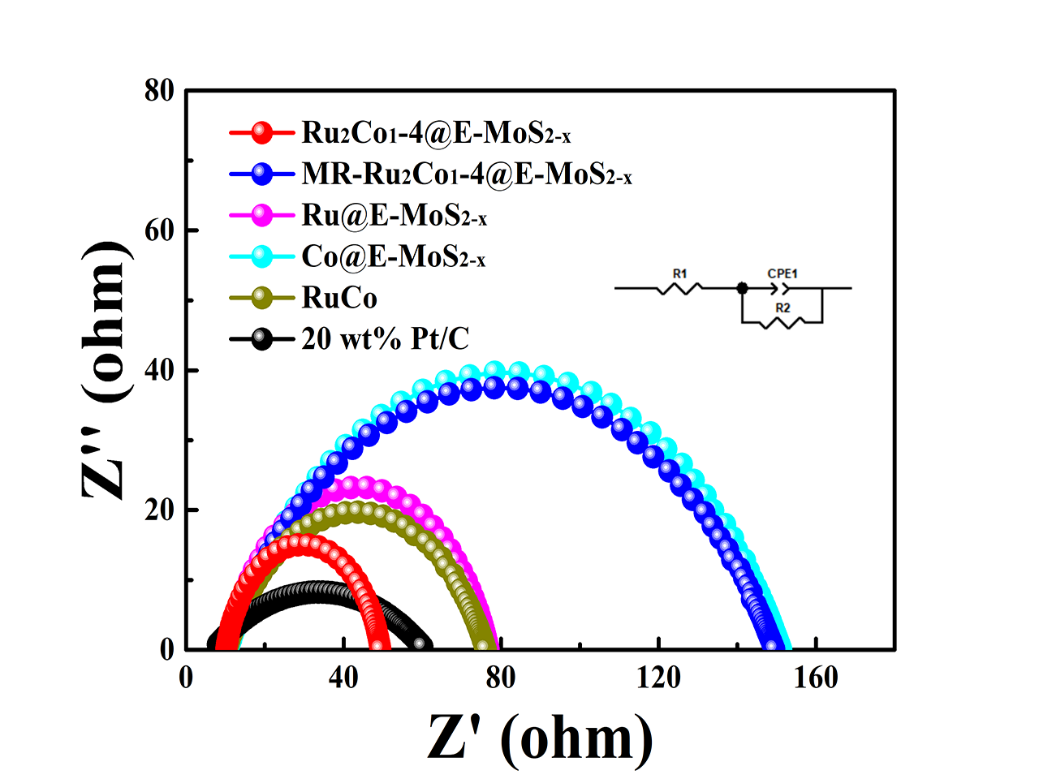


Figure S34. Nyquist plots of Ru_2_Co_1_-4@E-MoS_2-x_ NSs and control catalysts in 0.5 M H_2_SO_4_.





Figure S35. LSV curves over a wide current density range of Ru_2_Co_1_-4@E-MoS_2-x_ NSs and control catalysts in 0.5 M H_2_SO_4_.





Figure S36. Mass activity curves of Ru_2_Co_1_-4@E-MoS_2-x_ NSs and control catalysts 1.0 M KOH.


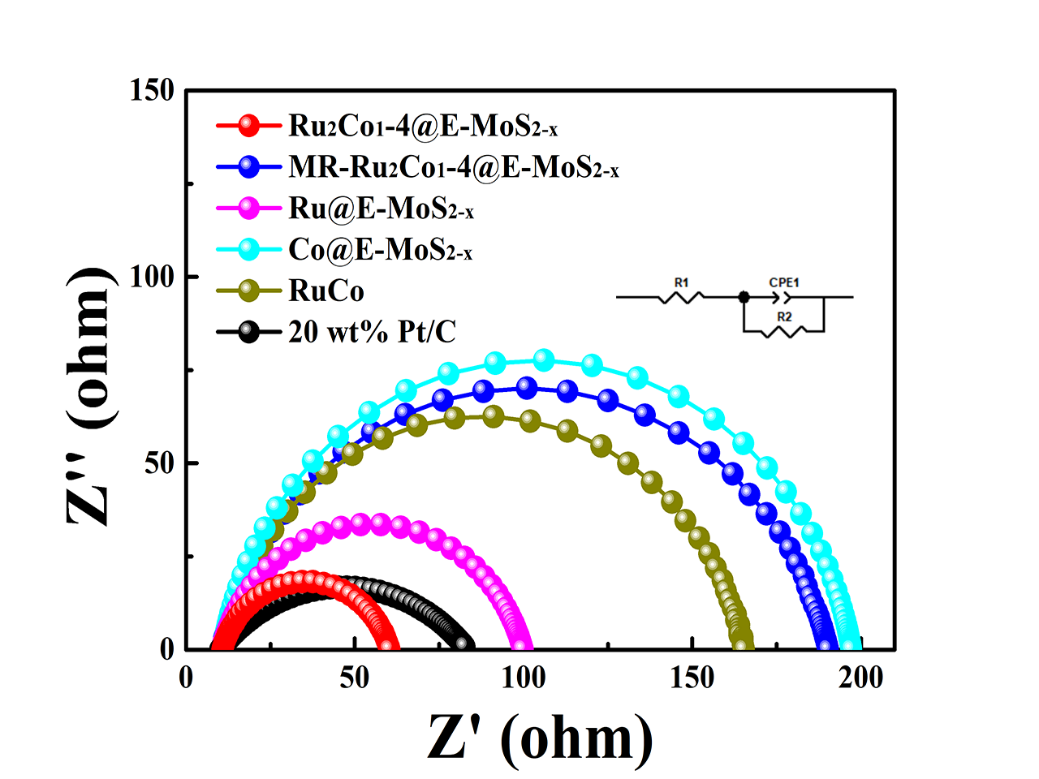


Figure S37. Nyquist plots of Ru_2_Co_1_-4@E-MoS_2-x_ NSs and control catalysts 1.0 M KOH.


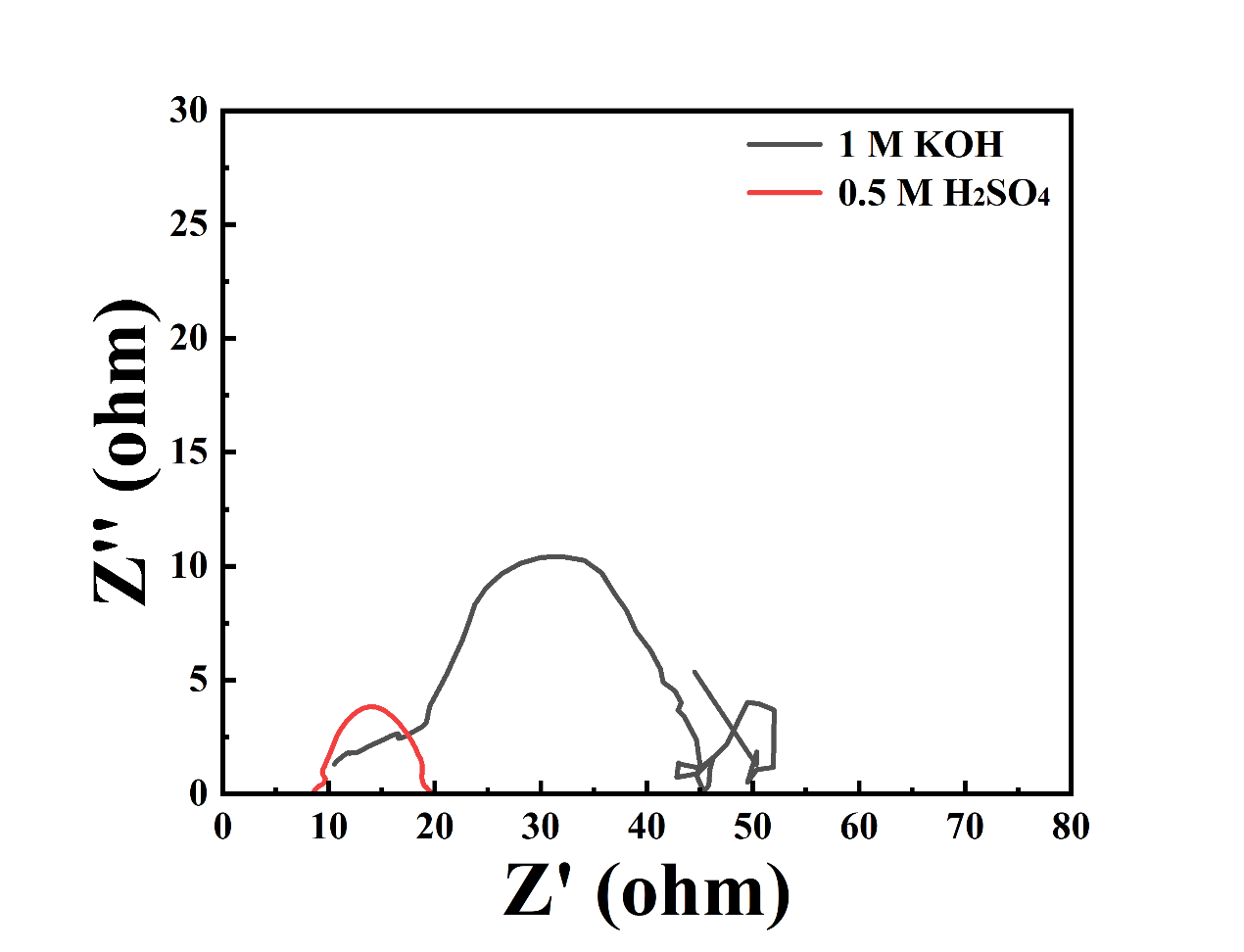


Figure S38. Nyquist plots of Ru_2_Co_1_-4@E-MoS_2-x_ NSs in 0.5 M H_2_SO_4_ and 1.0 M KOH.

Table S6. The comparison of the catalytic HER activities of Ru_2_Co_1_-4@E-MoS_2-x_ NSs with other reported catalysts in 0.5 M H_2_SO_4_ solution.

| Catalyst | η_10_ (mV) | Tafel Slope (mV·dec^-1^) | Reference |
| --- | --- | --- | --- |
| Ru_2_Co_1_-4@E-MoS_2-x_ | 31 | 29 | This work |
| 20 wt% Pt/C | 45 | 49 | This work |
| NiFe-MoS_2_ | 67 | 26.8 | ^[8]^ |
| Ru@1T-MoS_2_-MXene | 44 | 47 | ^[9]^ |
| Ru@SvMoS_2_ | 60 | 61.97 | ^[10]^ |
| Ru_2_@MoS_2_-85%/CFP | 54 | 49 | ^[11]^ |
| 2.46 nm-Ru@MoS_2_ | 53 | 86.04 | ^[12]^ |
| 4.5%-RuMoS_2_G | 36 | 74 | ^[13]^ |
| A-MoS_2_-Ni_3_S_2_-NF | 145 | 79.9 | ^[14]^ |
| m-Pt@MoS_2_ | 47 | 32 | ^[15]^ |
| Pt_1%_-CoMoS_2_/C. | 118 | 68 | ^[16]^ |
| Pd_x_S_y_/1T-MoS_2_ | 78 | 39.8 | ^[4a]^ |

Table S7. The comparison of the catalytic HER activities of Ru_2_Co_1_-4@E-MoS_2-x_ NSs with other reported catalysts in 1.0 M KOH solution.

| Catalyst | η_10_ (mV) | Tafel Slope (mV·dec^-1^) | Reference |
| --- | --- | --- | --- |
| Ru_2_Co_1_-4@E-MoS_2-x_ | 36 | 31 | This work |
| 20 wt% Pt/C | 53 | 58 | This work |
| Ru_0.10_@2H-MoS_2_ | 51 | 64.9 | ^[17]^ |
| MSOR_1_ | 43 | 63.1 | ^[18]^ |
| Co-1T-MoS_2_-bye | 118 | 83 | ^[19]^ |
| MoS_2_/CoFe@NC | 172 | 111.6 | ^[20]^ |
| Ru@1T-MoS_2_-MXene | 42 | 38 | ^[9]^ |
| Ru@SvMoS_2_ | 125 | 94.66 | ^[10]^ |
| Ru MIs-MoS_2_ | 17 | 63 | ^[21]^ |
| Ru@MoS_2_-85%/CFP | 19 | 29 | ^[11]^ |
| Er-MoS_2_/NC | 55 | 76 | ^[22]^ |
| 4.5%-RuMoS_2_G | 60 | 38 | ^[13]^ |
| Ru_1_@D-MoS_2_ | 107 | 96 | ^[23]^ |
| CoNi@MoS_2_-Pd_SA_Ru_SA_ | 89 | 51 | ^[24]^ |
| A-MoS_2_-Ni_3_S_2_-NF | 95 | 107.5 | ^[14]^ |
| Pt-MoS_2_-Co@CHNF | 91 | 78 | ^[25]^ |


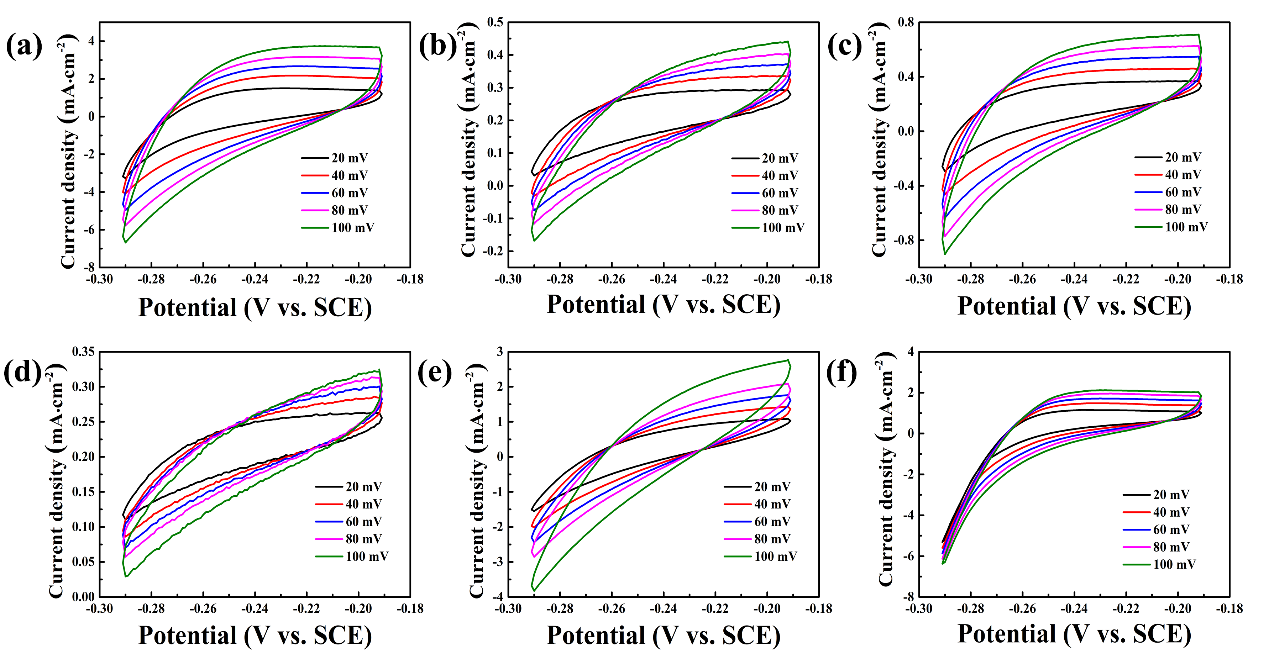


Figure S39. CV curves of the (a) Ru_2_Co_1_-4@E-MoS_2-x_ NSs, (b) MR-Ru_2_Co_1_-4@E-MoS_2-x_ NSs, (c) Ru@E-MoS_2-x_ NSs, (d) Co@E-MoS_2-x_ NSs, (e) RuCo alloy and (f) 20 wt% Pt/C in 0.5 M H_2_SO_4_.


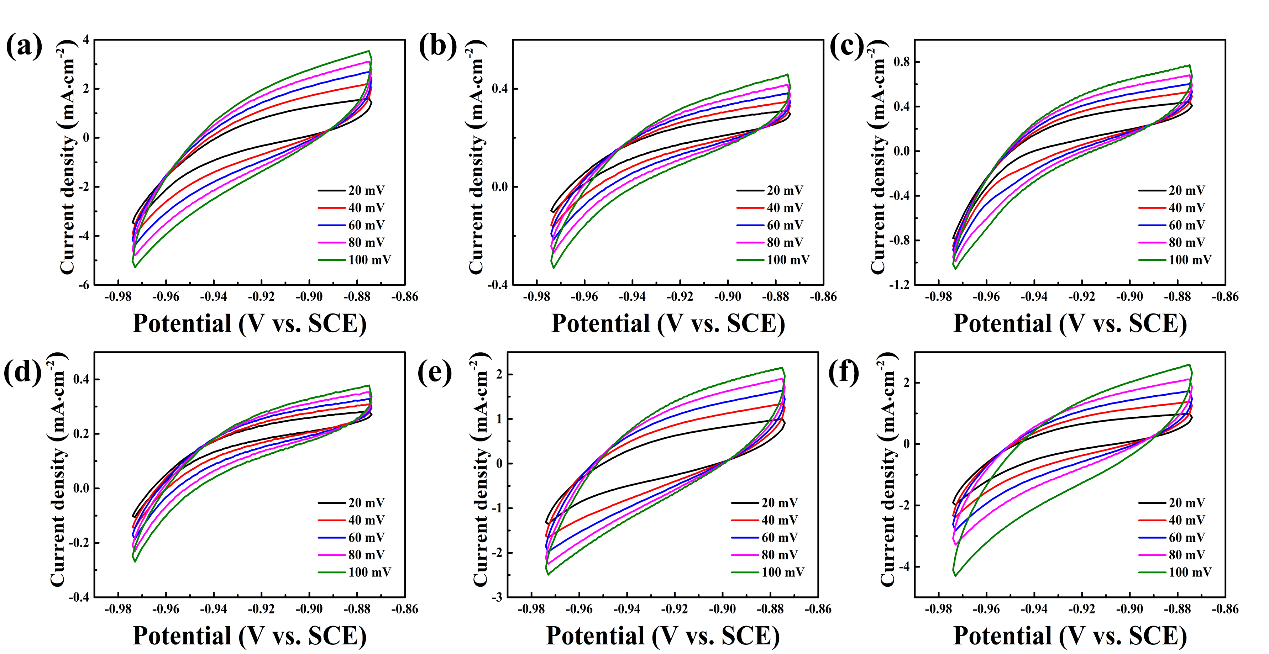


Figure S40. CV curves of the (a) Ru_2_Co_1_-4@E-MoS_2-x_ NSs, (b) MR-Ru_2_Co_1_-4@E-MoS_2-x_ NSs, (c) Ru@E-MoS_2-x_ NSs, (d) Co@E-MoS_2-x_ NSs, (e) RuCo alloy and (f) 20 wt% Pt/C in 1.0 M KOH.


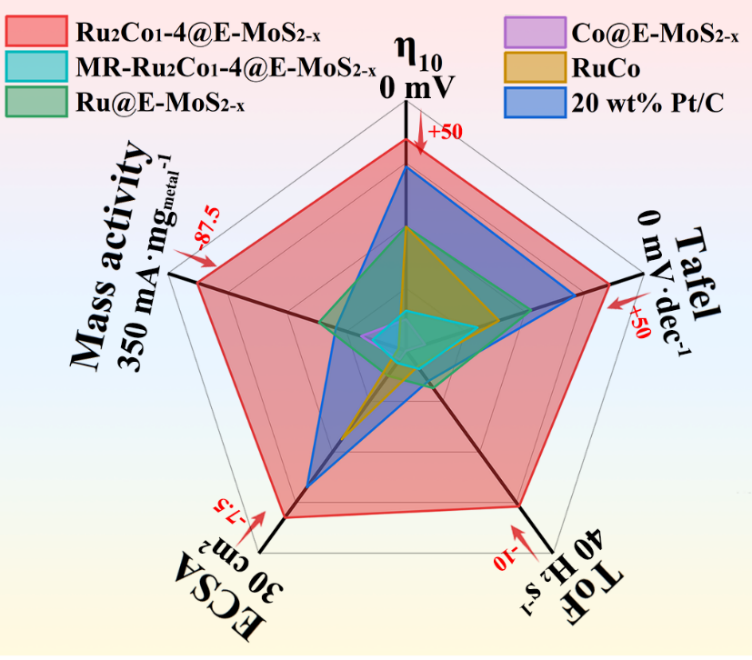


Figure S41. Comparison of 𝜂_10_, ECSA, Tafel slope, TOF, and mass activity of different catalysts in 0.5 M H_2_SO_4_.

Table S8. Comparison of 𝜂_10_, Tafel slope, j_0_, ECSA, and mass activity of different catalysts in 0.5 M H_2_SO_4_.

|  | η_10_ (mv) | Tafel slope (mV·dec^-1^) | j_0_ (mA·cm^-2^) | ESCA (cm^2^) | Mass activity @ 30 mV (mA·mg_metal_^-1^) |
| --- | --- | --- | --- | --- | --- |
| Ru_2_Co_1_-4@E-MoS_2-x_ | 31 | 29 | 1.67 | 35.9 | 307 |
| MR-Ru_2_Co_1_-4@E-MoS_2-x_ | 167 | 124 | 0.57 | 1.3 | 32 |
| Ru@E-MoS_2-x_ | 118 | 103 | 0.73 | 3.7 | 80 |
| Co@E-MoS_2-x_ | 183 | 153 | 0.59 | 0.4 | 53 |
| RuCo | 111 | 132 | 1.51 | 13.0 | 9 |
| 20 wt% Pt/C | 45 | 49 | 1.55 | 16.5 | 95 |

Table S9. Concentrations of Ru, Co, Mo, and S elements in Ru_2_Co_1_-4@E-MoS_2-x_ NSs and MR- Ru_2_Co_1_-4@E-MoS_2-x_ NSs.

|  | Ru | Co | Mo | S |
| --- | --- | --- | --- | --- |
| Ru_2_Co_1_-4@E-MoS_2-x_ | 14.86 | 9.39 | 28.18 | 47.57 |
| MR-Ru_2_Co_1_-4@E-MoS_2-x_ | 2.55 | 5.64 | 35.84 | 55.97 |





Figure S42. XPS spectra of Ru_2_Co_1_-4@E-MoS_2-x_ NSs and MR- Ru_2_Co_1_-4@E-MoS_2-x_ NSs.


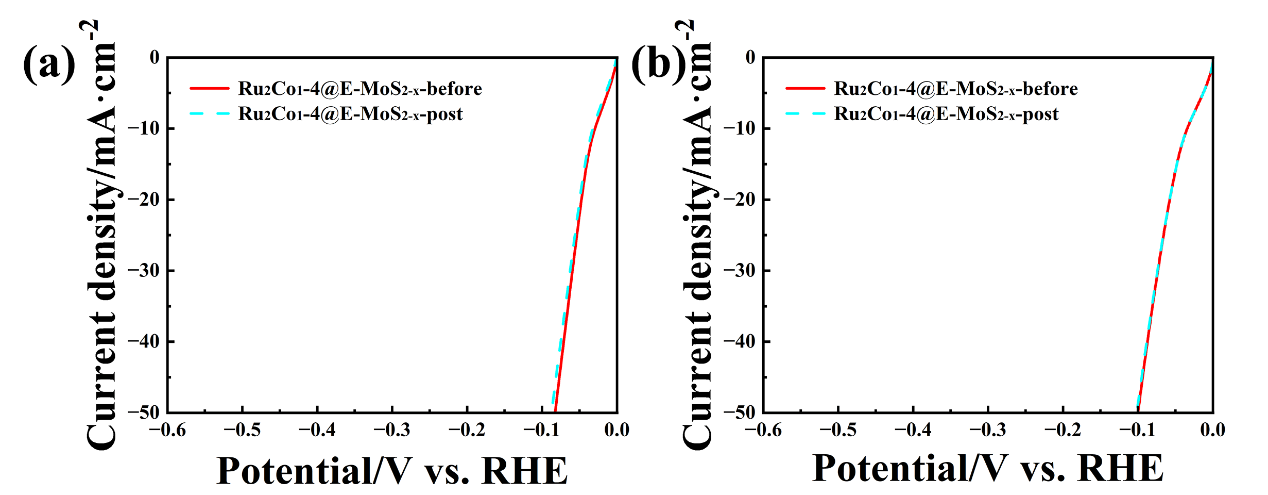


Figure S43. LSV curves of the Ru_2_Co_1_-4@E-MoS_2-x_ NSs before and after long-term stability test in (a) 0.5 M H_2_SO_4_ and (b) 1.0 M KOH.


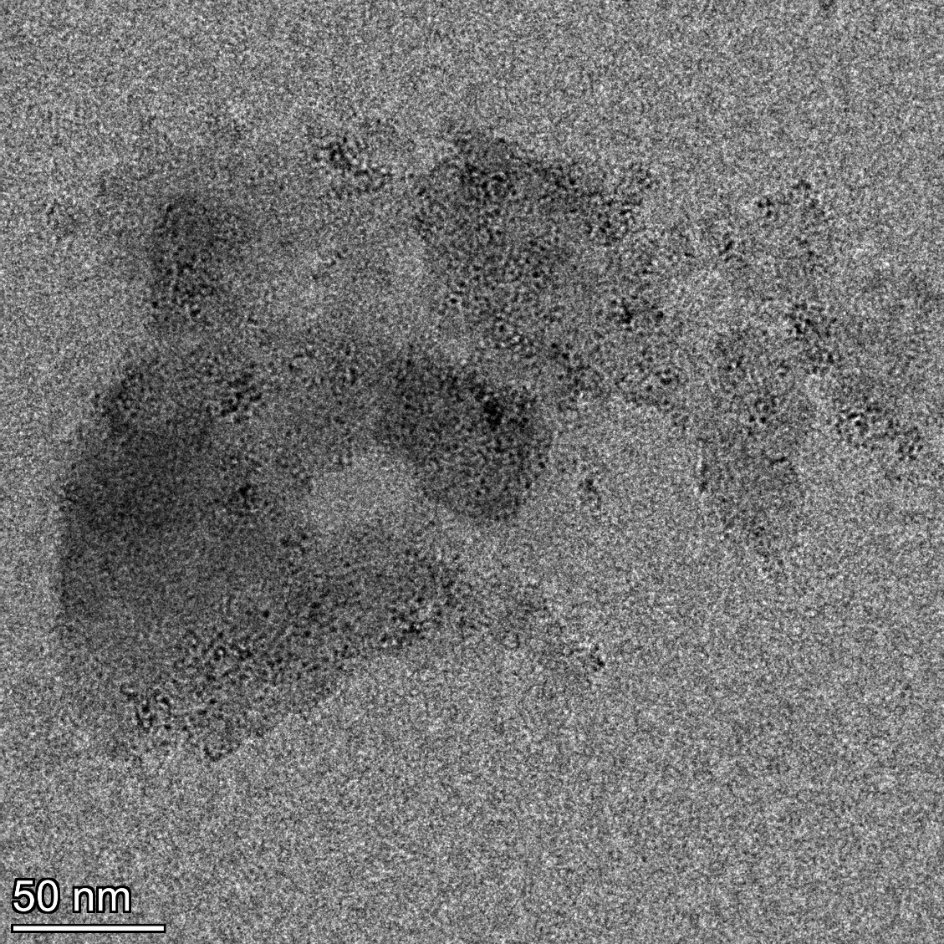


Figure S44. TEM images of the Ru_2_Co_1_-4@E-MoS_2-x_ NSs after long-term stability test in 0.5 M H_2_SO_4_.


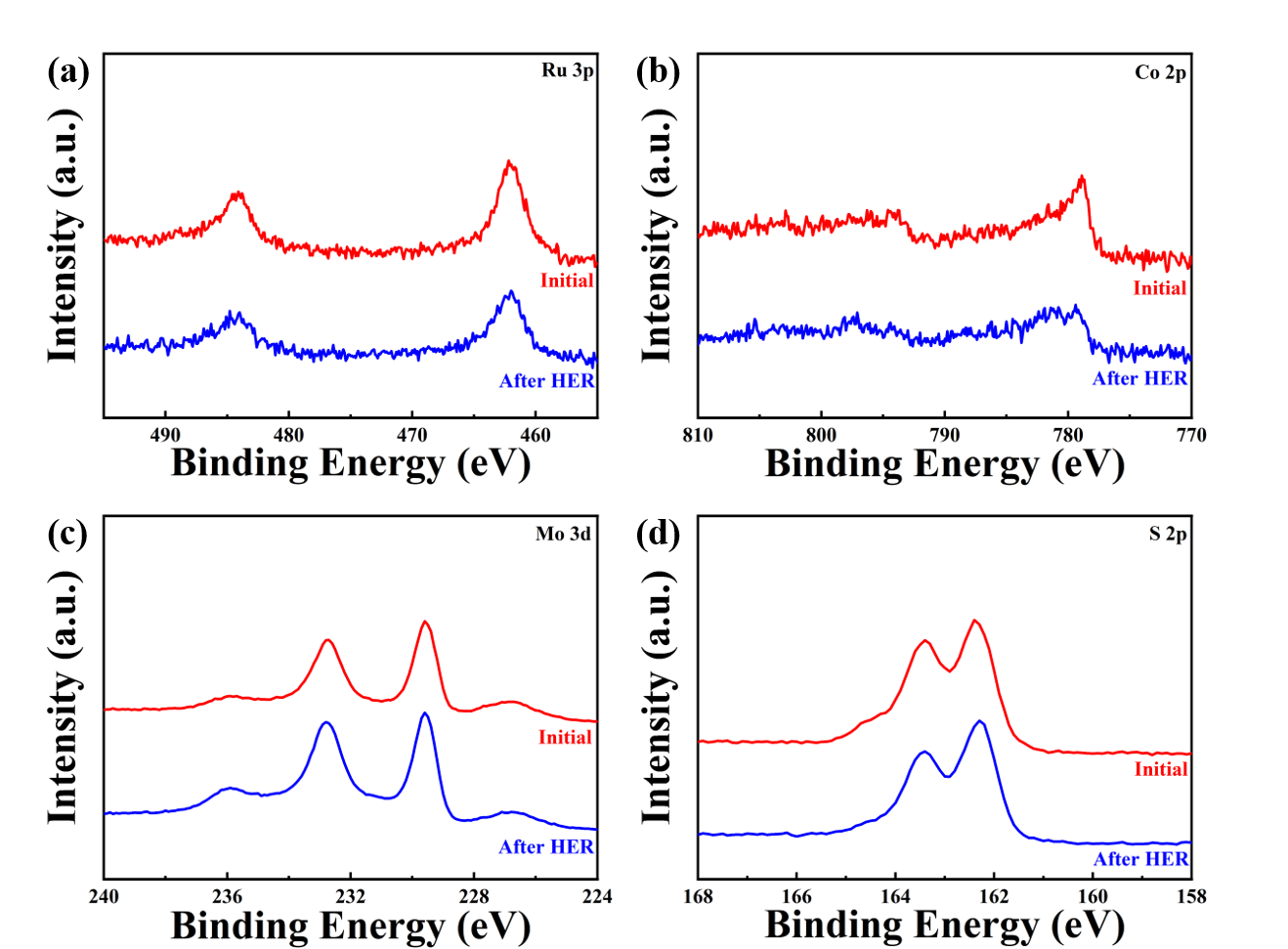


Figure S45. (a) Ru 3p, (b) Co 2p, (c) Mo 3d, and (d) S 2p high-resolution XPS spectra of Ru_2_Co_1_-4@E-MoS_2-x_ NSs before and after long-term stability test.





Figure S46. XRD patterns of Ru_2_Co_1_-4@E-MoS_2-x_ NSs before and after long-term stability test.


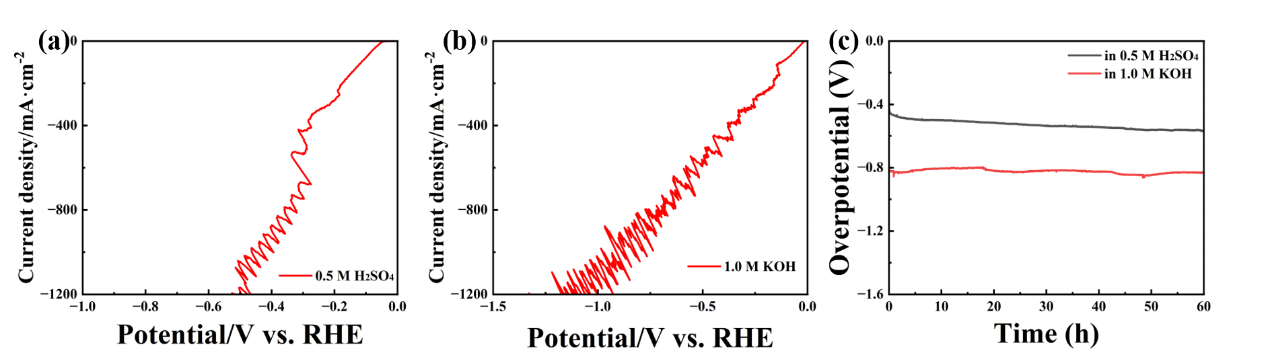


Figure S47. LSV curves of Ru_2_Co_1_-4@E-MoS_2-x_ NSs in (a) 0.5 M H_2_SO_4_ and (d) 1.0 M KOH. Chronopotentiometry curves of Ru_2_Co_1_-4@E-MoS_2-x_ NSs at 1 A·cm^-2^ in 0.5 M H_2_SO_4_ and 1.0 M KOH.


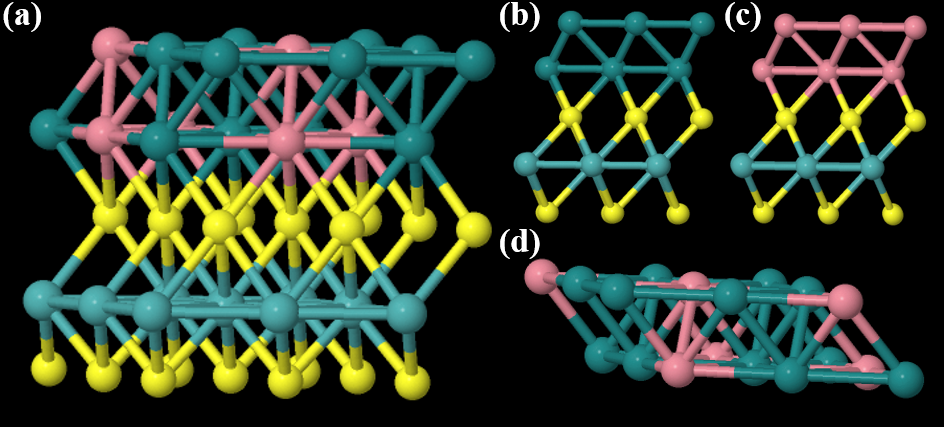


Figure S48. Atomic model structures of (a) Ru_2_Co_1_-4@E-MoS_2-x_ NSs, (b) Ru@E-MoS_2-x_ NSs, (c) Co@E-MoS_2-x_ NSs, and (d) RuCo with H adsorption. Atomic colors: green: Ru, pink: Co, cyan: Mo, yellow: S.


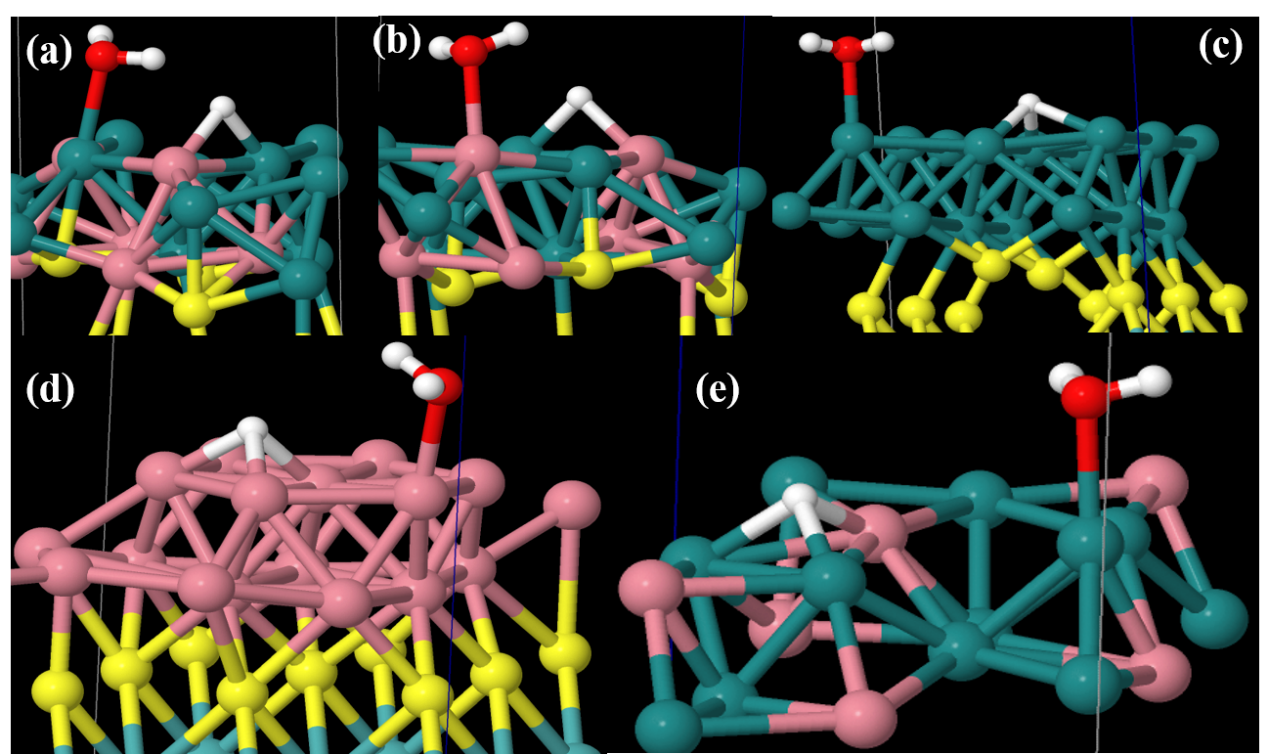


Figure S49. HER process of the Ru_2_Co_1_-4@E-MoS_2-x_ NSs in alkaline conditions.


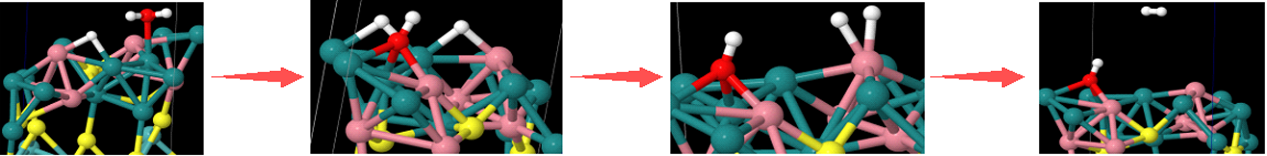


Figure S50. HER process of the Ru_2_Co_1_-4@E-MoS_2-x_ NSs in alkaline conditions.


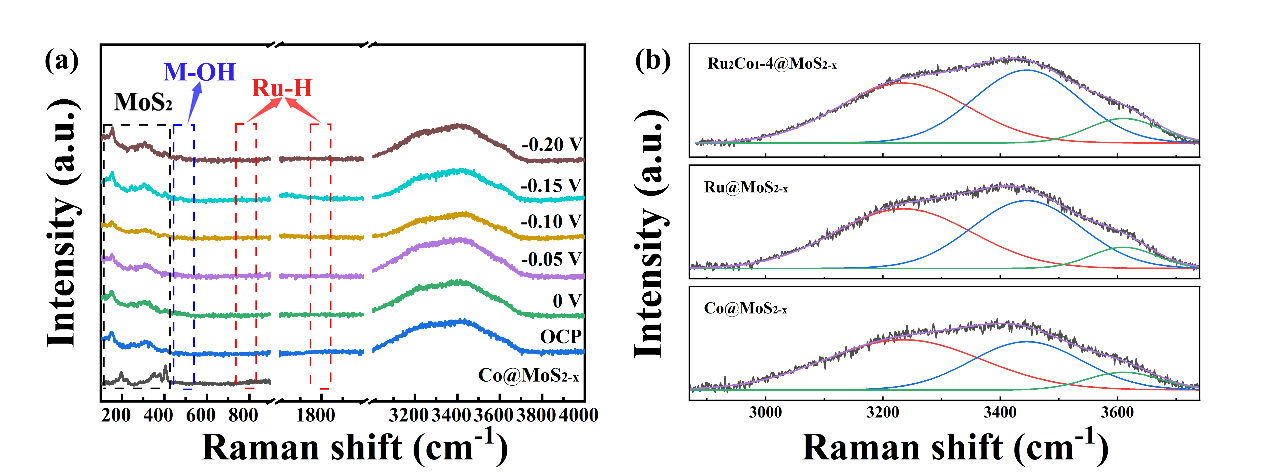


Figure S51. (a) In situ Raman spectra of HER for Co@E-MoS_2-x_ NSs in 1.0 M KOH; (b) The Raman spectrum at a potential of -0.20 V shows the distribution of interfacial water on the surfaces of different samples, where red, blue, and green correspond to the Gaussian fitting results of the three oxygen-hydrogen stretching vibration modes (O-H) of tetrahedral coordinated water, trihedral coordinated water, and free water, respectively.


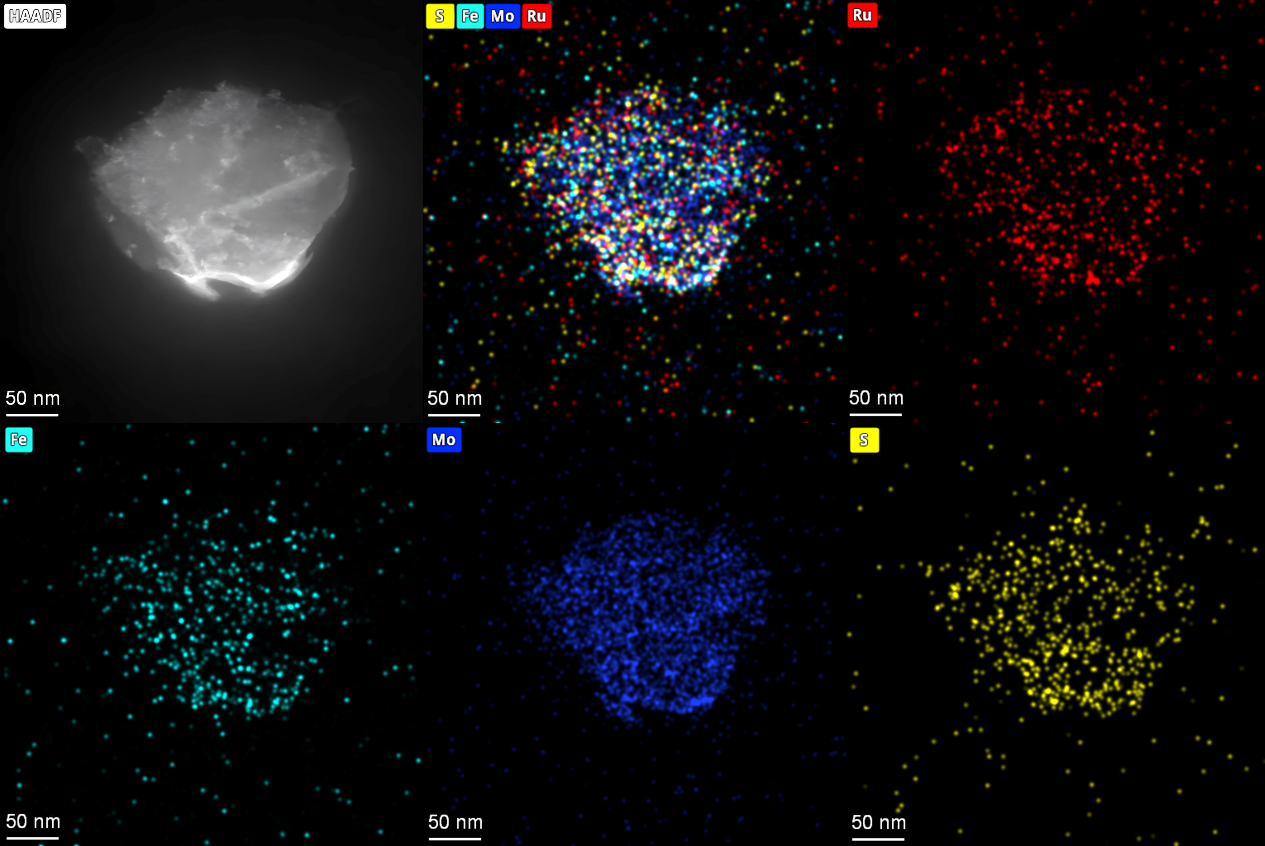


Figure S52. EDX elemental mapping image of Ru_2_Fe_1_-4@E-MoS_2-x_ NSs.


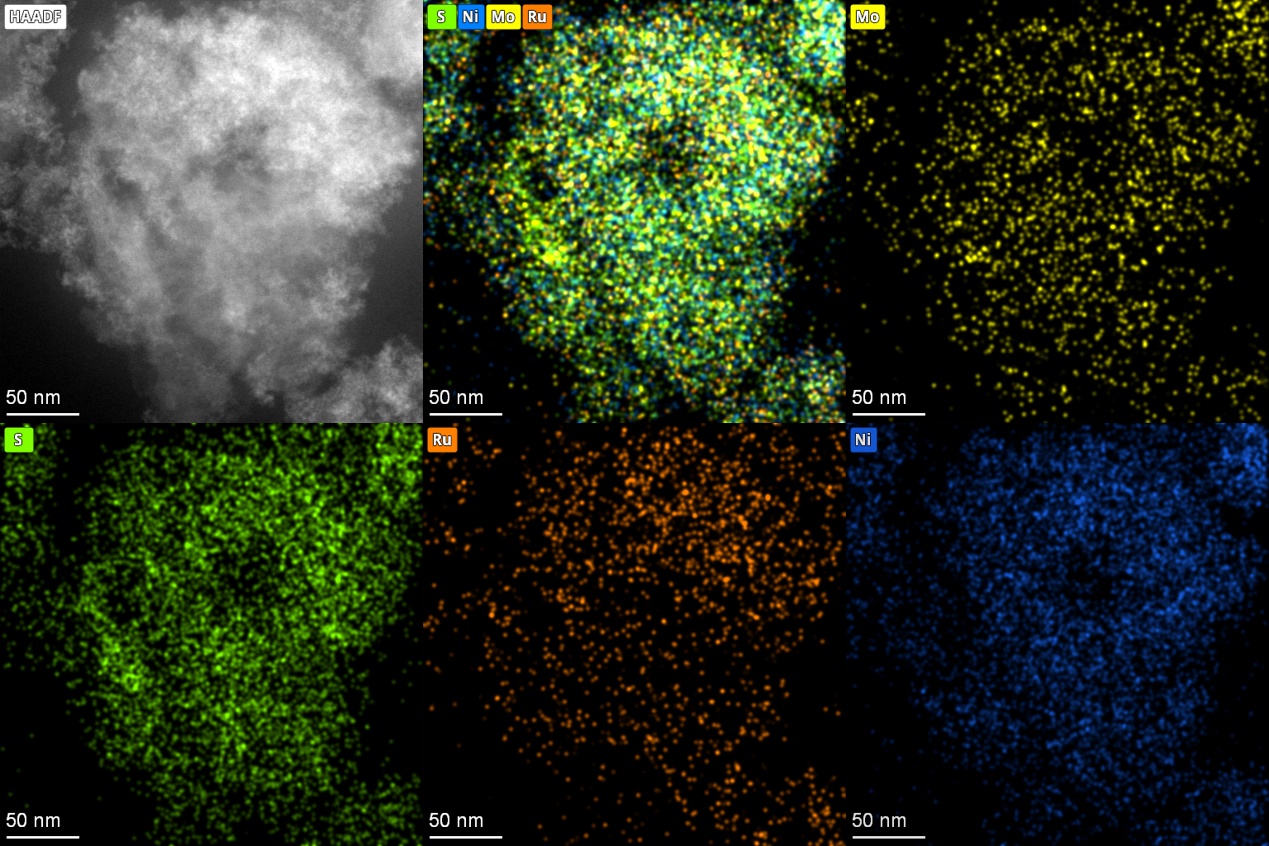


Figure S53. EDX elemental mapping image of Ru_2_Ni_1_-4@E-MoS_2-x_ NSs.


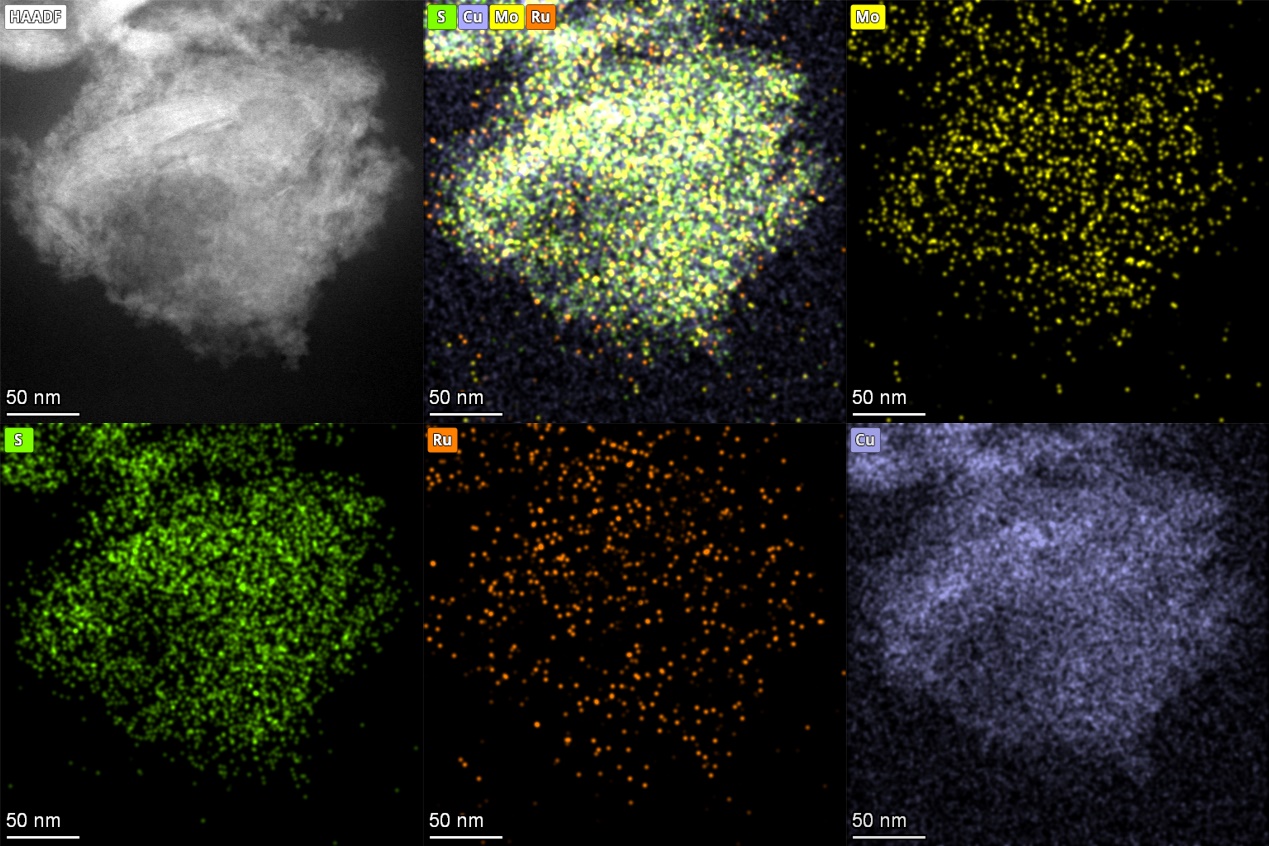


Figure S54. EDX elemental mapping image of Ru_2_Cu_1_-4@E-MoS_2-x_ NSs.

***References***

[1] H. Tang, Z. Wu, J. Zhang, R. Su, X. Zhu, Z. Dong, *Ceramics International* **2025**, 51, 3283.

[2] C. Peng, X. Zhu, J. Zhang, W. Zhao, J. Jia, Z. Wu, Z. Yu, Z. Dong, *Ultrasonics Sonochemistry* **2024**, 103.

[3] a) L.-N. Wang, X. Wu, F.-T. Wang, X. Chen, J. Xu, K.-J. Huang, *Journal of Colloid and Interface Science* **2021**, 583, 579; b) X. Yu, F. Yan, Y. Zhao, B. Geng, X. Ma, L. Wu, X. Zhang, Y. Chen, *Applied Catalysis B: Environmental* **2024**, 343.

[4] a) H. D. Mai, S. Jeong, G. N. Bae, N. M. Tran, J. S. Youn, C. M. Park, K. J. Jeon, *Advanced Energy Materials* **2023**, 13; b) L. Liu, J. Wu, L. Wu, M. Ye, X. Liu, Q. Wang, S. Hou, P. Lu, L. Sun, J. Zheng, L. Xing, L. Gu, X. Jiang, L. Xie, L. Jiao, *Nature Materials* **2018**, 17, 1108.

[5] a) T. Liu, W. Wu, X. Bai, *Ultrasonics Sonochemistry* **2024**, 105; b) J. Li, C. Hou, C. Chen, W. Ma, Q. Li, L. Hu, X. Lv, J. Dang, *ACS Nano* **2023**, 17, 10947.

[6] a) X. Wang, H. Yao, C. Zhang, C. Li, K. Tong, M. Gu, Z. Cao, M. Huang, H. Jiang, *Advanced Functional Materials* **2023**, 33; b) J. Guan, W. Chen, Y. Zhu, L. Wang, Y. Fu, B. Guo, M. Zhang, *Journal of Alloys and Compounds* **2023**, 942; c) J. Li, Y. Tan, M. Zhang, W. Gou, S. Zhang, Y. Ma, J. Hu, Y. Qu, *ACS Energy Letters* **2022**, 7, 1330.

[7] Y. He, F. Yan, X. Zhang, C. Zhu, Y. Zhao, B. Geng, S. Chou, Y. Xie, Y. Chen, *Advanced Energy Materials* **2023**, 13.

[8] Z. Jiang, W. Zhou, C. Hu, X. Luo, W. Zeng, X. Gong, Y. Yang, T. Yu, W. Lei, C. Yuan, *Advanced Materials* **2023**, 35.

[9] G. Li, T. Sun, H. J. Niu, Y. Yan, T. Liu, S. Jiang, Q. Yang, W. Zhou, L. Guo, *Advanced Functional Materials* **2023**, 33.

[10] T. Lin, R. Xu, T. Bo, Y. Hu, Y. Liu, W. Zhou, *Applied Surface Science* **2024**, 671.

[11] C. Xu, H. Yu, H. Huang, W. Huang, S. Li, Y. Cao, H. Lu, G. Li, Y. Li, X. Li, Y. Zhang, W. Chen, *Chemical Engineering Journal* **2024**, 489.

[12] T. Lin, R. Xu, Y. Hu, J. Wang, Y. Liu, W. Zhou, *International Journal of Hydrogen Energy* **2024**, 68, 688.

[13] S. E. Islam, D.-R. Hang, C.-T. Liang, K. H. Sharma, H.-C. Huang, M. M. C. Chou, *Chemical Engineering Journal* **2024**, 488.

[14] M. Hu, Y. Qian, S. Yu, Q. Yang, Z. Wang, Y. Huang, L. Li, *Small* **2023**, 20.

[15] J. Wang, W. Zang, X. Liu, J. Sun, S. Xi, W. Liu, Z. Kou, L. Shen, J. Wang, *Small* **2024**, 20.

[16] L. A. Zavala, K. Kumar, V. Martin, F. Maillard, F. Maugé, X. Portier, L. Oliviero, L. Dubau, *ACS Catalysis* **2023**, 13, 1221.

[17] J. Wang, W. Fang, Y. Hu, Y. Zhang, J. Dang, Y. Wu, B. Chen, H. Zhao, Z. Li, *Applied Catalysis B: Environmental* **2021**, 298.

[18] Y. Zhang, T. Yang, J. Li, Q. Zhang, B. Li, M. Gao, *Advanced Functional Materials* **2022**, 33.

[19] H. J. Liu, S. Zhang, Y. M. Chai, B. Dong, *Angewandte Chemie International Edition* **2023**, 62.

[20] W. Ma, W. Li, H. Zhang, Y. Wang, *International Journal of Hydrogen Energy* **2023**, 48, 22032.

[21] X. Li, S. Han, Z. Qiao, X. Zeng, D. Cao, J. Chen, *Chemical Engineering Journal* **2023**, 453.

[22] N.-P. Li, L. Zhang, H. Zhang, F.-F. Min, L. Wang, S. Toan, X.-J. Liu, G.-Z. Hu, *Rare Metals* **2023**, DOI: 10.1007/s12598-023-02409-7.

[23] C. Lang, W. Jiang, C. J. Yang, H. Zhong, P. Chen, Q. Wu, X. Yan, C. L. Dong, Y. Lin, L. Ouyang, Y. Jia, X. Yao, *Small* **2023**, 19.

[24] M. Islam, T. H. Nguyen, D. T. Tran, V. A. Dinh, N. H. Kim, J. H. Lee, *ACS Sustainable Chemistry & Engineering* **2023**, 11, 6688.

[25] C. Sun, C. Wang, G. Han, Y. Zhang, H. Zhao, *Chemical Engineering Journal* **2024**, 496.
